# Recovery of novel association loci in *Arabidopsis thaliana* and *Drosophila melanogaster* through leveraging INDELs association and integrated burden test

**DOI:** 10.1371/journal.pgen.1007699

**Published:** 2018-10-16

**Authors:** Baoxing Song, Richard Mott, Xiangchao Gan

**Affiliations:** 1 Max Planck Institute for Plant Breeding Research, Köln, Germany; 2 UCL Genetics Institute, University College London, London United Kingdom; The University of North Carolina at Chapel Hill, UNITED STATES

## Abstract

Short insertions, deletions (INDELs) and larger structural variants have been increasingly employed in genetic association studies, but few improvements over SNP-based association have been reported. In order to understand why this might be the case, we analysed two publicly available datasets and observed that 63% of INDELs called in *A*. *thaliana* and 64% in *D*. *melanogaster* populations are misrepresented as multiple alleles with different functional annotations, i.e. where the same underlying variant is represented by inconsistent alignments leading to different variant calls. To address this issue, we have developed the software Irisas to reclassify and re-annotate these variants, which we then used for single-locus tests of association. We also integrated them to predict the functional impact of SNPs, INDELs, and structural variants for burden testing. Using both approaches, we re-analysed the genetic architecture of complex traits in *A*. *thaliana* and *D*. *melanogaster*. Heritability analysis using SNPs alone explained on average 27% and 19% of phenotypic variance for *A*. *thaliana* and *D*. *melanogaster* respectively. Our method explained an additional 11% and 3%, respectively. We also identified novel trait loci that previous SNP-based association studies failed to map, and which contain established candidate genes. Our study shows the value of the association test with INDELs and integrating multiple types of variants in association studies in plants and animals.

## Author summary

In this study, we develop a method for testing multiple types of variants in genome-wide association studies. We show that a multi-allelic artefact caused by inconsistent alignments was a key obstacle for testing association of insertion and deletion polymorphisms (INDELs) and for integrated association methods, such as burden testing. To address this problem, we developed the software Irisas that synchronizes variants and integrates the impact of SNPs, INDELs and structural variants for burden testing. We re-analysed two publicly available datasets with multiple traits in *A*. *thaliana* and *D*. *melanogaster*. We identified novel loci that contain well-established candidate genes that SNP-based GWAS failed to detect; INDEL-specific QTLs generally have weak local linkage disequilibrium (LD) with nearby SNPs. We showed by simulation that multiple independent loss-of-function common-allele SNPs/INDELs were challenging for direct association tests but demonstrated that they could be recovered by integrated burden testing.

## Introduction

Identifying the causal loci underlying phenotypic variance is a fundamental biological challenge. Genome-wide association studies (GWAS) is a widely used and effective methodology, which tests a genome-wide set of genetic variants in different individuals for association with trait variation. Typically, single-nucleotide polymorphisms (SNP) are genotyped, either by array, re-sequencing or imputation, and then used as markers to identify loci associated with the trait. However, the total variance explained by summing mapped quantitative trait loci (QTLs) is usually much less than the overall heritability estimated from genome-wide genetic relationships [1–3]. Many explanations have been proposed, such as attributing it to variants of small effect or of low allele frequency [4].

SNP-based GWAS rely on linkage disequilibrium (LD) to tag nearby causal variants, which might include other SNPs, insertions or deletions (INDELs) or structural variants (SVs). Though INDELs and SVs have been increasingly employed for GWAS [5–7], it is unclear whether explicitly testing those variants imperfectly tagged by SNPs will increase power. For example, in *A*. *thaliana*, a 16bp insertion and 345bp complex deletion (where 376bp have been replaced with 31bp) in the *FRIGIDA* (*FRI*) gene both have been linked to flowering time [8], an important adaptive trait in plants. Interestingly, when these two variants were genotyped using dideoxy sequencing and added to a panel of array-genotyped SNPs for genome-wide significance test, neither of them reached genome-wide significance in a traditional GWAS [9]. It is thus important to understand why such established causal INDELs and SVs were missed by GWAS.

To date, there has been a lack of methodological investigations of INDELs and SVs in GWAS. These types of variants have usually been treated and encoded in the same way as SNPs, although it is well known that it is more difficult calling INDELs and SVs accurately from short-read sequence data [10]. It is unclear whether hidden factors or different methods could improve power and thus recover novel loci. However, it has been observed that even for very short INDELs, where current sequence technology can provide fairly good accuracy in variant calling, encoding them in a consistent way for GWAS is not trivial [11]. A related issue is that of complex substitutions, i.e. where a segment of DNA is replaced by another segment; these substitutions can be represented either as integrated complex events or by combinations of many smaller atomic SNPs and INDELs. In some cases their phenotypic effects will depend on their integrated context rather than on their constituent parts. This line of argument leads to the idea of an integrated burden test, which may be particularly relevant within protein coding sequence. This is supported by our earlier observations in *Arabidopsis thaliana*, that changes around and within coding sequences should be considered holistically in order to avoid erroneously predicting deleterious changes in which the effect of one variant was compensated by another change nearby [12].

In this paper, we show that many INDELs are misclassified as multi-allelic with potentially different functional annotations, which undermines the association. We have developed the software Irisas (**I**ntegrated **re**g**i**on-based variant **s**ynchronization and **a**nnotation for association **s**tudies) to reclassify and re-annotate these variants. In addition, we propose a robust measure that integrates the predicted functional impact of SNPs, INDELs, and SVs for burden test. The single-locus association test using our synchronized INDELs and integrated burden test explained a large proportion of phenotypic variance additionally. We thereby map novel loci that SNP-based GWAS have failed to associate and which contain established candidate genes. Collectively, our work demonstrates a reliable framework to leverage INDELs for GWAS, and establishes the value of integrated analysis of multiple types of variants in association studies in plants and animals.

## Results

### Variant synchronization and integrated burden test for GWAS

We re-analysed 106 *A*. *thaliana* phenotypes [9] and 153 *D*. *melanogaster* phenotypes measured in DGRP inbred lines [13, 14]. The *A*. *thaliana* dataset contained 177 inbred accessions from the 1001 genomes project [15] (S1 and S2 Figs and S1 Table). The DGRP population consisted 212 strains for which Illumina short-read sequences were available. We genotyped each sample using Illumina short-reads with IMR/DENOM [12], an algorithm we developed previously which combines iterative short-read mapping and *de novo* assembly for reliable variant calling and reassembly. By comparing with a long read (Pacific Biosciences) based *de novo* assembly of the *A*. *thaliana* accession L*er*-0 [16], we estimated that 3.1% of variants were incorrectly called and a further 2.3% of variants were mistakenly called as reference for accessible regions [15, 17] (S1 Text). We then compared IMR/DENOM’s calls around the well-characterised *FRI* complex of variants to the dideoxy sequences from 18 *A*. *thaliana* accessions. We confirmed that both a 16bp insertion and a 345bp complex deletion were correctly identified, including 31bp novel sequence within the complex deletion [12]. The high accuracy of our INDEL calls, allowed us to determine these complex variants accurately across large populations, and thus directly test them for association. The local densities of SNPs and INDELs correlated to each other (p-value < 2.2e-16 with Kendall rank correlation test; Fig 2A inner cycles and Fig 2B). And the INDEL/SNP ratio in CDS regions was significantly lower than that on the whole genome level (p-value < 2.2e-16, paired Wilcoxon signed rank test, Fig 2C).

**Fig 1:**
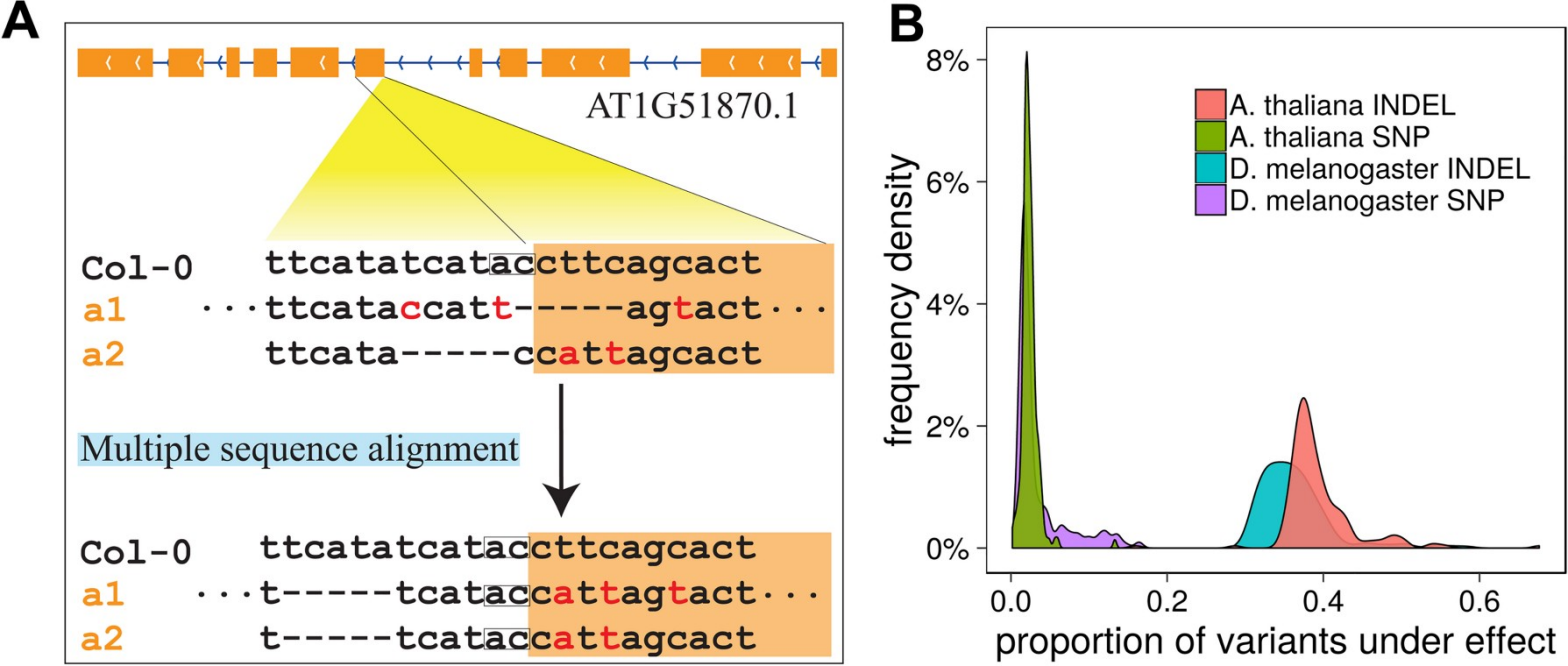
Inconsistent alignments contribute significantly to INDEL multi-allelic. (A) An example of the same sequence divergence encoded as different variants when the surrounding sequences are different between accessions. The deletion in the first alignment implicates an ORF shift with the encoded gene AT1G51870, while the deletion in the second alignment implicates an interruption of the splice motif. With multiple sequence alignment, the deletion is located in intron region. (B) Density plot of proportions of variants affected by multiple sequence alignment per sample.

When aligning a divergent sequence to a reference genome, alignment isomorphs frequently occur, where the essentially same sequence is aligned in different ways (Fig 1A). In the context of variant calling for the *A*. *thaliana* or *D*. *melanogaster* genomes under study, this ambiguity results in false multi-allelic calls for the same allele, even if the surrounding sequence is same between strains. In supporting of this, we observed that ~63.18% and ~64.49% of INDELs from the population (including insertions and deletions >1kbp, which are usually categorized as SVs) had more than one alignment isomorph among the 177 *A*. *thaliana* ecotypes and the 212 inbred lines of *D*. *melanogaster* respectively. We characterised common scenarios where false multi-allelic calls occur due to alignment isomorphism (Fig 1A and S3 Fig). These ambiguities would be expected to reduce the power of association testing since unnecessary degrees of freedom are used to estimate the effects of apparently distinct but—in reality—identical alleles.

To mitigate this problem, we developed software Irisas to synchronize INDEL and SNP variant calls. Irisas aligned haplotypes of samples using multiple sequence alignment (MSA) with overlapped windows, and re-called variants in a consistent and synchronized manner. We found that on average 39.43% of INDELs in *A*. *thaliana* and 36.30% in *D*. *melanogaster* per line were reassigned to a shared allele, compared to the original variants. In contrast, variant calling ambiguities affected only 2.40% SNPs in *A*. *thaliana*, and 3.53% in *D*. *melanogaster* per sample, the majority of those having at least one INDEL within 10bp (Fig 1B).

This analysis prompted us to create a robust burden test, in order to evaluate the joint effects of INDELs and SNPs in coding regions [18]. Traditional burden testing is based on combining the annotated effects of SNPs. Each SNP is classified (e.g. nonsynonymous, nonsense and benign) based on functional annotation of the reference genome. Our previous work [12] had shown that this approach overestimates the numbers of deleterious coding variants because gene models vary, particularly around splice sites. Irisas integrated all the variants (i.e. SNPs, INDELs and SVs) so that the functional impact of each variant was evaluated collectively and conservatively. It focused on variants or groups of variants which change open reading frames (by INDELs/SVs), destroy splice sites or cause premature stop codons (by INDELs/SVs or SNPs). We call these events “open reading frame state” (ORFS) changes hereafter. The existence of an ORFS change in a gene implies a change in protein sequence and possibly of its function. The results of our ORFS algorithm on 18 *A*. *thaliana* accessions were 98.9% concordant when compared to the previous annotation (S2 Table) which integrated RNA-seq and *ab initio* gene prediction [12].

### INDELs and ORFSs explain a large proportion of phenotypic variance not explained by SNPs

We performed GWAS for *A*. *thaliana* and *D*. *melanogaster* [13] using linear mixed models. To adjust for the population structure, two kinship matrices were constructed, one using SNPs only and the other using all variants. Since only trivial differences for the association tests were observed using either matrix, we present only the SNP-only kinship matrix-based results (S8 Fig). As phenotypes were measured on different subsets of lines and the numbers of variants used for association tests changed accordingly, we calculated genome-wide significance thresholds for each phenotype separately using ‎Bonferroni correction and by permutation tests, with 1000 permutations per phenotype (S1 Text). Thus, four thresholds were calculated for each phenotype: 1) a genome-wide significance threshold derived from ‎Bonferroni correction and a genome-wide significance threshold from permutation tests using SNPs only, which serve as the benchmark for comparison, 2) an integrated Bonferroni significance threshold and an integrated permutation threshold using all Irisas variant calls. We found the integrated Bonferroni thresholds were only slightly higher than the SNP-only Bonferroni thresholds in all phenotypes, as the total numbers of tests increased by only 11–13% in both species. Integrated permutation thresholds were also slightly higher than SNP-only permutation test thresholds. In the subsequent analyses, the integrated permutation thresholds were used unless expressly stated otherwise. The association results for *A*. *thaliana* and *D*. *melanogaster* are summarized in Table 1. Manhattan and quantile-quantile plots for those 34 phenotypes in *A*. *thaliana* and ith at least one QTL passing the permutation threshold are presented in S15–S69 Figs. For simplicity, the genome-wide significant loci detected with SNPs, INDELs and ORFSs are referred to as snpQTL, indelQTL and orfsQTL respectively hereafter.

**Table 1:** Summary of GWAS results with three types of genotypic variants.

	phenotypes with at least one significant variant detected	phenotypes with significant SNP detected	phenotypes with significant INDEL detected	phenotypes with significant ORFS detected	Mean of variance explained by SNP	Mean of extra variance explained by INDEL	Mean of extra variance explained by ORFS
*A*. *thaliana*	34	24	17	12	~26.09%	~4.53%	~6.40%
*D*. *melanogaster*	21	18	5	0	~18.52%	~2.60%	~0%

**Fig 2:**
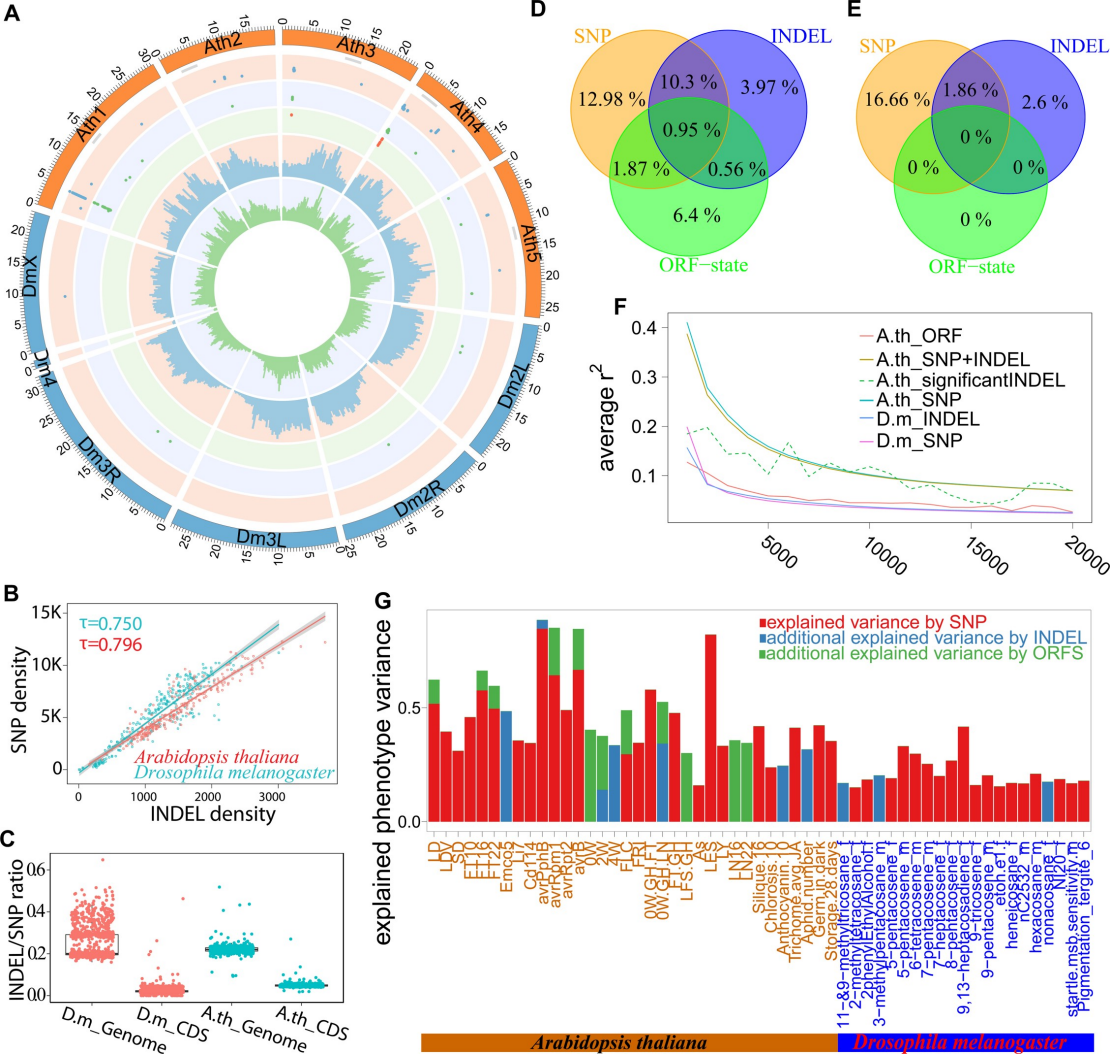
Association analysis of SNP, INDEL and ORFS in *Arabidopsis thaliana* (*A*. *th*) and *Drosophila melanogaster* (*D*. *m*). (A) Circos plot for association studies. From inner to outer: histogram plot of density for INDEL and SNP, dot plots of genome-wide significant loci for all the phenotypes by ORFSs, INDELs and SNPs respectively. (B) Correlation between INDEL density and SNP density. (**C**) SNP/INDEL ratios in CDS region and whole genome level. (**D, E**) Venn diagram comparing the variance explained by three types of genotypic variants for *A*. *thaliana* (**D**) and *D*. *melanogaster* (**E**). (**F**) LD decay patterns of different types of the variants, with SNP+INDEL for all INDELs and SNPs, *significantINDEL for the significant INDELs against their nearby SNPs and INDELs. (G) Phenotypic variance explained by genome-wide significant loci.

Fig 2A plots snpQTL, indelQTL and orfsQTL of all traits (Fig 2A outer 3 layers for snpQTL, indelQTL and orfsQTL of all traits respectively). Approximately 25.0% of snpQTLs are also genome-wide significant with INDELs or ORFSs (Fig 2D and 2E and S5A and S5B Fig). These included the association loci from 5 phenotypes (*AvrPphB*, *AvrRpm1*, *avrB*, *FRI*, *LES*) highlighted in the original publication [9] in *A*. *thaliana* and one phenotype in *D*. *melanogaster* (5-pentacosene concentration for male). All these loci showed very strong LD (S14–S19 and S49 Figs). No genome-wide significant association for long INDELs (SVs) was found, concordant with the low power of SVs for GWAS analysis observed in Mouse and Human studies [19, 20].

We suspected that loci identified only by SNPs or only by INDELs were due to incomplete LD. To this end, we analysed the LD patterns of SNPs and INDELs with MAF >10% and missing rate <50%. Our analysis was solely based on *A*. *thaliana* because the very rapid LD decay in *D*. *melanogaster* made it difficult to discern any effect. LD among SNPs in *A*. *thaliana* was on average stronger than LD among INDELs but the LD half-decay distances, where LD falls to half of its maximum value, were roughly the same (~2-3kbp). LD was stronger at loci where a cluster of significant INDELs or SNPs was detected. LD around isolated indelQTLs was usually weak (Fig 2F). This indicated that weak LD regions, such as the boundaries of haplotype blocks or at recombination hotspots, could be the cause of failure for genome-wide significant association for a particular phenotype in SNP-based GWAS.

We assessed the additional contribution of INDELs and ORFSs to the phenotypic variance. Since genotypic variants used for the association are not totally independent of each other, we estimated the effect size (i.e. variance explained), using a mixed model [21] with a SNP-based kinship matrix. We estimated the effect size of snpQTLs firstly and then added indelQTLs and orfsQTLs as additional independent variables (Fig 2G and Table 1). Compared to SNP-based association studies which explained on average 26.09% and 18.52% of variance for multiple traits in *A*. *thaliana* and *D*. *melanogaster*, INDELs and ORFSs explained an additional 10.93% and 2.60% phenotypic variance, respectively. Here we did not evaluate the heritability using a restricted maximum likelihood (REML) model suggested by GCTA [22]. The model showed inflated log-likelihood values for many phenotypes for SNP-based analysis, probably due to the small sample size we used [23, 24] or “synthetic” effects, which might be responsible for”ghost” associations in *A*. *thaliana* [25] and rice [26].

### Analysis of INDEL-specific and ORFS-specific genome-wide significant loci

**Fig 3:**
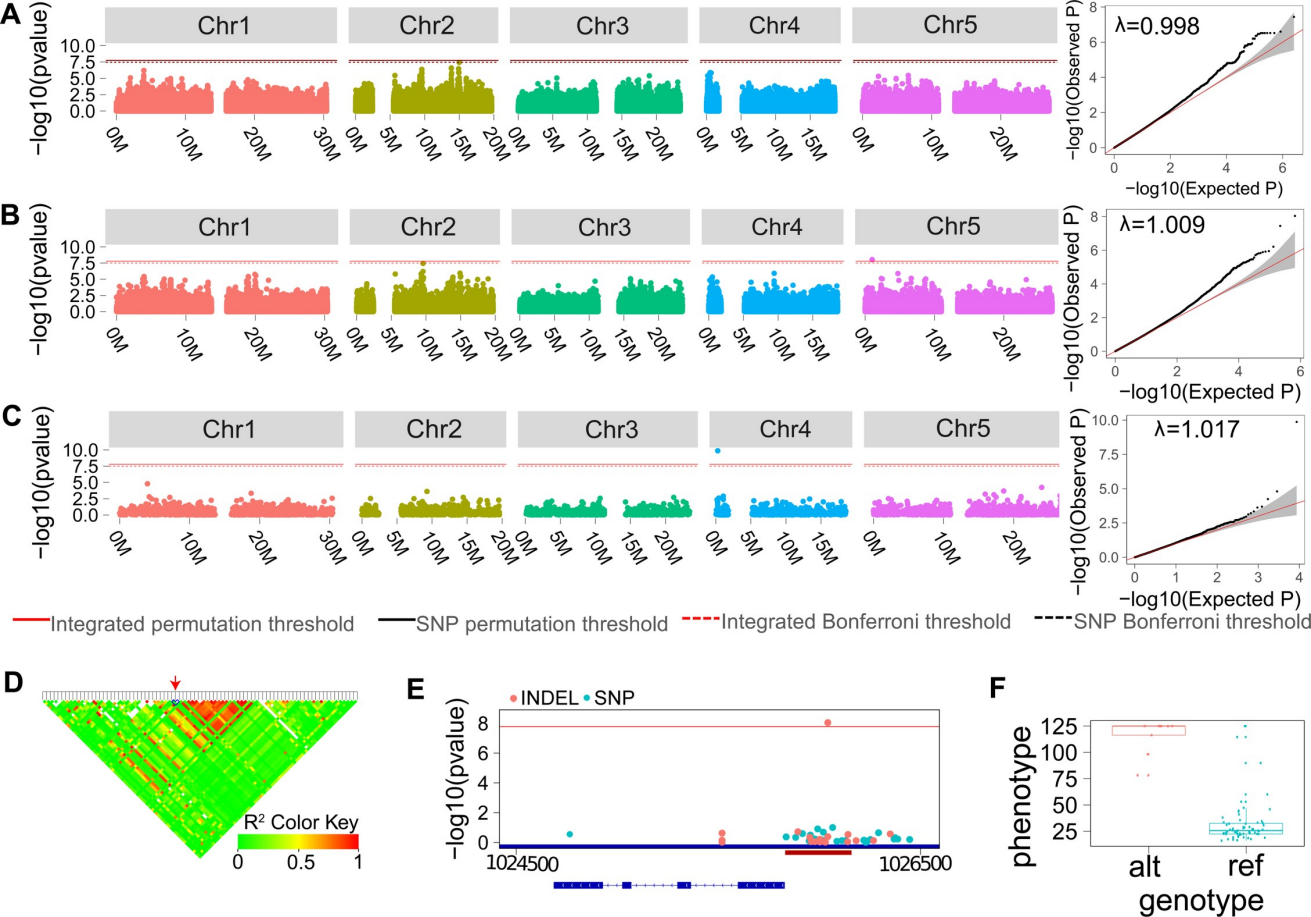
The INDEL and ORFS based association identified novel candidate loci. (**A**-**C**) The Manhattan and quantile-quantile (QQ) plot using SNPs (**A**), INDELs (**B**) and ORFSs (**C**) for phenotype “number of days required for the bolt height to reach 5cm with 2 weeks vernalization”. (**D**) LD heatmap for the genome-wide significant INDEL on chromosome 5, with the INDEL labelled in red. (**E**). Association plot for *TFL1* locus. The putative *TFL1* regulatory region is shown with the red bar. (**F**) Boxplot of the phenotype of different *A*. *thaliana* accession grouped by the allele at the significantly associated INDEL locus.

We next inspected certain INDEL-specific QTLs in more detail. For phenotype “days before the bolt reach 5cm” (S24 Fig), the highest scoring of which contains an isolated variant, a 1bp insertion on chromosome 5 (Fig 3A, B). The insertion, with allele frequency 0.125 (9 from 72 genotyped samples), shows very low average LD with nearby variants (Fig 3D). The most strongly linked SNP within 20kbp of the insertion is located 206bp upstream with *R*^2^ = 0.64 (*p*-value = 0.433 for the association test). The insertion is 249bp upstream of the start codon of *TERMINAL FLOWER 1* (*TFL1*), a region implicated in the regulation of the expression of *TFL1* [27–29] (Fig 3E and 3F, S6A Fig and S4 Table). Those accessions containing the insertion originate mainly from a small region in Sweden (S7 Fig).

In *D*. *melanogaster*, a 3 base-pair deletion was significantly associated with the concentration of 11- & 9-methyltricosane (11- & 9-Me-C23) in females (S51 Fig). These are pheromones in the epicuticular wax layer of abdominal tergites [30]. Only the role of 7-methyltricosane has been determined while those of 11- & 9-methyltricosane are unclear [31]. The associated deletion we identified is in the first intron or promoter region of *G-oα47A* that encodes G protein α o subunit (Gαo), which may affect pheromone signalling [32].

We next hypothesized that either collective sets of mutations, or loss of function via independent mutations on the same gene [33–36], might underlie orfsQTLs. We developed an integrated burden analysis to test this hypothesis. In *A*. *thaliana*, 2481 genes containing at least two distinct loss-of-function variants were identified using a graph theoretic algorithm (Materials and Methods), a large potential gene set for integrated burden testing. Among them is *FRI* with a 16bp insertion and a 345bp complex deletion both affecting the protein’s functionality [8, 37]. *FRI* regulates *FLOWERING LOCUS C* (*FLC*) and thereby flowering time in multiple plant species [8, 38]. Interestingly, *FRI* did not reach genome-wide significance in SNP-based GWAS for flowering time even when these two INDELs were genotyped and tested specifically [9, 39]. Since both variants were common (allele frequency 14.12% and 16.38% respectively within the population), the loss of power here was not due to many rare variants with small effects. The distribution of loss-of-function *FRI* alleles in the population showed no obvious pattern (S7 Fig). Further simulations confirmed that independent loss-of-function alleles pose a big challenge to GWAS (S1 Text) especially for small population samples, but have a higher power of detection by burden testing. In our study, *FRI* achieved genome-wide significance for many flowering time phenotypes (S20–S29 Figs).

### INDELs and ORFSs contribute to expression variation

Next, we investigated whether INDELs and ORFSs associate with gene expression variation [40, 41]. To this end, 628 *A*. *thaliana* natural accessions with RNA-seq from the 1001 Epigenomes Project [42] were chosen. The genomic variants were called using genomic sequence data from 1001 genome project and assembled with IMR/DENOM, and then synchronized and ORFS called using Irisas. Overall, ~3.87 Million SNPs, 1.96 Million INDELs and ORFSs of 14 thousand transcripts passed our quality checks for further analysis. We tested the association for the expression of 19,844 genes using linear mixed models. Among them, 10,508 genes have at least one eQTL reached integrated permutation test threshold (FDR< = 0.05).

**Fig 4:**
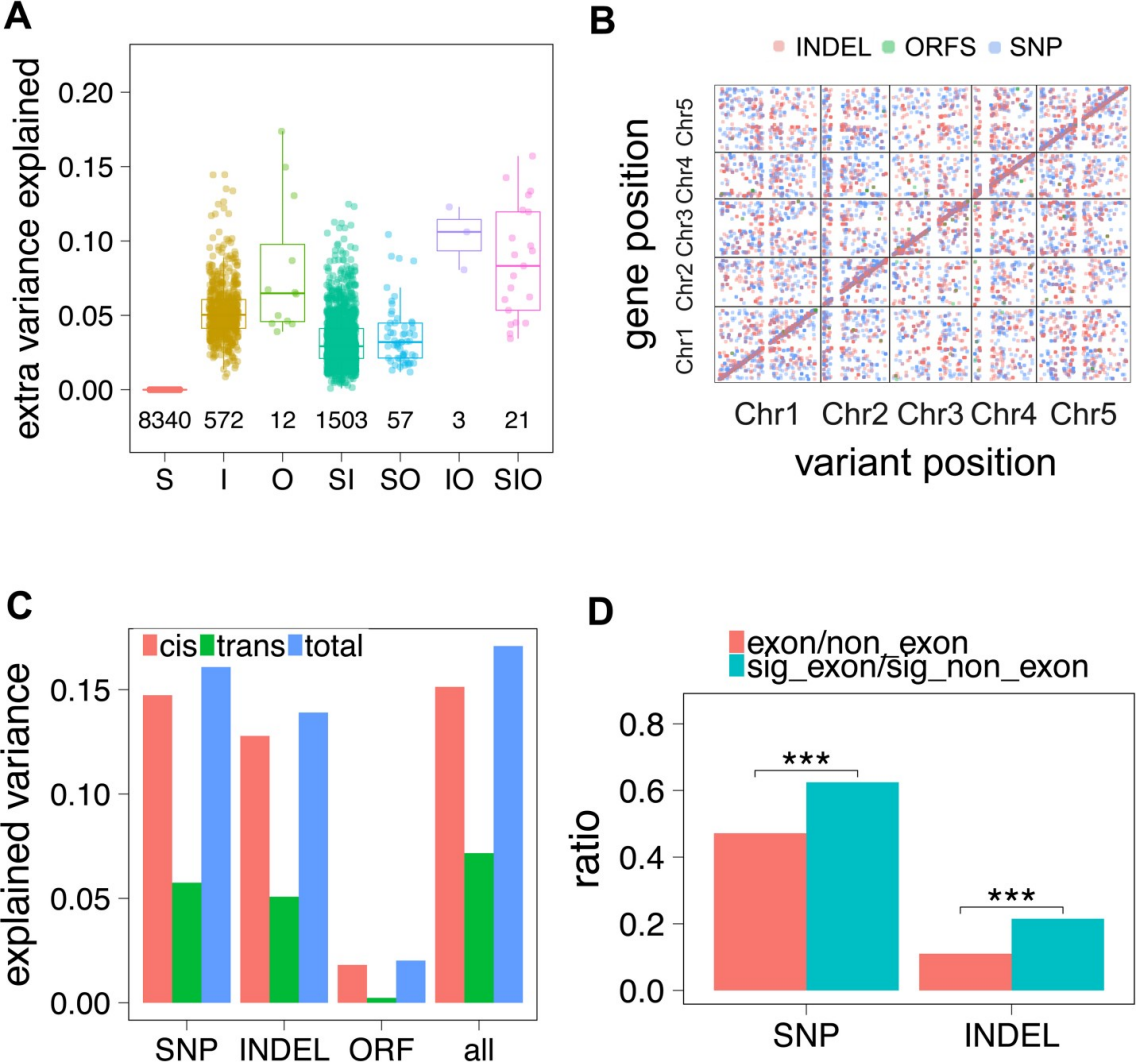
eQTL analysis based on INDELs and ORFSs. (A) Extra expression variance being explained by indelQTLs and orfsQTLs. S: Genome-wide significant snpQTLs were detected and no extra variance could be explained by either indelQTLs or orfsQTLs; I: No snpQTLs were detected, indelQTLs were detected and orfsQTLs could not explain extra variance; O: Neither snpQTLs nor indelQTLs were detected, while orfsQTLs were detected; SI: snpQTLs and indelQTLs were detected, and indelQTLs explained additional variance, no orfsQTL were detected or orfsQTLs could not explain additional variance; SO: snpQTLs and orfsQTLs were detected, and orfsQTLs explained additional variance, while no indelQTLs were detected or indelQTLs could not explain additional variance; IO: no snpQTLs were detected, indelQTLs and orfsQTLs were detected, and orfsQTLs explained additional variance; SIO: snpQTLs, indelQTLs and orfsQTLs were detected, and both indelQTLs and orfsQTLs explained extra variance. (**B**) The start position of the mapped genes was plotted against the chromosome position of the associated genotypic variants. (**C)** The proportion of explained expression variance. (D) The ratios of the number of snpQTLs and indelQTLs located in exon region to outside-exon region (cyan) against genome-wide (red) SNPs and INDELs.

Inspection of these eQTLs revealed that 16.28% of expression variance was explained by eQTLs from SNPs, and 13.90% from INDELs and 2.02% from ORFSs. A large portion of expression variance explanation was shared. INDELs and ORFSs explained 0.81% extra variance on average (Fig 4A, S9 Table, S11C–S11H Fig). This indicated the pronounced effect from the strong LD between SNPs and INDELs. SNPs, INDELs and ORFSs were all predominately associated as *cis*-eQTL (i.e. within 30kbp of the gene) (Fig 4B and 4C). Additionally, the majority of eQTLs were identified outside of coding regions (S11I Fig). The ratio of the number of eQTL inside exons to those outside is significantly higher than genome-wide levels for both SNPs and INDELs (Fisher's exact test, *p*-value<0.001) (Fig 4D), indicating that sequence polymorphisms inside a gene were also playing a role in gene expression regulation, possibly through nonsense-mediated decay [43] or sRNA pathways [44].

## Discussion

We devised the software Irisas to perform GWAS based on INDELs and ORFSs for a large number of phenotypes in both plant and animal models. We focused on sequenced inbred populations in both species in which variant-calling is more robust. Our results show that INDELs and burden testing using ORFSs are both capable of revealing associations with causal variants that would not have been detected by SNPs alone. There are two main reasons for this successful recovery of missing heritability. The first is that we removed multi-allelic artefacts caused by inconsistent alignment isoforms, as shown in the comparison of INDEL-association with or without proposed variant synchronization procedure (S1 Text, S13 Fig). The second stems from our novel ORFS calling procedure, which ameliorated the lack of power for independent test of multiple common alleles which cause loss-of-function of the same transcript and thus have same functional effect (S1 Text).

Testing for INDELs as well as SNPs increases the number of tests by only 11–13%. Since many INDELs are in LD with SNPs, the effective number of tests will increase modestly, resulting in only slightly higher thresholds for significance, and a correspondingly larger sample size is required to maintain power. This is generally accepted and the trend across association studies in all species is to increase sample sizes and to test more exotic variants including INDELs. An alternative, where additional samples are unavailable, would be to perform an integrated analysis of certain candidate regions that are marginally significant with SNPs at a certain threshold. This strategy could uncover the loci where SNPs are only partially linked with causal INDELs, with the possibility of losing the loci containing isolated causal INDELs.

Burden analysis is often regarded as a useful supplement to independent SNP association. While SNP burden tests have been under intensive investigation, the effect of INDELs has been largely overlooked. In our GWAS, ORFSs explained a significant additional fraction of phenotypic diversity either by identifying novel loci or by increasing the power of detected loci. Notably, our tests also revealed that the loss-of-function alleles in the reference genome could seriously affect the ORFS-based association analysis. For example, in *FRI*, where the reference genome Col-0 contains a non-functional version of the gene (S10 Table), the locus cannot achieve genome-wide significance unless the functional version of the gene is used to determine ORFSs. This indicates that expert knowledge of gene functionality is important for burden testing. We anticipate that more advanced burden analyses will further help understand the contribution of INDELs to phenotypic diversity.

## Materials and methods

### Datasets used

(a) We chose a set of *Arabidopsis thaliana* accessions whose phenotypes had been investigated previously [9] and whose genomes have been sequenced through the 1001 genomes project [15]. In depth, we checked the overlap of those two datasets by comparing the ecotype ID. For those lines whose phenotypes have been measured but the genome sequences are not available in the 1001 genomes project, we used the genome sequence from the plants with the same accession name if the SNPs identified from SNP-array are consistent with the Illumina sequence data. The final list of samples contains 177 accessions (S1 Table), from worldwide regions (S1 Fig). The public “TAIR10” genome [45] were used as reference. (b) For expression quantitative trait loci (eQTL) experiment, the leave transcriptomes of 728 accessions were obtained from the public database [42].

The *Drosophila* Genetic Reference Panel (DGRP) consist 212 inbred *D*. *melanogaster* (fruit fly) lines with whole genome sequenced using Illumina shotgun sequencing. A wide range of phenotypes have been measured and released for subsets of those lines. The FB6.11 genome sequence was used as reference.

### Genomic variant calling with IMR-DENOM

The variant calling was performed with IMR-DENOM v 0.5, which integrates iterative reads mapping and *de novo* variant calling [12]. Different *k*-mers were chosen for the *de novo* assembly part by setting parameter “-k” based on the read length and coverage. For *A*. *thaliana*, the default parameter was used when read length smaller than 75; “-k 65” when read length larger than 100 and coverage larger than 30X; “-k 45” otherwise. For *Drosophila melanogaster*, “-k 45” was used.

### Integrated region-based variant synchronization

As demonstrated in Fig 1 and S3 Fig, the same underlying clustered variants could be presented as inconsistent alignments. To synchronize variants, we used multiple sequence alignment (MSA). However, the computational complexity of multiple sequence alignment increases exponentially with the sequence length and number of samples. It is difficult, if not impossible, to directly apply MSA to a whole chromosome in a large population study.

To alleviate the computational load of MSA, we here proposed a sliding window based scheme. In details, our algorithm consists of three steps: 1) the reference genome was split into windows of 50,000 bases overlapping by 1,000 bases (parameters can be tuned in Irisas). Within each window, each haplotype sequence was assembled with its variants; 2) perform multiple sequence alignment for all haplotypes and the reference genome, and call variants for each haplotype based on the MSA. Irisas used MAFFT v7.213 [46] (parameter—auto) by default for the MSA, though other MSA software is also supported; 3) resolve the conflict within the overlapped regions and integrate the variants. Below we explain step 3 in details.

We called the variants from MSA base-pair by base-pair, for example, a 10 base-pair deletion was treated as 10 atomic one base-pair deletions. This allows us to resolve the conflict within the overlapped regions easily based on the coordinate. When two atomic variants within overlapped regions in the two consecutive windows were in conflicts, those closer to the center of their window were chosen. The atomic variants were then linked together. For example, two 1bp deletions at adjacent positions were merged as a single 2bp deletion. Overlapping INDELs were treated independently as two variants.

### Genes’ ORF state annotation for association studies

As described previously in our publication [12], alternative gene models often restore protein-coding in a natural accession. In addition, insertions and deletions can be called in different forms at different genomic positions, as shown in Fig 1A. Evaluating a gene’s ORFS based on each mutation independently would cause serious problems. The *de novo* annotation method suggested in the previous study [12] was computationally intensive and required the additional transcriptome sequence data, which could be unavailable in certain cases. As re-annotation indicated that the conservation of the protein coding had a dominate effect [12], we thus implemented a light-weight algorithm in Irisas to detect the change of ORFS in a gene. The algorithm includes three steps: (a) obtaining the assembled haplotype of the gene; (b) aligning both the CDS and the protein sequences of the reference to the haplotype sequence and annotating the gene structure accordingly; (c) merging the two gene models from step b and the direct lift over result, and the most conserved version was chosen for the ORFS. The details are as follows:

- CDS based orthologous annotation
  The genome sequences of each gene from each accession were extracted from the assembled sequence including 1kbp upstream and 1kbp downstream regions. The CDS sequences of reference annotation were mapped to the genome sequence using exonerate (V2.2.0) with parameters “—maxintron 30000—model est2genome -i -10—score 10—bestn 1—minintron 10” (intron length not less than 10 and not more than 30000, penalty -10 for introduction of an intron, and only report the best result with minimum score 10).
- Protein based orthologous annotation
  The protein sequences of TAIR10 were mapped to haplotype sequences using exonerate with parameters: “—bestn 1—maxintron 30000—intronpenalty -10—model protein2genome—percent 10—score 10—minintron 10”.
- Final annotations for the ORFS
  The gene annotations generated with the above two methods and the annotation from lift over were checked with the following criterions:
  1. Are the start codon and the stop codon intact?
  2. Has any of the splicing sites been interrupted? A splicing site is regarded as intact if its sequences are same with reference sequences, or follow the GT-AG rule [47], or belong to one of combinations of GC-AG, GG-AG, GT-TG, GT-CG, CT-AG.
  3. Is there any premature stop codon?
  4. Is the full length of CDS (protein coding sequences) sequence of the gene is dividable by 3?

A gene’s ORFS was regarded as interrupted if each of its three different annotations had satisfied at least one of criterions, otherwise it will be regarded as ORFS-conserved.

For any gene whose haplotype in the reference genome is not functional, a working version from other natural accession was chosen. For example, *FRI* in the reference genome Col-0 is not functional, we chose accession Eden-1 (ecotype id 6009) as reference.

### Genomic variants encoding and filtering

SNPs that were biallelic within the population were used for association. A SNP was treated as missing if: (1) The number of reads supporting it is less than 2; (2) heterozygous; 3) physically overlapped by an INDEL.

INDELs from different accessions could occur at the same position or overlap with each other (S12C Fig). Here only INDELs/SVs with the same position and same length were encoded as the same variant. An INDEL would be genotyped as missing in an accession if a different INDEL were found to occupy a part of its positions in that accession. There were totally 3820962 INDELs in *A*. *thaliana* and 2290193 INDELs in *D*. *melanogaster* encoded.

### Test for the existence of homogeneous lines

Homogeneous lines could affect the power of association analysis. To filter out homogeneous lines, the identity by state (IBS) matrix was constructed for the population using PLINK [48] (v 1.9) with SNPs. In *A*. *thaliana*, SNPs passing the following procedures would be used to construct IBS matrix:

1. SNPs located within centromere regions were removed as the recombination is rare and the variant calling is less reliable in these regions due to a large proportion of duplications;
2. SNPs with minor allele frequency (MAF) less than 5% or missing rate higher than 10% were dropped;
3. The nearby highly linked SNPs were pruned with function:—indep-pairwise 2000 1000 0.9 (window size 2000bp, windows overlapping size 1000bp pairwise SNPs with r^2^>0.9 with only one being kept).

For *D*. *melanogaster*, we used the following procedures:

1. SNPs within 2L:0.4Mb-14.9Mb, 2R:9Mb-18Mb and 3R:6Mb-27Mb were excluded since major inversions
2. SNPs with MAF less than 5% or missing rate higher than 20% were dropped
3. The nearby highly linked SNPs were pruned with function:—indep-pairwise 2000 1000 0.9

In *A*. *thaliana*, for the group with pair-wised IBS > 0.9, we ranked each sample firstly with the ecotype ID identification and then sequencing quality. That is, in a homogeneous group, if two accession shares the same accession name but with different ecotype ID, it will be removed preferentially. We ranked *D*. *melanogaster* with only sequencing quality. The accession with the highest rank in a homogeneous group would be kept for the following GWAS analysis.

We used the number of trustable ORFSs as an indicator of the sequencing quality of each sample. Accessions sharing a high IBS index are expected to share very a similar number of trustable ORFSs. An ORFS was treated as trustable if it followed the following criteria:

1. Coverage of every base pair of the CDS region equal to or large than 1
2. All the INDEL physically falling into the CDS region could be confirmed by both *de novo* assembly and reference guided assembly

More reliable ORFSs were used as an indication of higher sequencing quality.

There are 207 *D*. *melanogaster* lines left for GWAS analysis. Since different sets of *A*. *thaliana* were used in different phenotyping experiments, filtering was performed for each phenotype separately with the same IBS matrix. Each variant was filtered with the phenotype specific accessions list from the original full variants dataset.

### GWAS analysis

For *A*. *thaliana*, the phenotypic values were transformed according to the original report [9]. The genotypic variants were filtered with MAF (> = 0.1, as Atwell et al.[9]), missing rate (< = 0.5), minor allele number (> = 5). Those inside centromere regions were also exempted from the subsequent association tests. We used EMMAX pipeline for association tests. To correct population structure, the kinship matrixes were generated with the EMMAX-BN (Balding-Nichols) method. A modified version of EMMAX was used when analysing INDELs. We excluded an accession if it were genotyped as missing.

In *D*. *melanogaster*, the genotypic variants were filtered with MAF > = 0.05, missing rate < = 0.2 and minor allele number > = 8 before being used for association analysis. To estimate kinship matrix, the SNP dataset was further filtered with LD pattern (–indep-pairwise 1000 500 0.2 in PLINK) and SNPs within 2L:0.4Mb-14.9Mb, 2R:9Mb-18Mb and 3R:6Mb-27Mb were excluded since major inversions. The phenotypes were adjusted for the effects of *Wolbachia* infection and major inversions (In(2L)t, In(2R)NS, In(3R)P, In(3R)K, and In(3R)Mo) according to the DGRP2 paper [13]. The adjusted values were then transformed with the WarpedLMM [49] package. We trained WarpedLMM (with default settings) using the same SNP datasets used for kinship matrix construction. The transformed phenotype values were fitted into association algorithm FAST-LMM (v 2.0) [50]. The Manhattan and QQ plots were visualized with homemade R [51] scripts.

### Proportion of phenotypic variance explained by significant genotypic variants

To assess how much of the phenotypic variance can be explained by detected genome-wide significant loci, we estimated the effect size of those variants with regression analysis.

Suppose that *n* measurements of a phenotype were collected across *t* inbred strains. A linear mixed model in model organism association mapping is typically expressed as

y=μ+xβ+Zu+e (1)

where *y* is an *n* × 1 vector of observed phenotypes, and *μ* is the intercept. x is an *n* × *q* matrix of fixed effects. β is a 1 × *q* vector representing coefficients of the fixed effects. Z is an *n* × *t* incidence matrix mapping each observed phenotype to one of *t* inbred strains. In this study Z is always an identical matrix and could be ignored. *u* is the random effect of the mixed model with Var(*u*) = $\sigma_{g}^{2}K$. K is the *t* × *t* kinship matrix and here *t* = *n*. And *e* is an *n* × *n* matrix of residual effect such that Var(e) = $\sigma_{e}^{2}I$.

Let S be the significant SNPs as fixed variables (Eq 3).

$${y=\mu }_{s}{+S\beta }_{s}{+\mathrm{Zu}+e}_{S}$$ (2)

$$\hat{y_{s}}=\mu_{s}{+S\beta }_{s}$$ (3)

where β_s_ is the coefficients of the significant SNPs and can be estimated with the mixed model.

The proportion of phenotypic variance explained by significant SNPs, denoted by $h_{S}^{2}$, was estimated as (Eq 4)

$$h_{S}^{2}=1‑\Sigma (y-{\hat{y_{s}})}^{2}/\Sigma (y-\bar{y}{)}^{2}$$ (4)

where $\bar{y}$ is the mean phenotype value of all the accessions. The contributions of INDELs were then added and the corresponding additionally explained phenotypic variances were estimated as:

$${y=\mu }_{s+i}{+S\beta }_{s}{+I\beta }_{i}{+\mathrm{Zu}+e}_{S+I}$$ (5)

$$\hat{y_{s+I}}=\mu_{s+i}+S\beta_{s}+I\beta_{i}$$ (6)

$$h_{S+I}^{2}=1‑\Sigma (y-\hat{y_{s+I}}{)}^{2}/\Sigma ({y-\bar{y})}^{2}$$ (7)

$$h_{I}^{2}=h_{S+I}^{2}‑h_{s}^{2}$$ (8)

where I is the contributions of INDELs, $h_{S+I}^{2}$ is the variance explained by significant SNPs and INDELs together and $h_{I}^{2}$ is the additional phenotypic variance explained by significant INDELs.

We then evaluated the contribution from ORFSs with

$$y=\mu_{s+i+o}+S\beta_{s}+I\beta_{i}+O\beta_{o}+Zu+e_{S+I+O}$$ (9)

$$\hat{y_{s+I+O}}=\mu_{s+i+o}+S\beta_{s}+I\beta_{i}+O\beta_{o}$$ (10)

$$h_{S+I+O}^{2}=1‑\Sigma (y-{\hat{y_{s+I+O}})}^{2}/\Sigma (y-\bar{y}{)}^{2}$$ (11)

$$h_{O}^{2}=h_{S+I+O}^{2}‑h_{S+I}^{2}$$ (12)

where O is the contributions from ORFSs, $h_{O}^{2}$ is the additional phenotypic variance explained by orfsQTLs.

Since some significant genotypic variants are highly correlated with each other, when a variant is added, we performed marginal association analysis. Only those variants that could explain significant proportion of variance (*p*-value<1e-4 with *F*-test performed on residual sum of squares, this method is expected to outperform extended Bayesian information criterion [21]) would be kept as a covariant for the subsequent analysis.

The above analysis was performed within the statistical frame work of EMMA [52] using Python version of MLMM [21]. The (additional) variances explained by a specific type of variants were reported by averaging multiple association studies where at least one QTL detected.

### Detection of independent ORFS-shift mutations

We used a graph theoretic model to detect the independent ORFS-shift events. Each ORFS-shifting transcript was regarded as a node. An edge would be created between two nodes if they have variants overlap with each other in genomic position (S9A Fig). If all ORFS-shift transcripts shared the same ancestry, every node in the graph would be linked with each other and a complete graph would be formed (S9B Fig).

We detected the independent ORFS-shift transcripts using all sequences released from 1001 genomes project with the following criteria:

1. Only trustable transcripts were used.
2. The ORFSs were shifted in more than 130 accessions (analogous to the MAF of ORFS).
3. The graph constructed is at least one edge from being complete.

The GO enrichment analysis was performed using agriGO [53] V1.2 with default parameters.

## Supporting information

S1 Text Supporting results and methods. (PDF)

S1 Fig The geographical distribution of *A*. *thaliana* accessions whose phenotypes were measured in the original publication. Those in green are accessions whose Illumina shotgun sequence data are unavailable, and thus excluded from association analysis in this study.
(PDF)

S2 Fig The identical by state (IBS) matrix of the *A*. *thaliana* accessions used in this study. Accessions were indicated with ecotype ID and those accessions colored black had no very similar accessions detected. And those similar accessions were labeled with same color.
(PDF)

S3 Fig Several typical scenarios where the false multiallelic calls occur due to inconsistent alignment isomorph. (a) An INDEL can be placed at multiple positions and could be unified with available left alignment algorithm. (b) A haplotype could be represented with different type of variants. (c) A haplotype could be represented with several different INDEL/SNP combinations.
(PDF)

S4 Fig Normalized INDEL and SNP distribution. (a, b) SNPs and INDELs distribution of *A*. *thaliana*. (c, d) SNPs and INDELs distribution of *D*. *melanogaster*. Relatively less INDELs and SNPs have been observed in CDS regions comparing with other genomic regions.
(PDF)

S5 Fig Venn diagram summary and LD pattern of detected QTLs. (a) The venn diagram for phenotypes of *A*. *thaliana* with different QTLs using three genotypes. (b) The venn diagram for phenotypes of *D*. *melanogaster* with different QTLs using three genotypes. (c) LD decay patterns of different types of *D*. *melanogaster* INDELs, *significantINDEL for the significant INDELs against their nearby SNPs and INDELs.
(PDF)

S6 Fig Phenotype of *tfl1* and *svp*. (a) The functional validation of TFL1 locus by comparing two *tfl1* T-DNA mutation lines to the wild-type Col-0 accession. (b) The functional validation of SVP locus by comparing *svp* T-DNA mutation lines to the wild-type Col-0 accession. (16 days old plants grown under the condition specified in the original association study).
(PDF)

S7 Fig Geographical and sub-population distribution of FRI and TFL1 natural alleles. (a) The geographical distribution of predicated functional and loss-of-function FRI alleles. (b) The sub-population distribution of predicated functional and loss-of-function FRI alleles. (c) The geographical distribution of TFL1 nearby significant INDEL. (d) The sub-population distribution of TFL1 nearby significant INDEL.
(PDF)

S8 Fig GWAS analysis when using kinship matrix constructed from combined variants. (**a**-**c**) The Manhattan and quantile-quantile (QQ) plot using SNPs (**a**), INDELs (**b**) and ORFSs (**c**) for phenotype “number of days required for the bolt height to reach 5cm with 2 weeks vernalization” when using kinship matrix constructed from combined variants of SNP and INDEL. (**d-f**) Comparing the -log10(pvalue) of SNP (**d**), INDEL (**e**), ORFS (**f**) using kinship matrix constructed from SNP variants versus combined variants.
(PDF)

S9 Fig The analysis of independence of ORF-shift variants. (a) A cartoon shows how to infer the independence of ORF-shift variants from sequence diversity. (b) An example of dependent ORF-shift variants. (c) Examples of two independent ORF-shift variants.
(PDF)

S10 Fig GO terms that are enriched (dark blue) in the set of 2481 genes containing independent ORF-shift variants and their parental terms (light blue). (PDF)

S11 Fig eQTL analysis of an *A*. *thaliana* population with 728 accessions. (a) Principal component analysis (PCA) of the expression matrix. The expression values were log10 transformed before analysis. (b) PCA of the expression matrix from 628 accessions after removing outliers and homogeneous individuals. (c) The start position of the mapped genes was plotted against the chromosome position of the associated SNPs. (d) The start position of the mapped genes was plotted against the chromosome position of the associated INDELs. (e) The start position of the mapped genes was plotted against the chromosome position of the associated ORFSs. (f) The variance being explained by *cis*-eQTLs for the expression level of *A*. *thaliana* genes with eQTL detected. (g) The variance being explained by *trans*-eQTLs for the expression level of *A*. *thaliana* genes with eQTL detected. (h) The variance being explained by all eQTLs for the expression level of *A*. *thaliana* genes with eQTL detected. (i) The count of snpQTLs and indelQTLs located in exon region against those of SNPs and INDELs outside exon region.
(PDF)

S12 Fig Simulation of the effect of common independent loss-of-function causal variants. (a) A cartoon indicates an independent loss-of-function gene, with box represent CDS sequence. The allele 1 is a functional allele. Allele 2 and allele 3 indicate loss-of-function allele due to ORF of them were shifted by INDEL B and INDEL C independently. (b) Phenotypes of allele 1 were simulated with mean 2, variance 1.44 and 70 samples. Phenotypes of allele 2 and allele 3 were simulated with mean value 1, variance 1.44 and 70 samples. The association based on present/absent of single INDEL and ORF states were performed with Wilcoxon rank sum test. This process was repeated for 10, 000 times, and the corresponding p-values were illustrated with a violin plot. The value of red line is 7.8, which is set as whole genome level significant threshold. (c) A cartoon indicates physically overlapped deletions.
(PDF)

S13 Fig The comparison of the effect size of detected QTLs between associations with and without variants synchronization by Irisas. (a) The phenotype variance being explained by SNPs and additional variance explained by INDELs without variants synchronization. (b) The phenotype variance being explained by SNPs and extra variance explained by INDELs after variants synchronization. (c, d) The plot of the ratios of phenotypic variance explained by SNPs and INDELs in GWAS analyses with and without variants synchronization in *A*. *thaliana* (c) and *D*. *melanogaster* (d).
(PDF)

S14 Fig Summary of GWAS results for Sodium concentration (Na) (*Arabidopsis thaliana*). (PDF)

S15 Fig Summary of GWAS results for *AvrPphB* (*Arabidopsis thaliana*). (PDF)

S16 Fig Summary of GWAS results for *AvrRpm1* (*Arabidopsis thaliana*). (PDF)

S17 Fig Summary of GWAS results for *AvrB* (*Arabidopsis thaliana*). (PDF)

S18 Fig Summary of GWAS results for FRI gene expression (FRI) (*Arabidopsis thaliana*). (PDF)

S19 Fig Summary of GWAS results for leaf presence or absence of lesioning (LES) (*Arabidopsis thaliana*). (PDF)

S20 Fig Summary of GWAS results for Days to Flowering under Long Days (LD) (*Arabidopsis thaliana*). (PDF)

S21 Fig Summary of GWAS results for Days to Flowering at 16°C (FT16) (*Arabidopsis thaliana*). (PDF)

S22 Fig Summary of GWAS results for Days to Flowering at 22°C (FT22) (*Arabidopsis thaliana*). (PDF)

S23 Fig Summary of GWAS results for No vernalization, grown as JIC (0W) (*Arabidopsis thaliana*). (PDF)

S24 Fig Summary of GWAS results for 2 weeks vernalization, grown as JIC (2W) (*Arabidopsis thaliana*). (PDF)

S25 Fig Summary of GWAS results for FLC gene expression (FLC) (*Arabidopsis thaliana*). (PDF)

S26 Fig Summary of GWAS results for Length tile flower senescence, greenhouse (LFS GH) (*Arabidopsis thaliana*). (PDF)

S27 Fig Summary of GWAS results for leaf number 16°C (*Arabidopsis thaliana*). (PDF)

S28 Fig Summary of GWAS results for leaf number at 22°C (*Arabidopsis thaliana*). (PDF)

S29 Fig Summary of GWAS results for 0WGH LN (*Arabidopsis thaliana*). (PDF)

S30 Fig Summary of GWAS results for Days to Flowering under Short Days (SD) (*Arabidopsis thaliana*). (PDF)

S31 Fig Summary of GWAS results for Days to Flowering at 10°C (FT10) (*Arabidopsis thaliana*). (PDF)

S32 Fig Summary of GWAS results for 4 weeks vernalization, grown as JIC (4W) (*Arabidopsis thaliana*). (PDF)

S33 Fig Summary of GWAS results for Days to flowering, no vernalization, greenhouse (0WGH FT) (*Arabidopsis thaliana*). (PDF)

S34 Fig Summary of GWAS results for Days to flowering greenhouse (FT GH) (*Arabidopsis thaliana*). (PDF)

S35 Fig Summary of GWAS results for Days to Flowering under Long Days with vernalization (LDV) (*Arabidopsis thaliana*). (PDF)

S36 Fig Summary of GWAS results for Emco5 (*Arabidopsis thaliana*). (PDF)

S37 Fig Summary of GWAS results for Lithium concentration (Li) (*Arabidopsis thaliana*). (PDF)

S38 Fig Summary of GWAS results for AvrRpt2 (*Arabidopsis thaliana*). (PDF)

S39 Fig Summary of GWAS results for AS (*Arabidopsis thaliana*). (PDF)

S40 Fig Summary of GWAS results for presence or absence of either lesioning or yellowing (*Arabidopsis thaliana*). (PDF)

S41 Fig Summary of GWAS results for Silique length at 16°C (*Arabidopsis thaliana*). (PDF)

S42 Fig Summary of GWAS results for presence or absence of Chlorosis at 10°C (*Arabidopsis thaliana*). (PDF)

S43 Fig Summary of GWAS results for presence or absence of Anthocyanin10°C (*Arabidopsis thaliana*). (PDF)

S44 Fig Summary of GWAS results for Aphid number (*Arabidopsis thaliana*). (PDF)

S45 Fig Summary of GWAS result for Germination in the dark (*Arabidopsis thaliana*). (PDF)

S46 Fig Summary of GWAS result for Primary Dormancy with 28 days dry storage (Storage 28 days) (*Arabidopsis thaliana*). (PDF)

S47 Fig Summary of GWAS result for Cd114 (*Arabidopsis thaliana*). (PDF)

S48 Fig Summary of GWAS result for Trichome avg JA (*Arabidopsis thaliana*). (PDF)

S49 Fig Summary of GWAS result for 5-pentacosene_male (*Drosophila melanogaster*). (PDF)

S50 Fig Summary of GWAS result for 7-pentacosene_male (*Drosophila melanogaster*). (PDF)

S51 Fig Summary of GWAS result for 11-&9-methyltricosane_female (*Drosophila melanogaster*). (PDF)

S52 Fig Summary of GWAS result for 2-methyltetracosane_female (*Drosophila melanogaster*). (PDF)

S53 Fig Summary of GWAS result for Olfactory_behavior[2-phenyl_ethyl_alcohol]_female (*Drosophila melanogaster*). (PDF)

S54 Fig Summary of GWAS result for 3-methylpentacosane_male (*Drosophila melanogaster*). (PDF)

S55 Fig Summary of GWAS result for 5-pentacosene_female (*Drosophila melanogaster*). (PDF)

S56 Fig Summary of GWAS result for 6-tetracosene_male (*Drosophila melanogaster*). (PDF)

S57 Fig Summary of GWAS result for 7-heptacosene_female (*Drosophila melanogaster*). (PDF)

S58 Fig Summary of GWAS result for 8-pentacosene_tobepicture_female (*Drosophila melanogaster*). (PDF)

S59 Fig Summary of GWAS result for 9,13-heptacosadiene_female (*Drosophila melanogaster*). (PDF)

S60 Fig Summary of GWAS result for 9-tricosene_female (*Drosophila melanogaster*). (PDF)

S61 Fig Summary of GWAS result for 9-pentacosene_male (*Drosophila melanogaster*). (PDF)

S62 Fig Summary of GWAS result for Alcohol_sensitivity_[E1]_female (*Drosophila melanogaster*). (PDF)

S63 Fig Summary of GWAS result for heneicosane_female (*Drosophila melanogaster*). (PDF)

S64 Fig Summary of GWAS result for hexacosane_male (*Drosophila melanogaster*). (PDF)

S65 Fig Summary of GWAS result for nonacosane_female (*Drosophila melanogaster*). (PDF)

S66 Fig Summary of GWAS result for NI20_female (*Drosophila melanogaster*). (PDF)

S67 Fig Summary of GWAS result for Statle_oxidative_stress_male (*Drosophila melanogaster*). (PDF)

S68 Fig Summary of GWAS result for Pigmentation_tergite_6 (*Drosophila melanogaster*). (PDF)

S69 Fig Summary of GWAS result for nC2532_male (*Drosophila melanogaster*). (PDF)

S1 Table *A*. *thaliana* accessions used in the GWAS. Those in bold font are accessions whose sequence data are from an individual with same native name.
(DOC)

S2 Table Comparison between our ORFS algorithm and Gan *et al*.’s results with the progenitor accessions of *A*. *thaliana*. (DOC)

S3 Table Number of variants for each genotypic category used for association analysis. (DOC)

S4 Table The 5cm bolting height time and other flowering time related phenotypes of two *tfl*1 mutation lines under long day at 21°C in climate chamber. Seed sowed under long day, at 21°C. The plants were moved to short day, 4°C for vernalization after 5 days, then were moved back to long day, 21°C after 14 days.
(DOC)

S5 Table The flowering time related phenotypes of *svp* T-DNA mutation lines under long day condition at 22°C in climate chamber. (DOC)

S6 Table The flowering time related phenotypes of *svp* T-DNA mutation lines under long day condition in green house with natural light supplemented with artificial light. (DOC)

S7 Table The flowering time related phenotypes of *svp* T-DNA mutation lines under long day at 21°C in climate chamber. (DOC)

S8 Table The flowering time related phenotypes of *svp* T-DNA mutation lines under long day condition in climate chamber, with temperature at 20°C in daytime and 18°C at night. (DOC)

S9 Table Summary of eQTLs detected and expression level variance explained on average. (DOC)

S10 Table ORF-state predication of *FRI* in different accessions. (DOC)

## References

[1] Gudbjartsson DF, Walters GB, Thorleifsson G, Stefansson H, Halldorsson BV, Zusmanovich P, et al Many sequence variants affecting diversity of adult human height. Nature Genetics. 2008;40:609–15. 10.1038/ng.122 18391951

[2] Consortium tDGRAM-aD. Large-scale association analysis provides insights into the genetic architecture and pathophysiology of type 2 diabetes. Nature Genetics. 2012;44:981–90. 10.1038/ng.2383 22885922 PMC3442244

[3] van Rheenen W, Shatunov A, Dekker AM, McLaughlin RL, Diekstra FP, Pulit SL, et al Genome-wide association analyses identify new risk variants and the genetic architecture of amyotrophic lateral sclerosis. Nature Genetics. 2016;48:1043–8. 10.1038/ng.3622 27455348 PMC5556360

[4] Gibson G. Rare and common variants: twenty arguments. Nature Reviews Genetics. 2012;13:135–45. 10.1038/nrg3118 22251874 PMC4408201

[5] Gymrek M, Willems T, Guilmatre A, Zeng H, Markus B, Georgiev S, et al Abundant contribution of short tandem repeats to gene expression variation in humans. Nat Genet. 2016;48(1):22–9. 10.1038/ng.3461 ; PubMed Central PMCID: PMCPMC4909355. 26642241 PMC4909355

[6] Montgomery SB, Goode DL, Kvikstad E, Albers CA, Zhang ZD, Mu XJ, et al The origin, evolution, and functional impact of short insertion-deletion variants identified in 179 human genomes. Genome Res. 2013;23(5):749–61. 10.1101/gr.148718.112 ; PubMed Central PMCID: PMCPMC3638132. 23478400 PMC3638132

[7] Liu X, Geng X, Zhang H, Shen H, Yang W. Association and Genetic Identification of Loci for Four Fruit Traits in Tomato Using InDel Markers. Front Plant Sci. 2017;8:1269 10.3389/fpls.2017.01269 ; PubMed Central PMCID: PMCPMC5515879. 28769968 PMC5515879

[8] Johanson U, West J, Lister C, Michaels S, Amasino R, Dean C. Molecular Analysis of FRIGIDA, a Major Determinant of Natural Variation in Arabidopsis Flowering Time. Science. 2000;290:344–7. 10.1126/science.290.5490.344 11030654

[9] Atwell S, Huang YS, Vilhjálmsson BJ, Willems G, Horton M, Li Y, et al Genome-wide association study of 107 phenotypes in Arabidopsis thaliana inbred lines. Nature. 2010;465:627–31. 10.1038/nature08800 20336072 PMC3023908

[10] Narzisi G, Schatz MC. The challenge of small-scale repeats for indel discovery. Front Bioeng Biotechnol. 2015;3:8 10.3389/fbioe.2015.00008 ; PubMed Central PMCID: PMCPMC4306302. 25674564 PMC4306302

[11] Tan A, Abecasis GR, Kang HM. Unified representation of genetic variants. Bioinformatics. 2015;31(13):2202–4. 10.1093/bioinformatics/btv112 ; PubMed Central PMCID: PMCPMC4481842. 25701572 PMC4481842

[12] Gan X, Stegle O, Behr J, Steffen JG, Drewe P, Hildebrand KL, et al Multiple reference genomes and transcriptomes for Arabidopsis thaliana. Nature. 2011;477:419–23. 10.1038/nature10414 21874022 PMC4856438

[13] Huang W, Massouras A, Inoue Y, Peiffer J, Ràmia M, Tarone AM, et al Natural variation in genome architecture among 205 Drosophila melanogaster Genetic Reference Panel lines. Genome Research. 2014;24:1193–208. 10.1101/gr.171546.113 24714809 PMC4079974

[14] Dembeck LM, Böröczky K, Huang W, Schal C, Anholt RRH, Mackay TFC. Genetic architecture of natural variation in cuticular hydrocarbon composition in Drosophila melanogaster. eLife. 2015;4:e09861 10.7554/eLife.09861 26568309 PMC4749392

[15] Alonso-Blanco C, Andrade J, Becker C, Bemm F, Bergelson J, Borgwardt KM, et al 1,135 Genomes Reveal the Global Pattern of Polymorphism in Arabidopsis thaliana. Cell. 2016;0 10.1016/j.cell.2016.05.063 PMC4949382 27293186

[16] Pacific Biosciences of California I. Sequel System Data Release: Arabidopsis Dataset and Genome Assembly 2016 [https://downloads.pacbcloud.com/public/SequelData/ArabidopsisDemoData/Assembly/Arabidopsis_assembly.fasta].

[17] Keane TM, Goodstadt L, Danecek P, White MA, Wong K, Yalcin B, et al Mouse genomic variation and its effect on phenotypes and gene regulation. Nature. 2011;477(7364):289–94. 10.1038/nature10413 PubMed PMID: WOS:000294852400022. 21921910 PMC3276836

[18] Lee S, Abecasis GR, Boehnke M, Lin X. Rare-variant association analysis: study designs and statistical tests. Am J Hum Genet. 2014;95(1):5–23. 10.1016/j.ajhg.2014.06.009 ; PubMed Central PMCID: PMCPMC4085641. 24995866 PMC4085641

[19] Yalcin B, Wong K, Agam A, Goodson M, Keane TM, Gan X, et al Sequence-based characterization of structural variation in the mouse genome. Nature. 2011;477(7364):326–9. 10.1038/nature10432 ; PubMed Central PMCID: PMCPMC3428933. 21921916 PMC3428933

[20] Sudmant PH, Rausch T, Gardner EJ, Handsaker RE, Abyzov A, Huddleston J, et al An integrated map of structural variation in 2,504 human genomes. Nature. 2015;526:75–81. 10.1038/nature15394 26432246 PMC4617611

[21] Segura V, Vilhjálmsson BJ, Platt A, Korte A, Seren Ü, Long Q, et al An efficient multi-locus mixed-model approach for genome-wide association studies in structured populations. Nature Genetics. 2012;44:825–30. 10.1038/ng.2314 22706313 PMC3386481

[22] Yang J, Benyamin B, McEvoy BP, Gordon S, Henders AK, Nyholt DR, et al Common SNPs explain a large proportion of the heritability for human height. Nature Genetics. 2010;42:565–9. 10.1038/ng.608 20562875 PMC3232052

[23] Visscher PM, Hemani G, Vinkhuyzen AAE, Chen GB, Lee SH, Wray NR, et al Statistical Power to Detect Genetic (Co)Variance of Complex Traits Using SNP Data in Unrelated Samples. Plos Genetics. 2014;10(4). 10.1371/journal.pgen.1004269 PubMed PMID: WOS:000335499600029. 24721987 PMC3983037

[24] Yang J, Bakshi A, Zhu Z, Hemani G, Vinkhuyzen AA, Lee SH, et al Genetic variance estimation with imputed variants finds negligible missing heritability for human height and body mass index. Nat Genet. 2015;47(10):1114–20. 10.1038/ng.3390 ; PubMed Central PMCID: PMCPMC4589513. 26323059 PMC4589513

[25] Kerdaffrec E, Filiault DL, Korte A, Sasaki E, Nizhynska V, Seren U, et al Multiple alleles at a single locus control seed dormancy in Swedish Arabidopsis. Elife. 2016;5 10.7554/eLife.22502 PubMed PMID: WOS:000393418600001. 27966430 PMC5226650

[26] Huang XH, Wei XH, Sang T, Zhao QA, Feng Q, Zhao Y, et al Genome-wide association studies of 14 agronomic traits in rice landraces. Nature Genetics. 2010;42(11):961–U76. 10.1038/ng.695 PubMed PMID: WOS:000283540500012. 20972439

[27] Serrano-Mislata A, Fernández-Nohales P, Doménech MJ, Hanzawa Y, Bradley D, Madueño F. Separate elements of the TERMINAL FLOWER 1 cis-regulatory region integrate pathways to control flowering time and shoot meristem identity. Development. 2016:dev.135269 10.1242/dev.135269 27385013

[28] Bradley D, Ratcliffe O, Vincent C, Carpenter R, Coen E. Inflorescence commitment and architecture in Arabidopsis. Science. 1997;275(5296):80–3. 10.1126/science.275.5296.80 PubMed PMID: WOS:A1997WA90300053. 8974397

[29] Valverde F, Mouradov A, Soppe W, Ravenscroft D, Samach A, Coupland G. Photoreceptor regulation of CONSTANS protein in photoperiodic flowering. Science. 2004;303(5660):1003–6. 10.1126/science.1091761 PubMed PMID: WOS:000188918000043. 14963328

[30] Gnatzy W, Volknandt W, Schulz S. Dufour gland of the digger wasp Liris niger: structure and developmental and biochemical aspects. Cell Tissue Res. 2004;315(1):125–38. 10.1007/s00441-003-0813-2 PubMed PMID: WOS:000188415200011. 14598162

[31] Olaniran OA, Sudhakar AVS, Drijfhout FP, Dublon IAN, Hall DR, Hamilton JGC, et al A Male-Predominant Cuticular Hydrocarbon, 7-Methyltricosane, is used as a Contact Pheromone in the Western Flower Thrips Frankliniella occidentalis. J Chem Ecol. 2013;39(4):559–68. 10.1007/s10886-013-0272-5 PubMed PMID: WOS:000317606200011. 23519504

[32] Blackwell E, Halatek IM, Kim HJ, Ellicott AT, Obukhov AA, Stone DE. Effect of the pheromone-responsive G(alpha) and phosphatase proteins of Saccharomyces cerevisiae on the subcellular localization of the Fus3 mitogen-activated protein kinase. Mol Cell Biol. 2003;23(4):1135–50. 10.1128/MCB.23.4.1135-1150.2003 ; PubMed Central PMCID: PMCPMC141143. 12556475 PMC141143

[33] Xiang Y, Song B, Née G, Kramer K, Finkemeier I, Soppe W. Sequence Polymorphisms at the Reduced Dormancy 5 Pseudophosphatase Underlie Natural Variation in Arabidopsis Dormancy. Plant Physiology. 2016:pp.005252016. 10.1104/pp.16.00525 27288362 PMC4972279

[34] Barboza L, Effgen S, Alonso-Blanco C, Kooke R, Keurentjes JJB, Koornneef M, et al Arabidopsis semidwarfs evolved from independent mutations in GA20ox1, ortholog to green revolution dwarf alleles in rice and barley. Proceedings of the National Academy of Sciences. 2013;110:15818–23. 10.1073/pnas.1314979110 24023067 PMC3785751

[35] Alcázar R, García AV, Kronholm I, de Meaux J, Koornneef M, Parker JE, et al Natural variation at Strubbelig Receptor Kinase 3 drives immune-triggered incompatibilities between Arabidopsis thaliana accessions. Nature Genetics. 2010;42:1135–9. 10.1038/ng.704 21037570

[36] Rivas MA, Beaudoin M, Gardet A, Stevens C, Sharma Y, Zhang CK, et al Deep resequencing of GWAS loci identifies independent rare variants associated with inflammatory bowel disease. Nat Genet. 2011;43(11):1066–73. 10.1038/ng.952 ; PubMed Central PMCID: PMCPMC3378381. 21983784 PMC3378381

[37] Schmalenbach I, Zhang L, Ryngajllo M, Jimenez-Gomez JM. Functional analysis of the Landsberg erecta allele of FRIGIDA. Bmc Plant Biology. 2014;14 10.1186/s12870-014-0218-2 PubMed PMID: WOS:000341317900001. 25207670 PMC4158083

[38] Shindo C, Aranzana MJ, Lister C, Baxter C, Nicholls C, Nordborg M, et al Role of FRIGIDA and FLOWERING LOCUS C in Determining Variation in Flowering Time of Arabidopsis. Plant Physiology. 2005;138:1163–73. 10.1104/pp.105.061309 15908596 PMC1150429

[39] Grimm DG, Roqueiro D, Salome P, Kleeberger S, Greshake B, Zhu W, et al easyGWAS: A Cloud-based Platform for Comparing the Results of Genome-wide Association Studies. Plant Cell. 2016 10.1105/tpc.16.00551 . 27986896 PMC5304348

[40] Cannavo E, Koelling N, Harnett D, Garfield D, Casale FP, Ciglar L, et al Genetic variants regulating expression levels and isoform diversity during embryogenesis. Nature. 2017;541(7637):402–6. 10.1038/nature20802 . 28024300

[41] Kilpinen H, Goncalves A, Leha A, Afzal V, Alasoo K, Ashford S, et al Common genetic variation drives molecular heterogeneity in human iPSCs. Nature. 2017;546(7658):370–5. 10.1038/nature22403 ; PubMed Central PMCID: PMCPMC5524171. 28489815 PMC5524171

[42] Kawakatsu T, Huang S-sC, Jupe F, Sasaki E, Schmitz RJ, Urich MA, et al Epigenomic Diversity in a Global Collection of Arabidopsis thaliana Accessions. Cell. 2016;166:492–505. 10.1016/j.cell.2016.06.044 27419873 PMC5172462

[43] Brogna S, Wen JK. Nonsense-mediated mRNA decay (NMD) mechanisms. Nat Struct Mol Biol. 2009;16(2):107–13. 10.1038/nsmb.1550 PubMed PMID: WOS:000263286600005. 19190664

[44] Borsani O, Zhu JH, Verslues PE, Sunkar R, Zhu JK. Endogenous siRNAs derived from a pair of natural cis-antisense transcripts regulate salt tolerance in Arabidopsis. Cell. 2005;123(7):1279–91. 10.1016/j.cell.2005.11.035 PubMed PMID: WOS:000234584500016. 16377568 PMC3137516

[45] Lamesch P, Berardini TZ, Li D, Swarbreck D, Wilks C, Sasidharan R, et al The Arabidopsis Information Resource (TAIR): improved gene annotation and new tools. Nucleic Acids Research. 2012;40:D1202–D10. 10.1093/nar/gkr1090 22140109 PMC3245047

[46] Katoh K, Standley DM. MAFFT Multiple Sequence Alignment Software Version 7: Improvements in Performance and Usability. Molecular Biology and Evolution. 2013;30:772–80. 10.1093/molbev/mst010 23329690 PMC3603318

[47] Benoist C, O'Hare K, Breathnach R, Chambon P. The ovalbumin gene—sequence of putative control regions. Nucleic Acids Research. 1980;8:127–42. 10.1093/nar/8.1.127 6243777 PMC327247

[48] Purcell S, Neale B, Todd-Brown K, Thomas L, Ferreira MAR, Bender D, et al PLINK: A Tool Set for Whole-Genome Association and Population-Based Linkage Analyses. The American Journal of Human Genetics. 2007;81:559–75. 10.1086/519795 17701901 PMC1950838

[49] Fusi N, Lippert C, Lawrence ND, Stegle O. Warped linear mixed models for the genetic analysis of transformed phenotypes. Nature Communications. 2014;5:4890 10.1038/ncomms5890 25234577 PMC4199105

[50] Lippert C, Listgarten J, Liu Y, Kadie CM, Davidson RI, Heckerman D. FaST linear mixed models for genome-wide association studies. Nature Methods. 2011;8:833–5. 10.1038/nmeth.1681 21892150

[51] Team RC. R: A language and environment for statistical computing. R Foundation for Statistical Computing.

[52] Kang HM, Zaitlen NA, Wade CM, Kirby A, Heckerman D, Daly MJ, et al Efficient Control of Population Structure in Model Organism Association Mapping. Genetics. 2008;178:1709–23. 10.1534/genetics.107.080101 18385116 PMC2278096

[53] Du Z, Zhou X, Ling Y, Zhang ZH, Su Z. agriGO: a GO analysis toolkit for the agricultural community. Nucleic Acids Research. 2010;38:W64–W70. 10.1093/nar/gkq310 PubMed PMID: WOS:000284148900012. 20435677 PMC2896167

