# Supplementary material for: Recovery of novel association loci in Arabidopsis thaliana and Drosophila melanogaster through leveraging INDELs association and integrated burden test
Source: PLoS Genet. 2018 Oct 16;14(10):e1007699. doi: 10.1371/journal.pgen.1007699 (PMC6203403; doi:10.1371/journal.pgen.1007699)
Supplement: S1 Text — (PDF) [file pgen.1007699.s001.pdf]

1. Portal for accessing our GWAS analysis results

Web site: Software Irisas and the source code are available from https://github.com/baoxingsong/Irisas. The tables of synchronized variants for *Arabidopsis thaliana* and *Drosophila melanogaster* are available from our web site http://chi.mpipz.mpg.de/gwas.

Genome Scan Browser: The detailed association results are available by LocusZoom [1] from our website http://chi.mpipz.mpg.de/gwas.

Scripts: The scripts and numerical data, we used to draw the plots, are available from https://github.com/baoxingsong/GWAS/tree/master/pg.

2. Genomic variants evaluation using long reads based assembly

We downloaded the *de novo* assembled genome sequence of L*er*-0 [2]. The genome was aligned against the TAIR10 reference using Blat v3.5 [3] (with –minIdentity=25). The alignment was then curated manually by checking its consistency with the Illumina short reads alignment and regions showing very little consistency were discarded. We define a variant as true positives (TP) if it is confirmed by the assembly, false positives (FP) otherwise. A variant shown by the assembly but absent from our variant table is false negatives (FN). The false discovery rate (FDR) is defined as FP/(TP+FP), the false negative rate (FNR) is defined as FN/(FN+TP) and the true positive rate (TPR) is defined as TP/(TP+FN). Overall for a total length of 82.2Mbp regions, the FDR of SNPs was 4.5% and FDR of INDEL was 7.6%, the TPR of SNPs was 94.3% and TPR of INDELs was 90.9%, the FNR of SNPs was 5.7% and FNR of INDELs was 9.1%.

By the alignment of L*er*-0 Illumina sequencing (16.5Gbp after removing of duplication reads), we then defined genome regions as accessible[4] where the average mapping depth was higher than 2 and smaller than 81, and inaccessible otherwise. For accessible regions of 77.6Mbp, the FDR of SNPs was 2.5% and the FDR of INDEL was 5.7%, the TPR of SNPs was 98.7% and the TPR of INDELs was 92.9%, the FNR of SNPs was 1.3% and the FNR of INDELs was 7.1%.

3. Statistical analysis

The genome-wide density distribution of variants

After removing homogeneous lines, 162 *A. thaliana* accessions and 207 *D. melanogaster* lines was used

to explore variants density. The density distribution was collected with a bin size 0.5M for each accession independently. And the numbers of variants for each bin from every accession were summed and plotted.

Decay of linkage disequilibrium

We used the 162 *A. thaliana* and 207 *D. melanogaster* samples. Since ambiguous (potentially heterozygous) calls were encoded as missing, no haplotype phasing was necessary. Additionally, variants were excluded if MAF less than 0.1 or missing rate was above 0.5. For the LD pattern of ORFSs of *A. thaliana*, only the protein coding transcripts with ID ended with ".1" were employed. These variants were used as input for the program PLINK to compute the linkage disequilibrium (LD) as measured by r^2.

We fit the r^2 values and physical distances on the chromosome into the fourth moments at two loci model [5]:

$$\mathrm{E}(r^2) = \left[\frac{10+Cd}{(2+Cd)(11+Cd)}\right]\left[1+\frac{(3+Cd)(12+12Cd+(Cd)^2)}{n(2+Cd)(11+Cd)}\right] \quad (13)$$

Where $E(r^2)$ is the expect value of r^2 and n is the number of inbred individuals. C is a parameter which is directly proportional to the ratio of recombination rate per base pair between sites to the effective population size. Let A, B, D be alleles at ordered loci and C_{AB} be the recombination rate between A and B. We have $C_{AD}=C_{AB}+C_{BD}$. So, the recombination fraction between any two variants is Cd, where d is the distance between these two variants. Here, the value of C was estimated to minimize $\sum(r^2 - E(r^2))^2$ with the *nls* function implemented in *R*. The LD half-decay distances, i.e., the distance at which LD is half of its maximum value, were estimated as the distance where $E(r^2)$ halved its maximum value.

4. Estimation of the threshold value for the genome-wide significance with permutation tests

For each genotype, the phenotypic values were shuffled with the function "sample" implemented in R and used for the association test. The process was repeated for 1000 times. The minimum *p*-value for each permutation were collected and the value corresponding to the 5% lower tail of those 1000 p-values were selected as the threshold value for the genome-wide significance of our study.

5. *A. thaliana* eQTL analysis

The leaf transcriptomes of 728 accessions were obtained from the public database [6] and their normalized expression profiles were used for our eQTLs. To determine the technical covariates, principal component analysis [7] (PCA) was performed for the expression levels as suggested by original paper. The outliers and strong cluster effects due to the batch effect were detected (S11 Fig a). After removing 1 outlier and 98 homogeneous lines, PC1 (principal component 1) and PC2 explained 19.42% and 8.05% variance respectively (S11 Fig b). The expression levels were adjusted for PC1 and PC2 with regression analysis. The adjusted values of each gene were quartile normalized and used as the dependent variable in the linear mixed association model implemented in EMMAX. We performed routine filtering based on MAF, minor allele number, missing rate and removed variants in the centromere region. 3869962 SNPs, 1962336 INDELs and 14239 ORFSs were used as independent variables for association analysis. The SNPs were further filtered with LD, and 2250198 SNPs were left to construct kinship matrix. We only performed association analyses for those genes expressed in more than 529 out of the 628 accessions.

6. The benefit of MSA to GWAS using single marker

As mentioned before, INDELs could have multiple alignment isomorphs, and this ambiguity would undermine the INDEL-based association analysis. We proposed consistent variant calling using multiple sequence alignment as a necessary step for the INDEL association. To check the benefit of MSA on single marker GWAS analysis for *A. thaliana*, we compared the GWAS results without consistent variant calling with the GWAS results using variant tables from consistent variant calling. The result suggested consistent variant calling improved the power of association analysis and could uncover some associated loci which could not be detected without it (S13 Fig).

7. Simulation of the effect of common independent-loss-of-function causal alleles

To evaluate the power of gene ORFSs for the association analysis, we simulated a simple gene with two ORF shift INDELs which shift the ORF independently. We used a model with 3 alleles: functional allele 1, loss of function allele 2 with ORF shifted by INDEL B and loss of function allele 3 with ORF shifted by INDEL C (S12 Fig a). We assume all three alleles are evenly distributed within a population of 210 samples. The phenotypes of three alleles follow Gaussian distribution $N(2, 1.44)$, $N(1, 1.44)$ and $N(1, 1.44)$. The simulation was repeated for $10,000$ times and associations were performed with Wilcoxon

rank sum test as suggested[8]. The result suggested ORFSs based association have much higher power than direct variant calling based association method (S12 Fig b).

8. Phenotyping validation

Phenotyping of *tfl1* mutation lines

The *tlf1-1*, *tlf1-13* and wide type (NASC ID: N70000) seeds were sowed under long day (16h daylight) at 21°C. The 5 days old seedlings were moved into 4°C short day (8h day time) for vernalization and moved back to long day after 14 days. The plants were checked every day between 1 p.m. to 4 p.m. for flowering time related phenotypes (number of days before bolting, number of days before bolting reach 5cm high and number of days before first flower observable).

Phenotyping of *svp* mutation lines

The seeds of *svp-41* mutation line and wide type (NASC ID: N70000) were sowed under long day (16h daylight) conditions at four different temperatures. The plants were checked every day between 1 p.m. to 4 p.m. for flowering time related phenotypes.

URLs:

The *A. thaliana* expression profiles:

ftp://ftp.ncbi.nlm.nih.gov/geo/series/GSE80nnn/GSE80744/suppl/GSE80744_ath1001_tx_norm_2016-04-21-UQ_gNorm_normCounts_k4.tsv.gz

The de novo assembly of L*er*-0 PacBio sequencing:

https://downloads.pacbcloud.com/public/SequelData/ArabidopsisDemoData/Assembly/Arabidopsis_assembly.fasta

IMR/DENOM Version v.0.5.0: http://chi.mpipz.mpg.de/imrdenom/

Reference:

1. Pruim RJ, Welch RP, Sanna S, Teslovich TM, Chines PS, Gliedt TP, et al. LocusZoom: regional visualization of genome-wide association scan results. Bioinformatics. 2010;26(18):2336-7. doi: 10.1093/bioinformatics/btq419. PubMed PMID: 20634204; PubMed Central PMCID: PMCPMC2935401.

2. Kim KE, Peluso P, Babayan P, Yeadon PJ, Yu C, Fisher WW, et al. Long-read, whole-genome shotgun

sequence data for five model organisms. Sci Data. 2014;1:140045. doi: 10.1038/sdata.2014.45. PubMed PMID: 25977796; PubMed Central PMCID: PMCPMC4365909.

3. Kent WJ. BLAT--the BLAST-like alignment tool. Genome Res. 2002;12(4):656-64. doi: 10.1101/gr.229202. Article published online before March 2002. PubMed PMID: 11932250; PubMed Central PMCID: PMCPMC187518.

4. Keane TM, Goodstadt L, Danecek P, White MA, Wong K, Yalcin B, et al. Mouse genomic variation and its effect on phenotypes and gene regulation. Nature. 2011;477(7364):289-94. doi: 10.1038/nature10413. PubMed PMID: WOS:000294852400022.

5. Hill WG, Weir BS. Variances and covariances of squared linkage disequilibria in finite populations. Theor Popul Biol. 1988;33(1):54-78. PubMed PMID: 3376052.

6. Kawakatsu T, Huang S-sC, Jupe F, Sasaki E, Schmitz RJ, Urich MA, et al. Epigenomic Diversity in a Global Collection of Arabidopsis thaliana Accessions. Cell. 2016;166:492-505. doi: 10.1016/j.cell.2016.06.044.

7. Lappalainen T, Sammeth M, Friedländer MR, Hoen PACt, Monlong J, Rivas MA, et al. Transcriptome and genome sequencing uncovers functional variation in humans. Nature. 2013;501:506-11. doi: 10.1038/nature12531.

8. Atwell S, Huang YS, Vilhjálmsson BJ, Willems G, Horton M, Li Y, et al. Genome-wide association study of 107 phenotypes in Arabidopsis thaliana inbred lines. Nature. 2010;465:627-31. doi: 10.1038/nature08800.
