# Supplementary figures and images for: Recovery of novel association loci in Arabidopsis thaliana and Drosophila melanogaster through leveraging INDELs association and integrated burden test
Source: PLoS Genet. 2018 Oct 16;14(10):e1007699. doi: 10.1371/journal.pgen.1007699 (PMC6203403; doi:10.1371/journal.pgen.1007699)

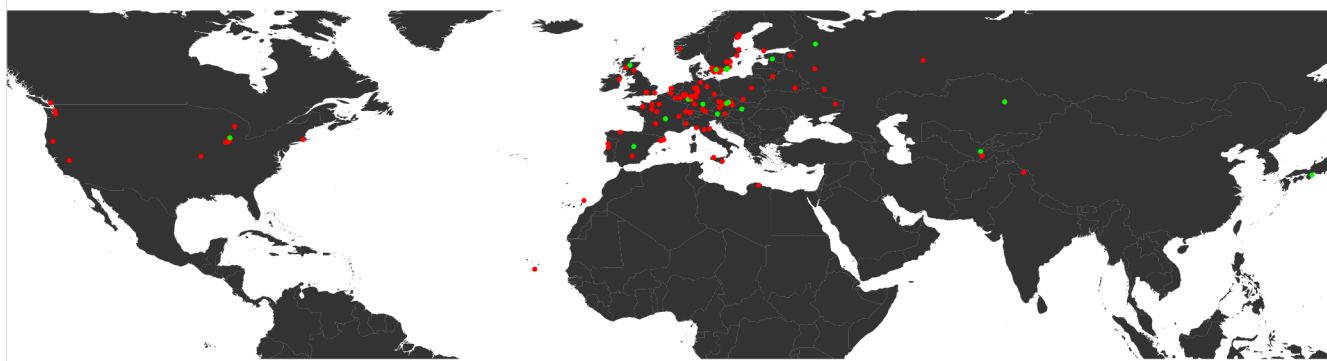

Supplement: S1 Fig — Those in green are accessions whose Illumina shotgun sequence data are unavailable, and thus excluded from association analysis in this study. (PDF) [file pgen.1007699.s002.pdf]

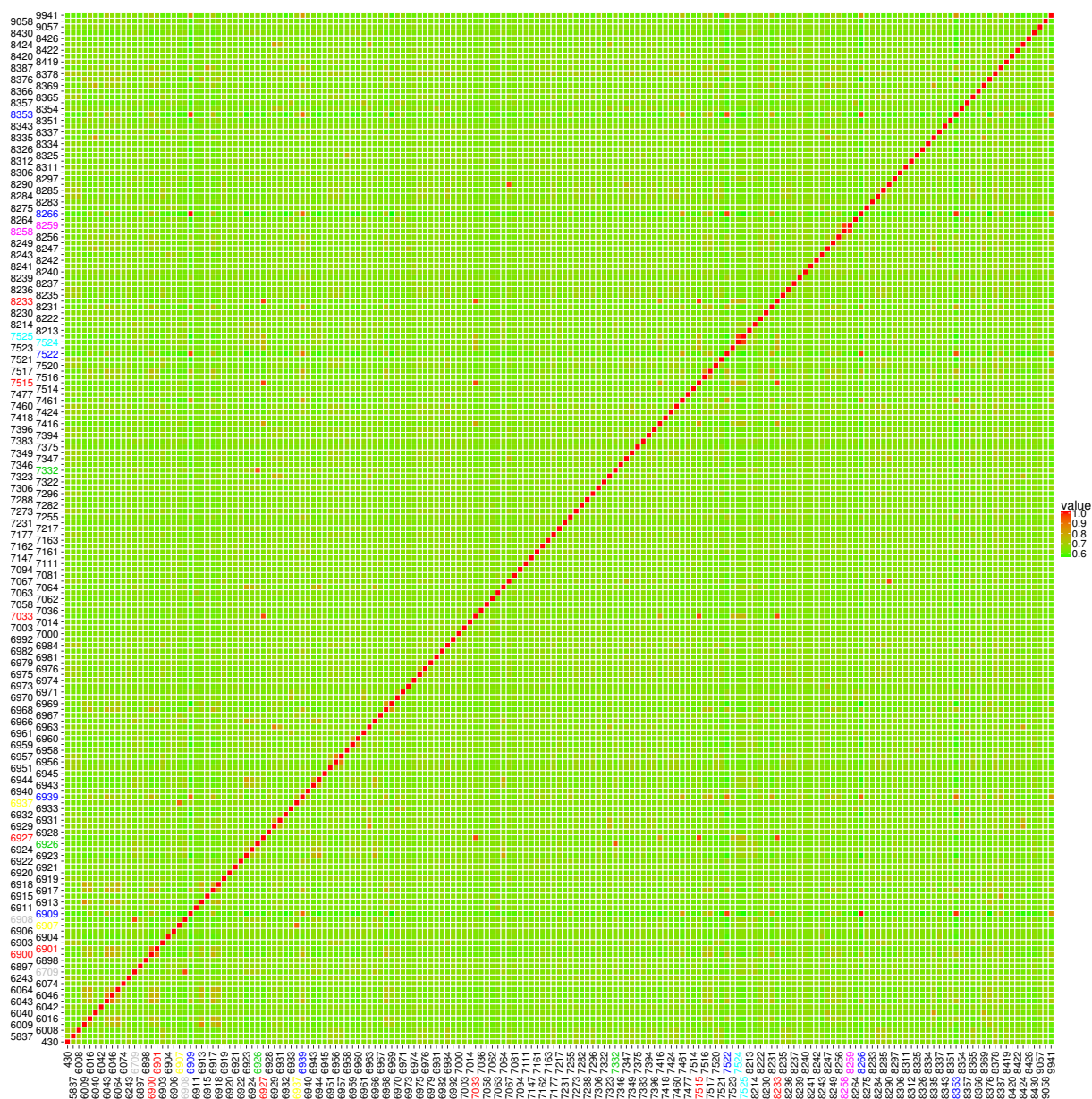

Supplement: S2 Fig — Accessions were indicated with ecotype ID and those accessions colored black had no very similar accessions detected. And those similar accessions were labeled with same color. (PDF) [file pgen.1007699.s003.pdf]

**a**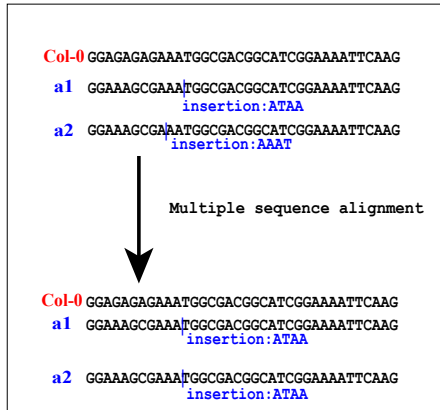**b**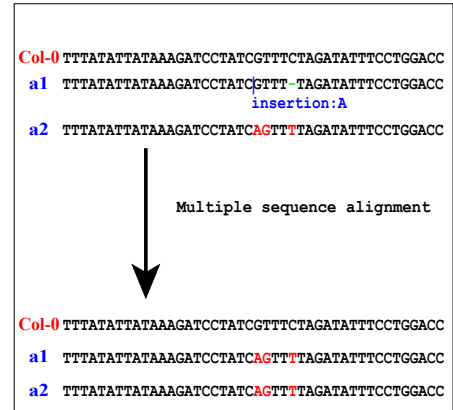**c**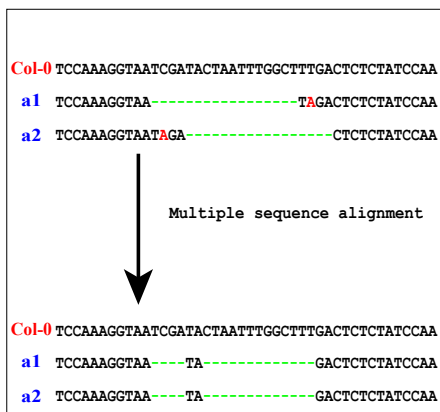

Supplement: S3 Fig — (a) An INDEL can be placed at multiple positions and could be unified with available left alignment algorithm. (b) A haplotype could be represented with different type of variants. (c) A haplotype could be represented with several different INDEL/SNP combinations. (PDF) [file pgen.1007699.s004.pdf]

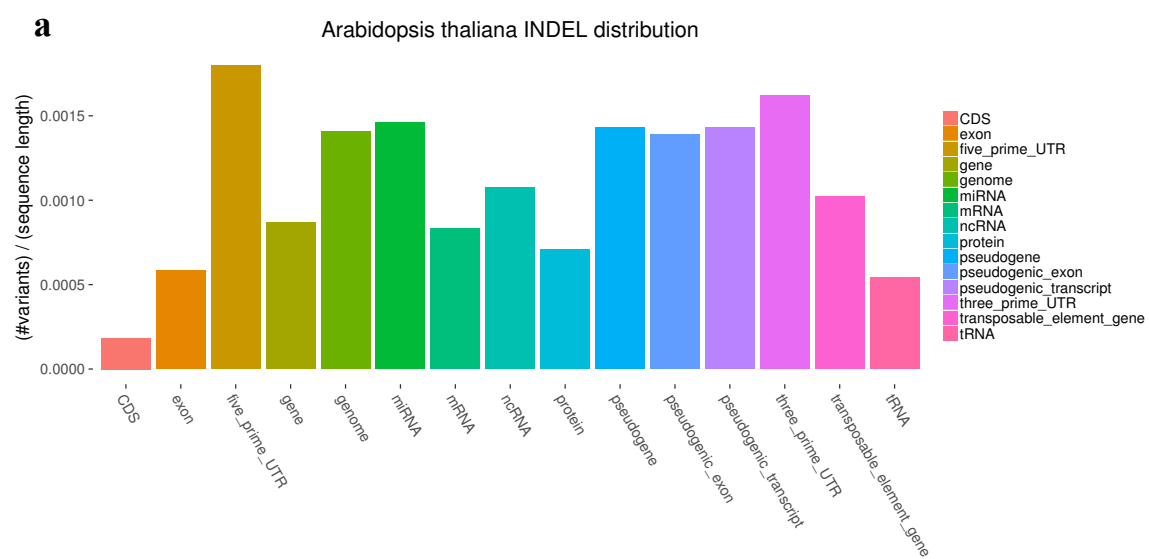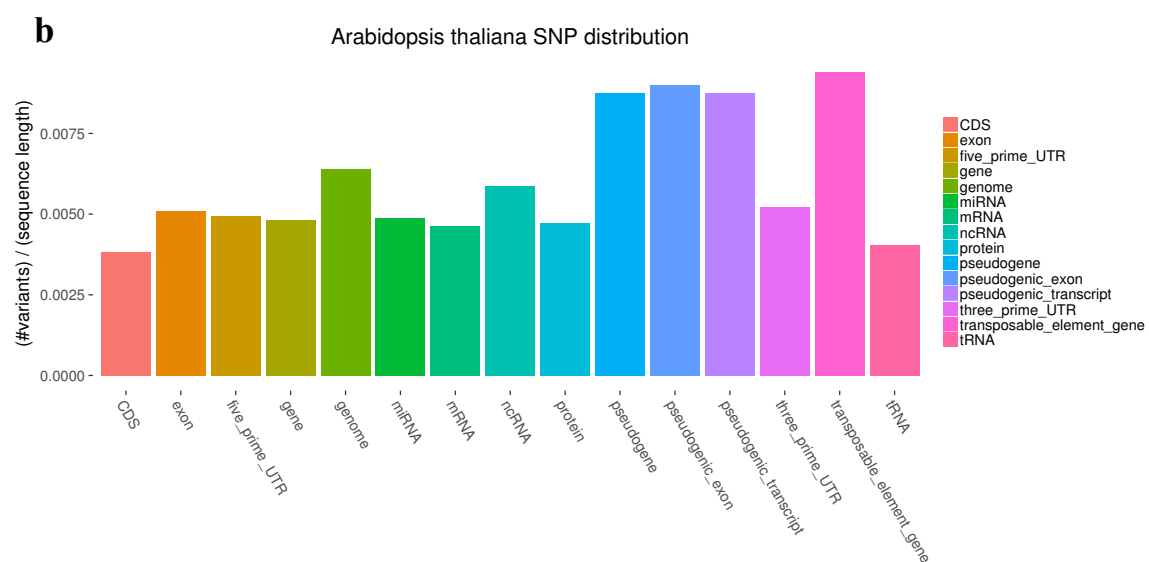

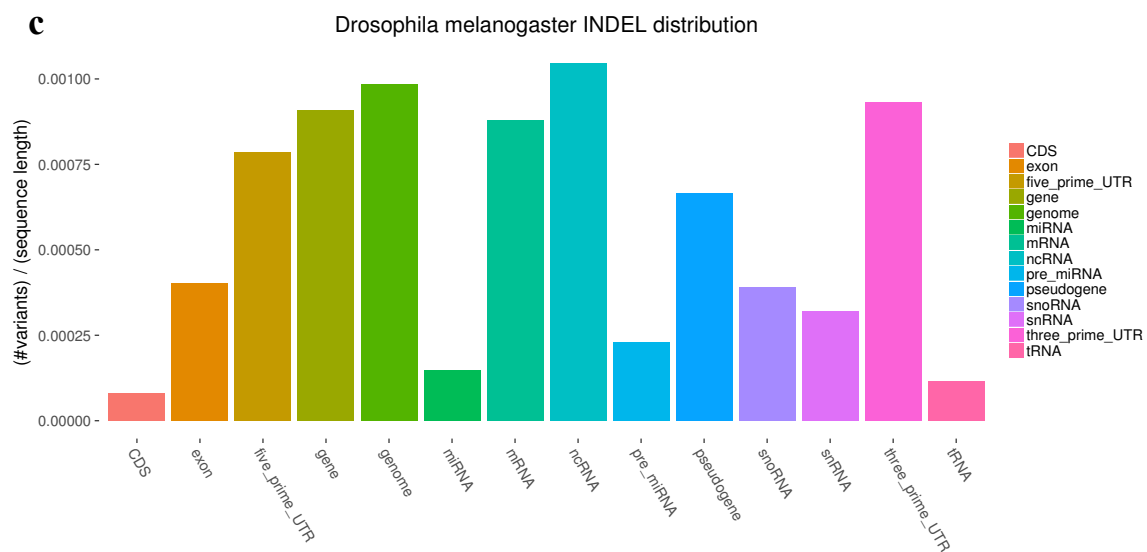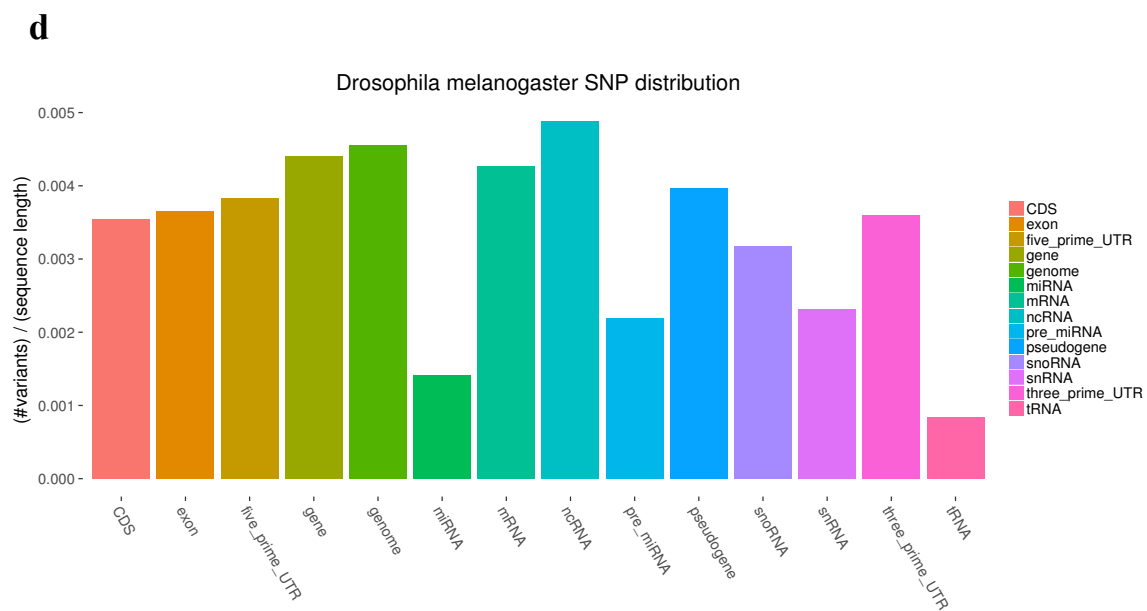

Supplement: S4 Fig — (a, b) SNPs and INDELs distribution of A. thaliana. (c, d) SNPs and INDELs distribution of D. melanogaster. Relatively less INDELs and SNPs have been observed in CDS regions comparing with other genomic regions. (PDF) [file pgen.1007699.s005.pdf]

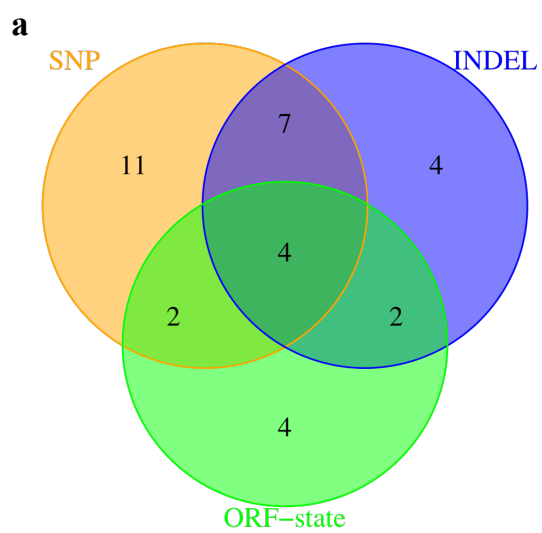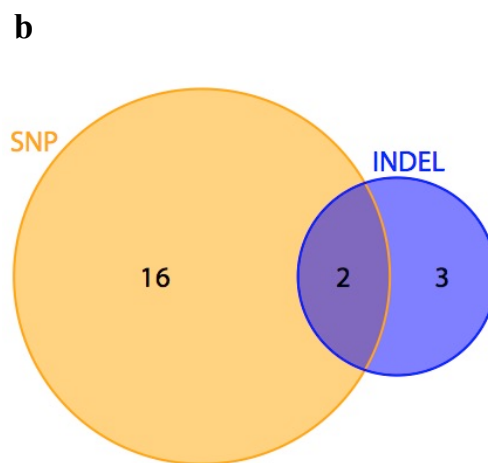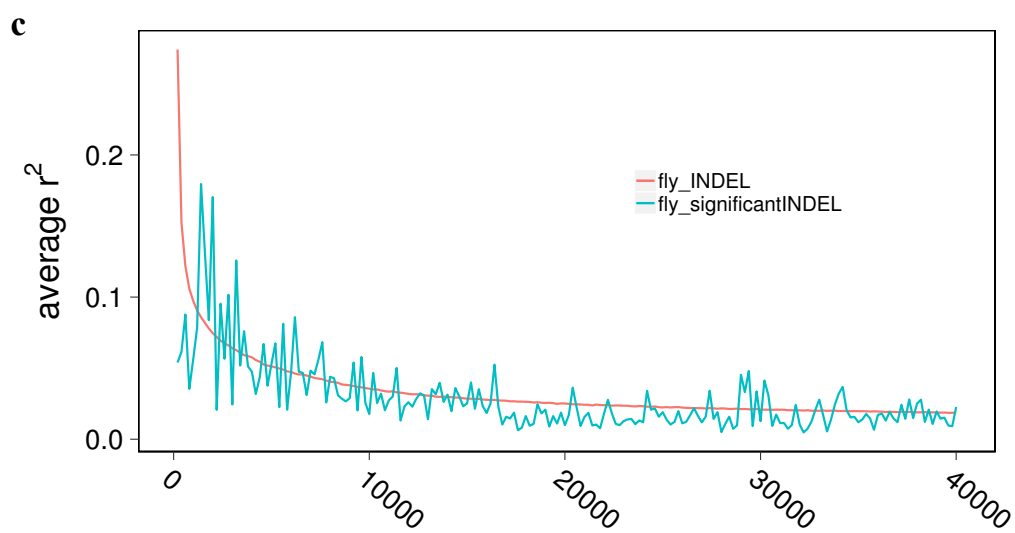

Supplement: S5 Fig — (a) The venn diagram for phenotypes of A. thaliana with different QTLs using three genotypes. (b) The venn diagram for phenotypes of D. melanogaster with different QTLs using three genotypes. (c) LD decay patterns of different types of D. melanogaster INDELs, *significantINDEL for the significant INDELs against their nearby SNPs and INDELs. (PDF) [file pgen.1007699.s006.pdf]

**a**

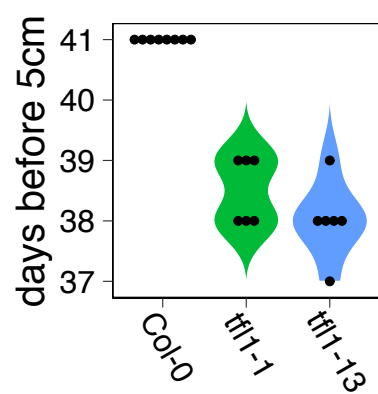

**b**

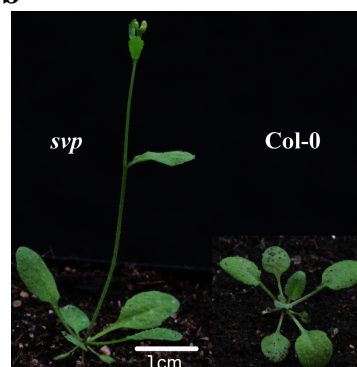

Supplement: S6 Fig — (a) The functional validation of TFL1 locus by comparing two tfl1 T-DNA mutation lines to the wild-type Col-0 accession. (b) The functional validation of SVP locus by comparing svp T-DNA mutation lines to the wild-type Col-0 accession. (16 days old plants grown under the condition specified in the original association study). (PDF) [file pgen.1007699.s007.pdf]

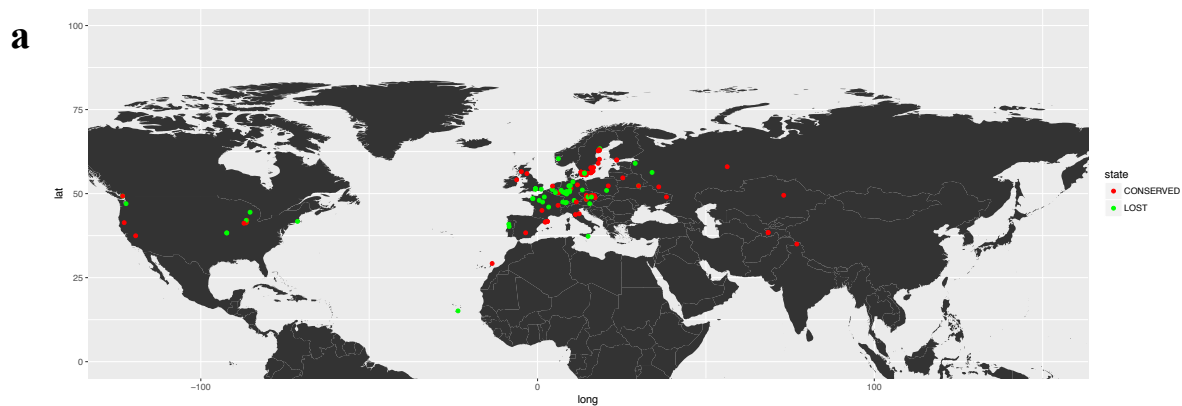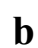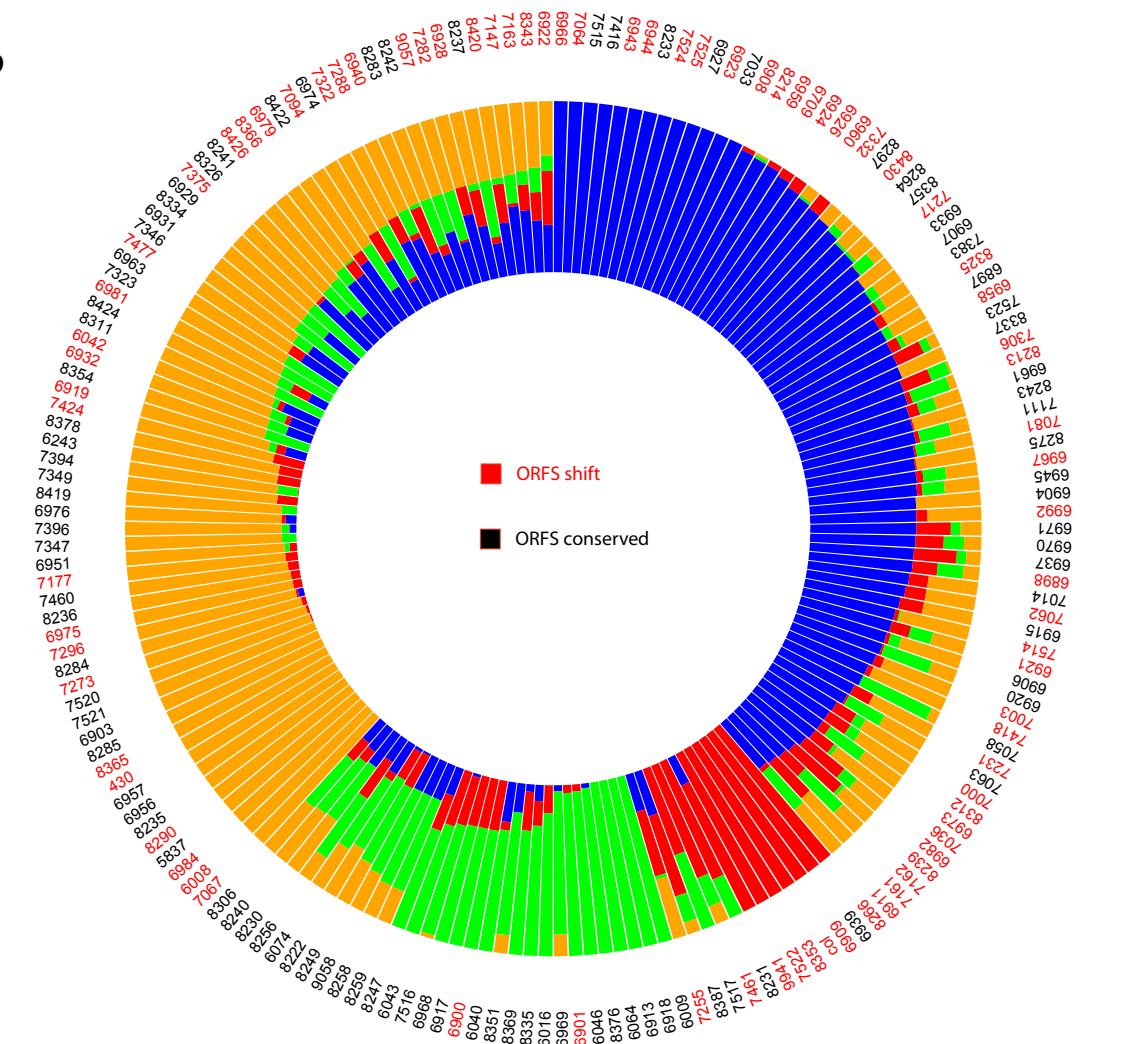

Supplement: S7 Fig — (a) The geographical distribution of predicated functional and loss-of-function FRI alleles. (b) The sub-population distribution of predicated functional and loss-of-function FRI alleles. (c) The geographical distribution of TFL1 nearby significant INDEL. (d) The sub-population distribution of TFL1 nearby significant INDEL. (PDF) [file pgen.1007699.s008.pdf]

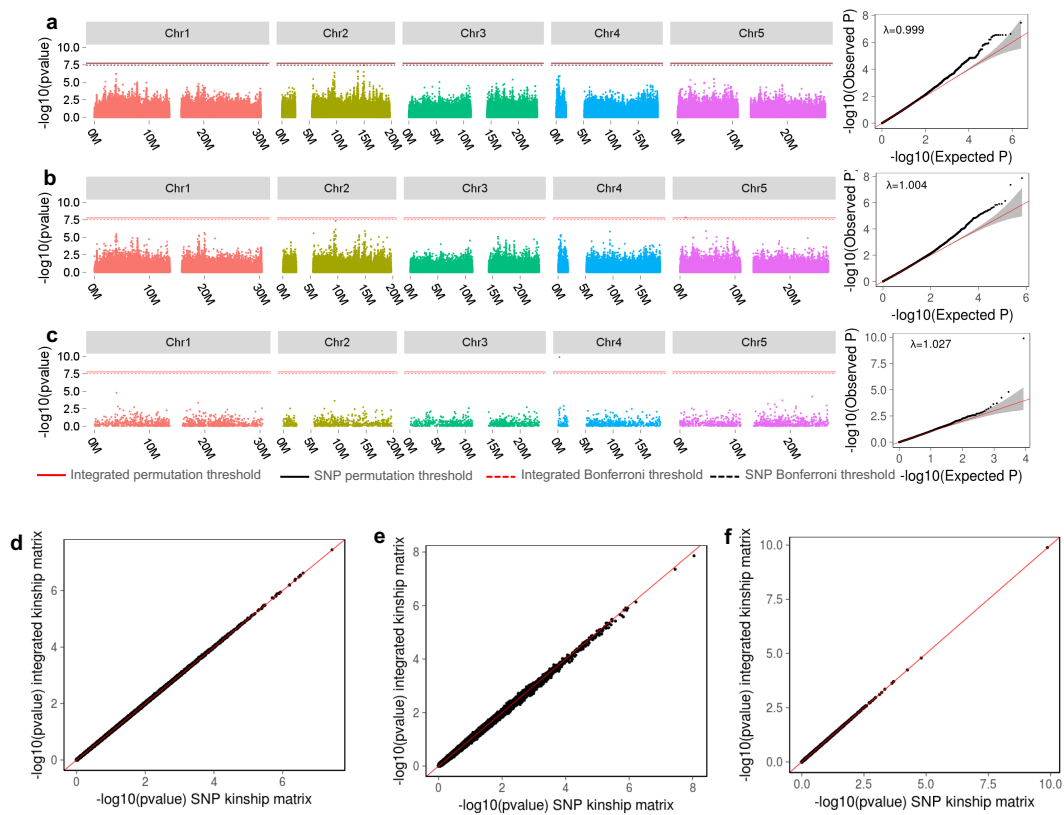

Supplement: S8 Fig — (a-c) The Manhattan and quantile-quantile (QQ) plot using SNPs (a), INDELs (b) and ORFSs (c) for phenotype “number of days required for the bolt height to reach 5cm with 2 weeks vernalization” when using kinship matrix constructed from combined variants of SNP and INDEL. (d-f) Comparing the -log10(pvalue) of SNP (d), INDEL (e), ORFS (f) using kinship matrix constructed from SNP variants versus combined variants. (PDF) [file pgen.1007699.s009.pdf]

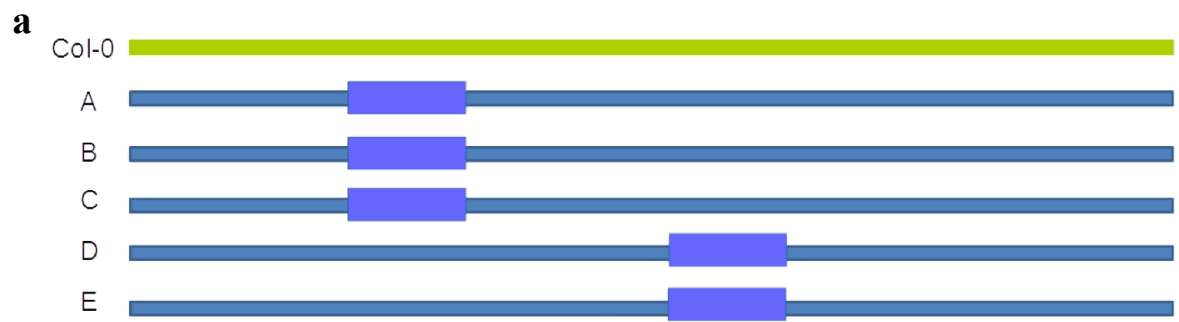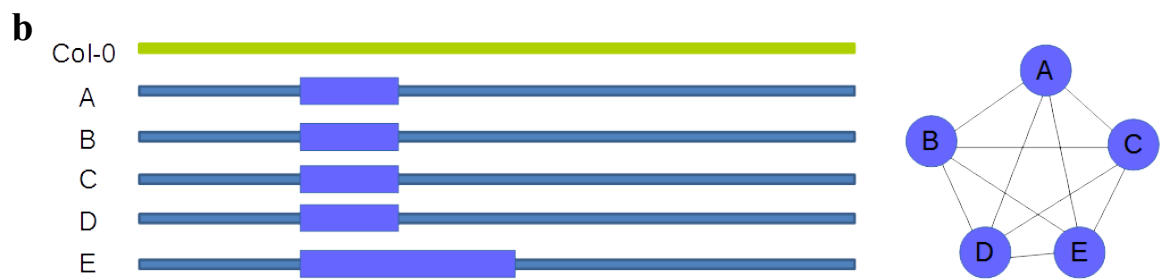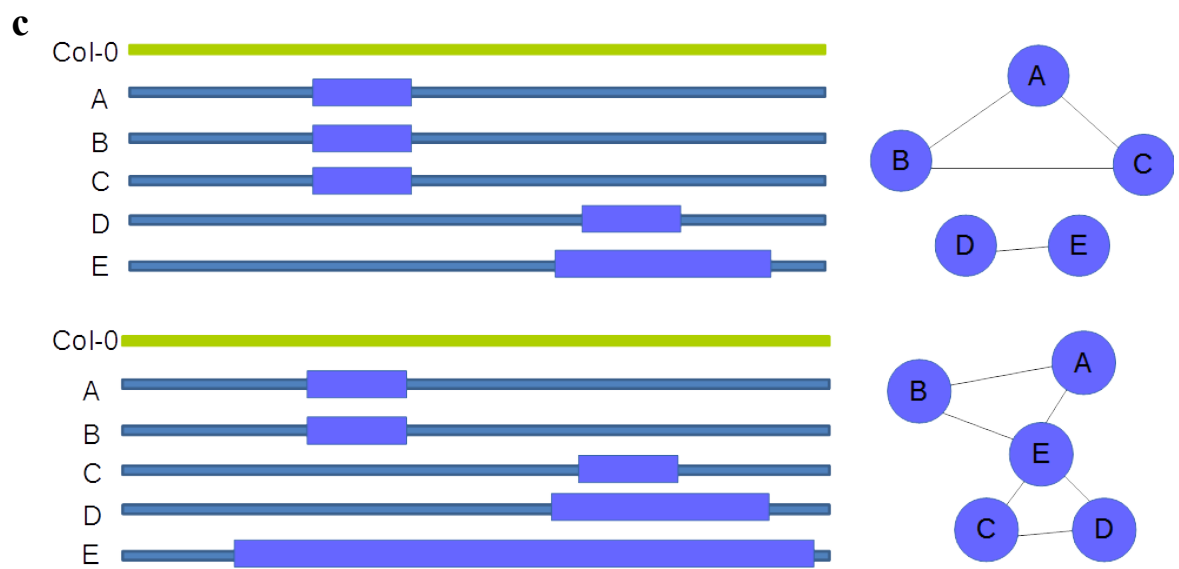

Supplement: S9 Fig — (a) A cartoon shows how to infer the independence of ORF-shift variants from sequence diversity. (b) An example of dependent ORF-shift variants. (c) Examples of two independent ORF-shift variants. (PDF) [file pgen.1007699.s010.pdf]

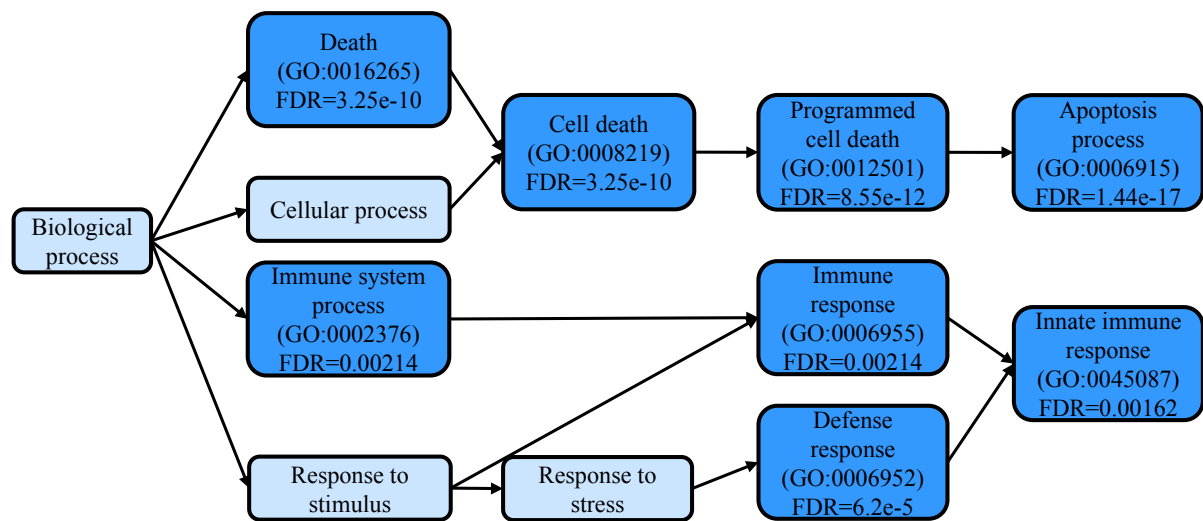

Supplement: S10 Fig — (PDF) [file pgen.1007699.s011.pdf]

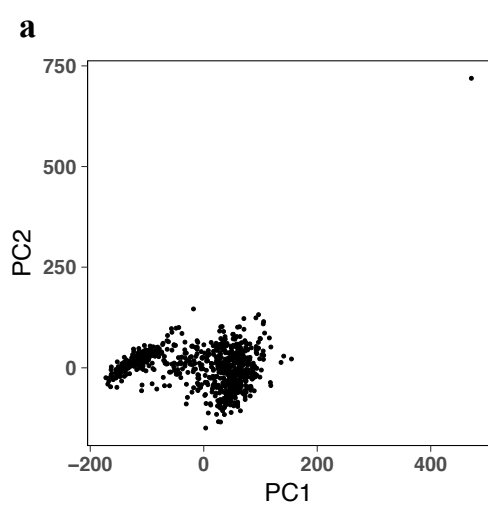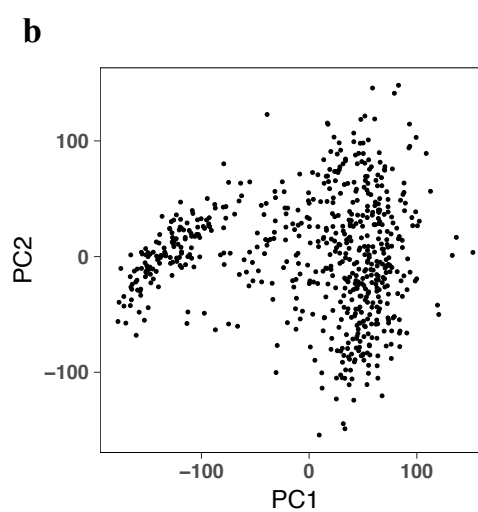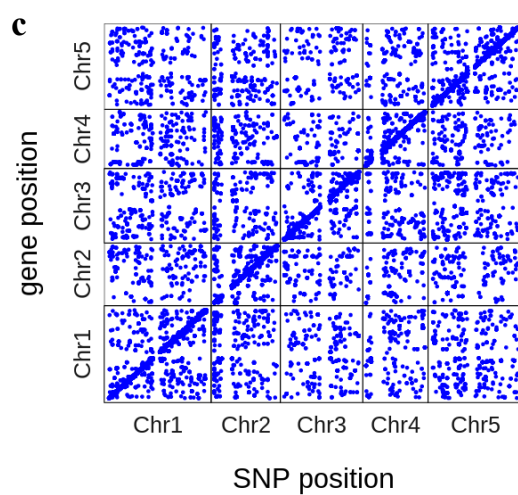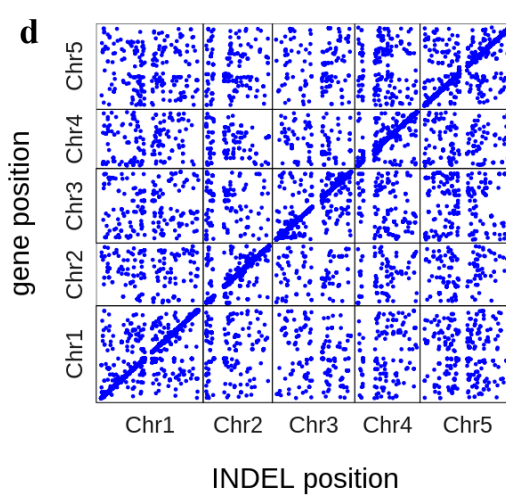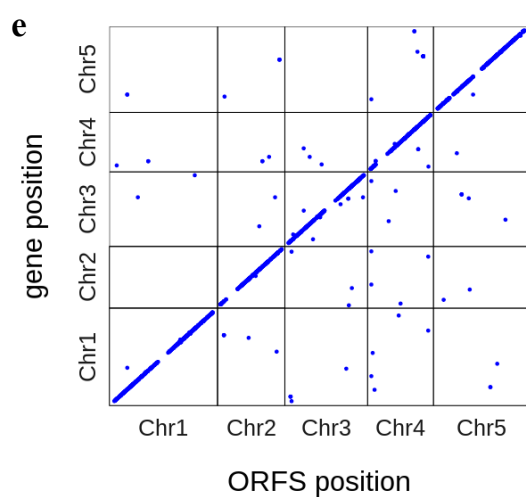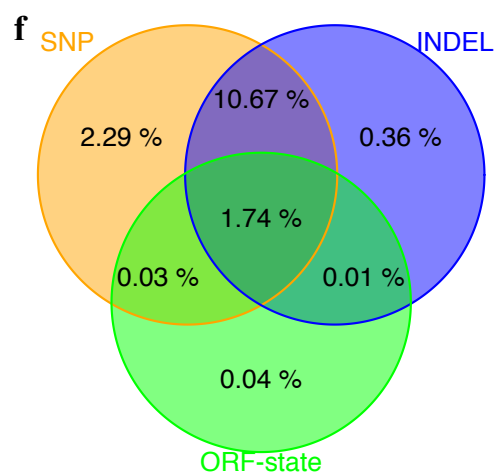

**g**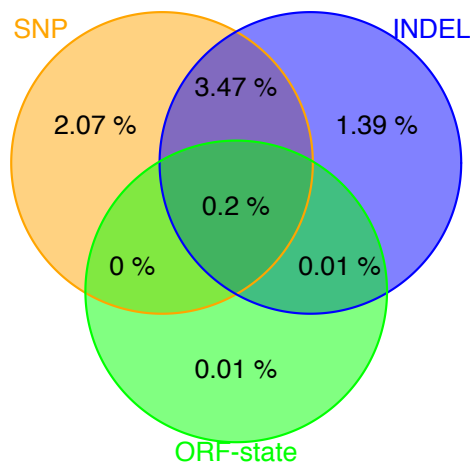**h**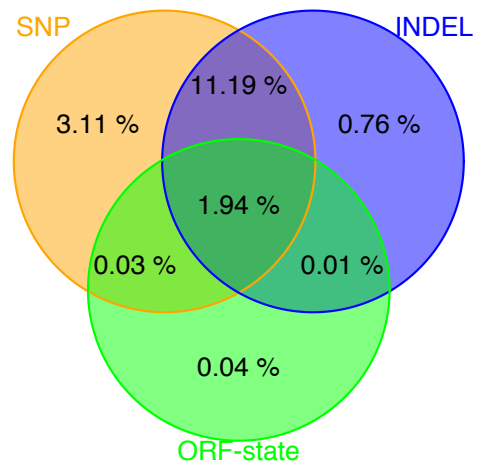**i**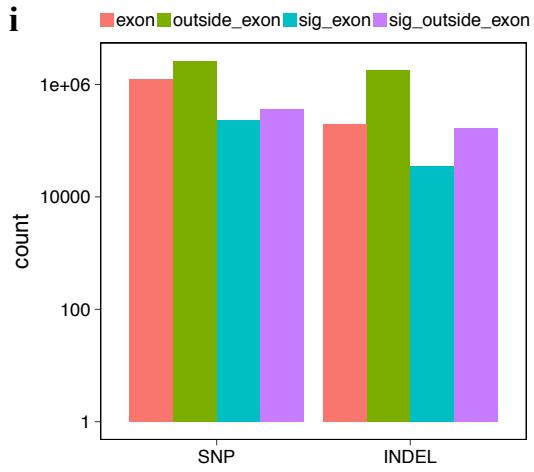

Supplement: S11 Fig — (a) Principal component analysis (PCA) of the expression matrix. The expression values were log10 transformed before analysis. (b) PCA of the expression matrix from 628 accessions after removing outliers and homogeneous individuals. (c) The start position of the mapped genes was plotted against the chromosome position of the associated SNPs. (d) The start position of the mapped genes was plotted against the chromosome position of the associated INDELs. (e) The start position of the mapped genes was plotted against the chromosome position of the associated ORFSs. (f) The variance being explained by cis-eQTLs for the expression level of A. thaliana genes with eQTL detected. (g) The variance being explained by trans-eQTLs for the expression level of A. thaliana genes with eQTL detected. (h) The variance being explained by all eQTLs for the expression level of A. thaliana genes with eQTL detected. (i) The count of snpQTLs and indelQTLs located in exon region against those of SNPs and INDELs outside exon region. (PDF) [file pgen.1007699.s012.pdf]

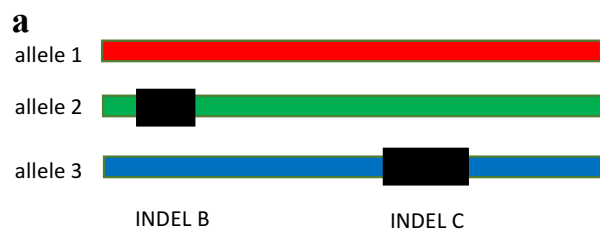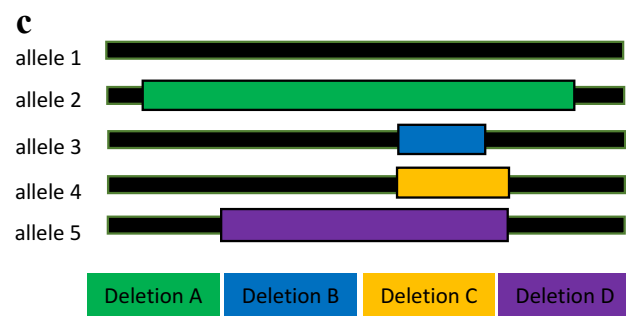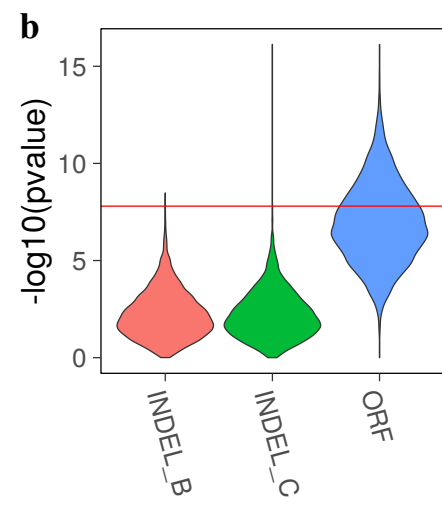

Supplement: S12 Fig — (a) A cartoon indicates an independent loss-of-function gene, with box represent CDS sequence. The allele 1 is a functional allele. Allele 2 and allele 3 indicate loss-of-function allele due to ORF of them were shifted by INDEL B and INDEL C independently. (b) Phenotypes of allele 1 were simulated with mean 2, variance 1.44 and 70 samples. Phenotypes of allele 2 and allele 3 were simulated with mean value 1, variance 1.44 and 70 samples. The association based on present/absent of single INDEL and ORF states were performed with Wilcoxon rank sum test. This process was repeated for 10, 000 times, and the corresponding p-values were illustrated with a violin plot. The value of red line is 7.8, which is set as whole genome level significant threshold. (c) A cartoon indicates physically overlapped deletions. (PDF) [file pgen.1007699.s013.pdf]

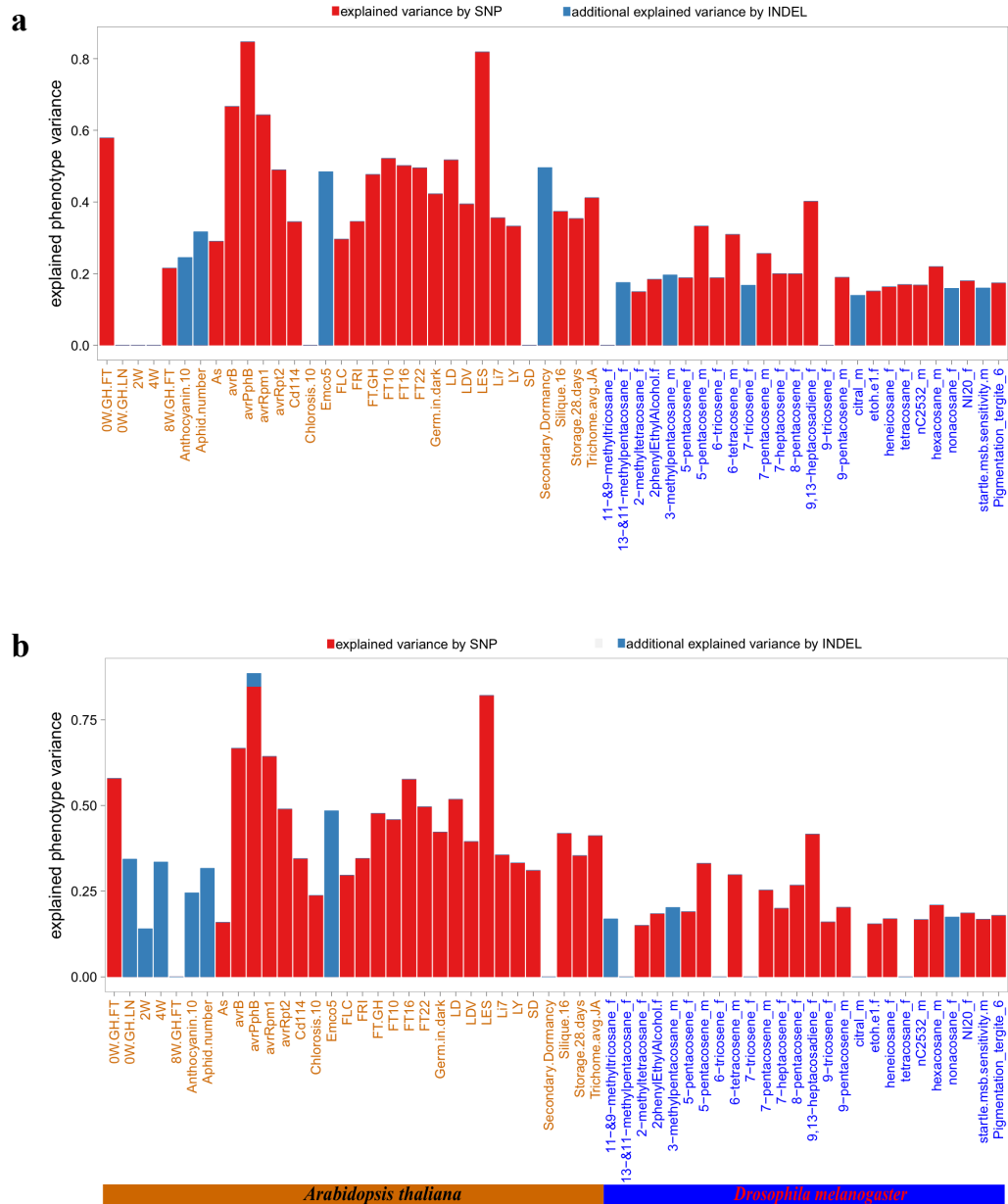

Supplement: S13 Fig — (a) The phenotype variance being explained by SNPs and additional variance explained by INDELs without variants synchronization. (b) The phenotype variance being explained by SNPs and extra variance explained by INDELs after variants synchronization. (c, d) The plot of the ratios of phenotypic variance explained by SNPs and INDELs in GWAS analyses with and without variants synchronization in A. thaliana (c) and D. melanogaster (d). (PDF) [file pgen.1007699.s014.pdf]

quantile-quantile plots of p-values

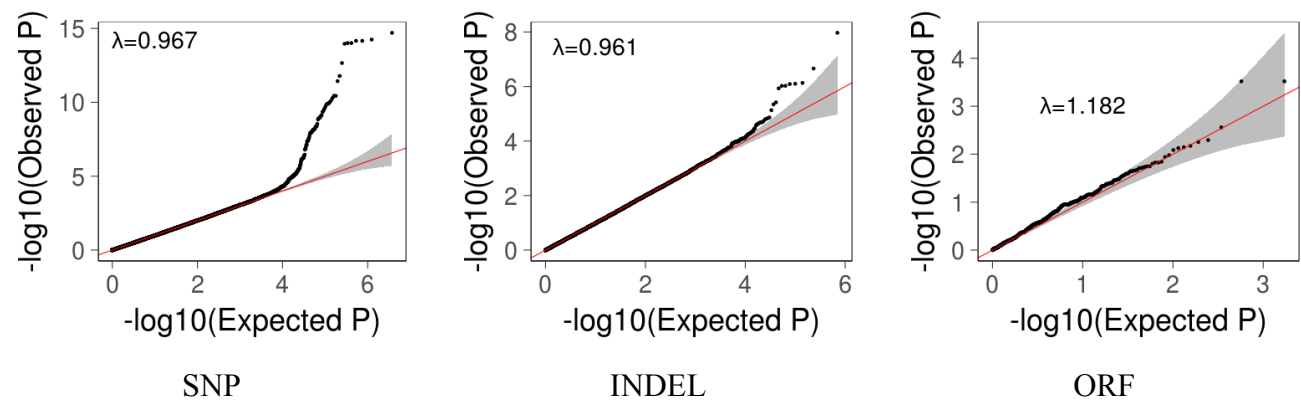

SNP results

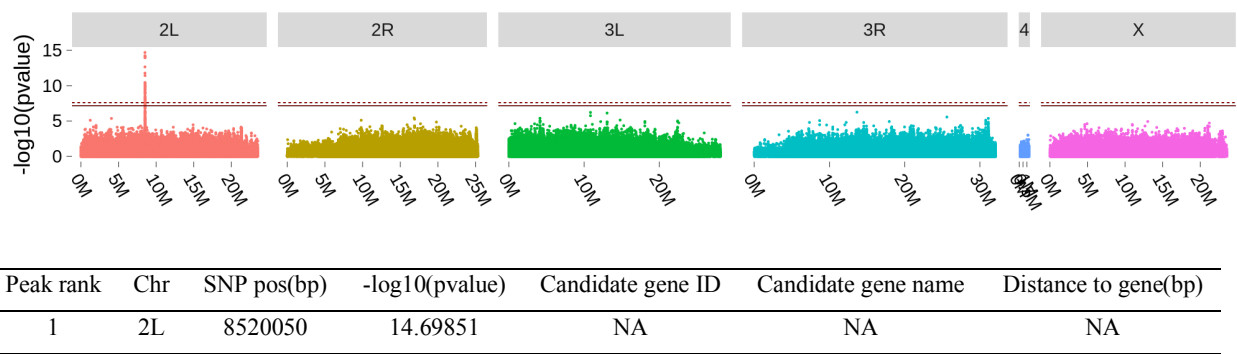

INDEL results

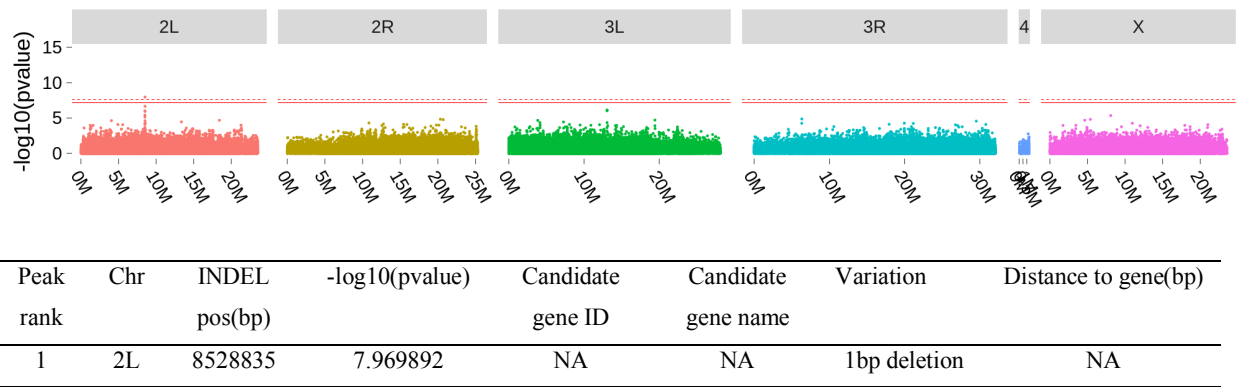

ORFS results

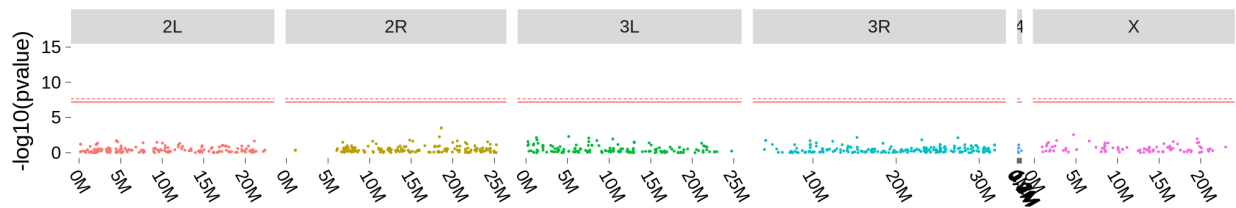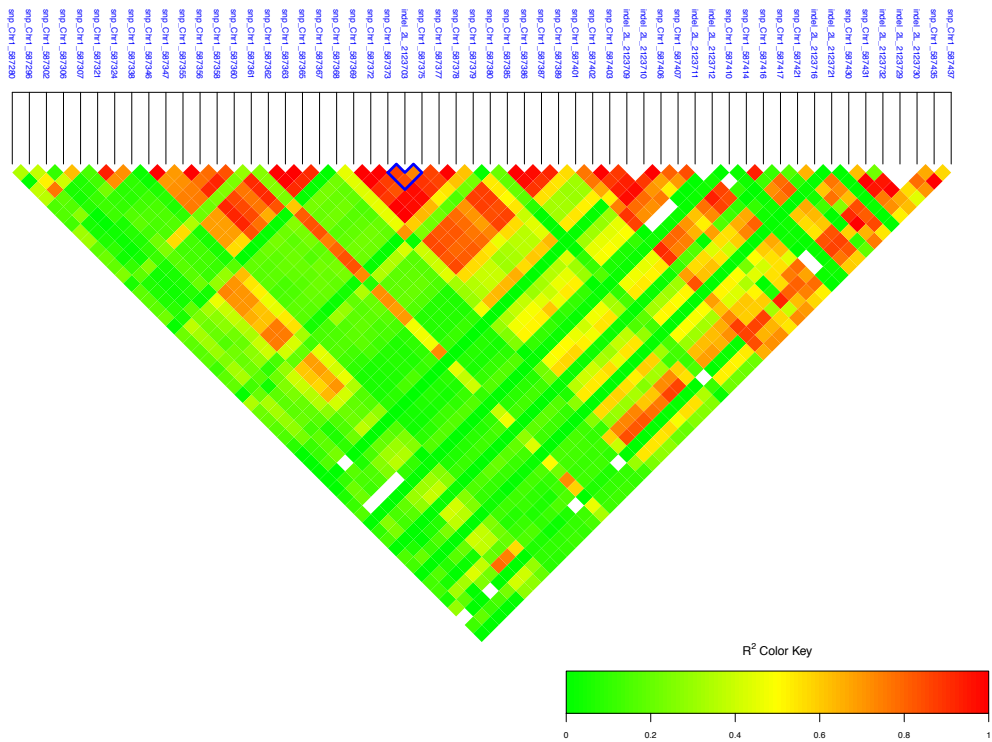

Supplement: S49 Fig — (PDF) [file pgen.1007699.s050.pdf]

quantile-quantile plots of p-values

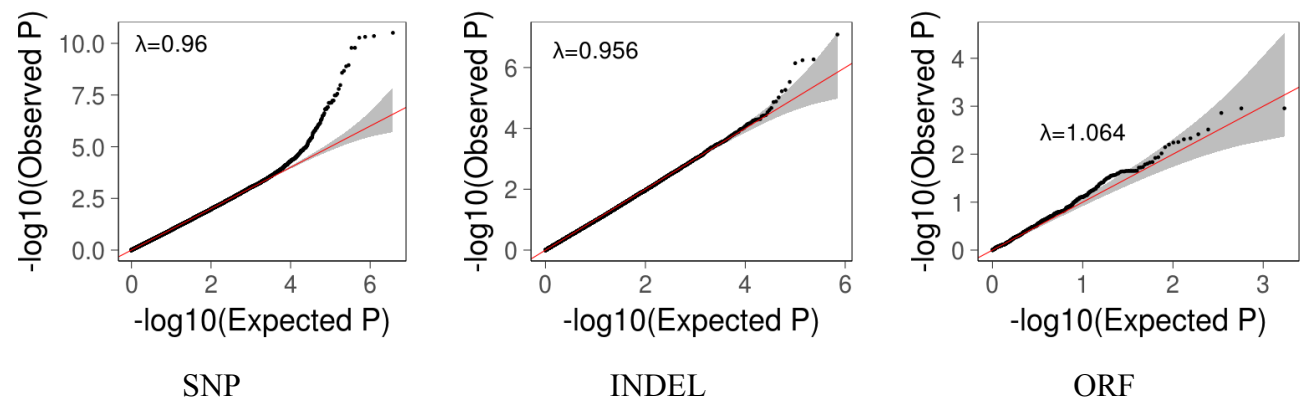

SNP results

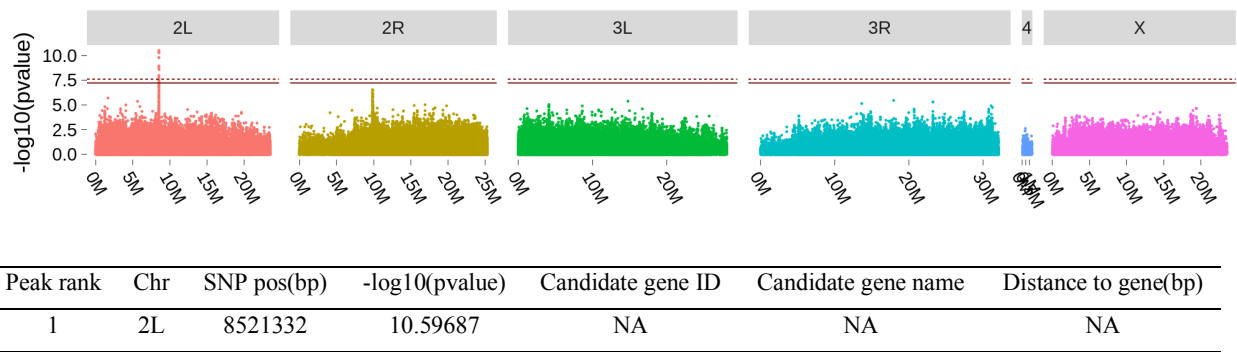

INDEL results

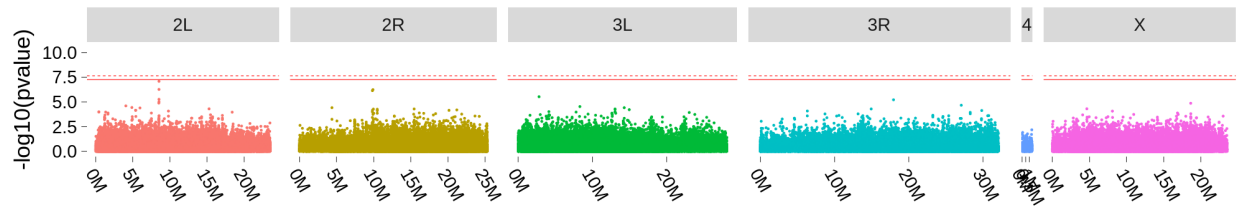

ORFS results

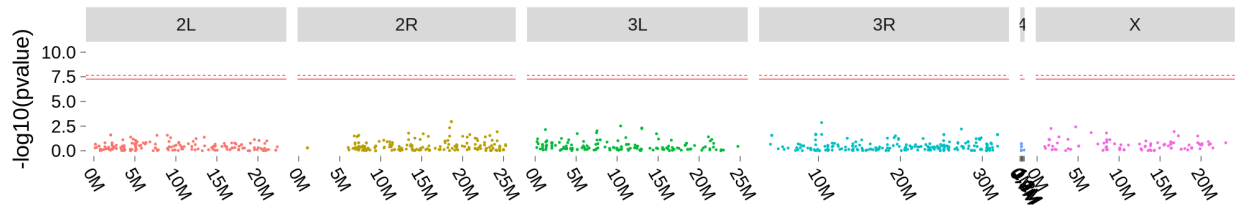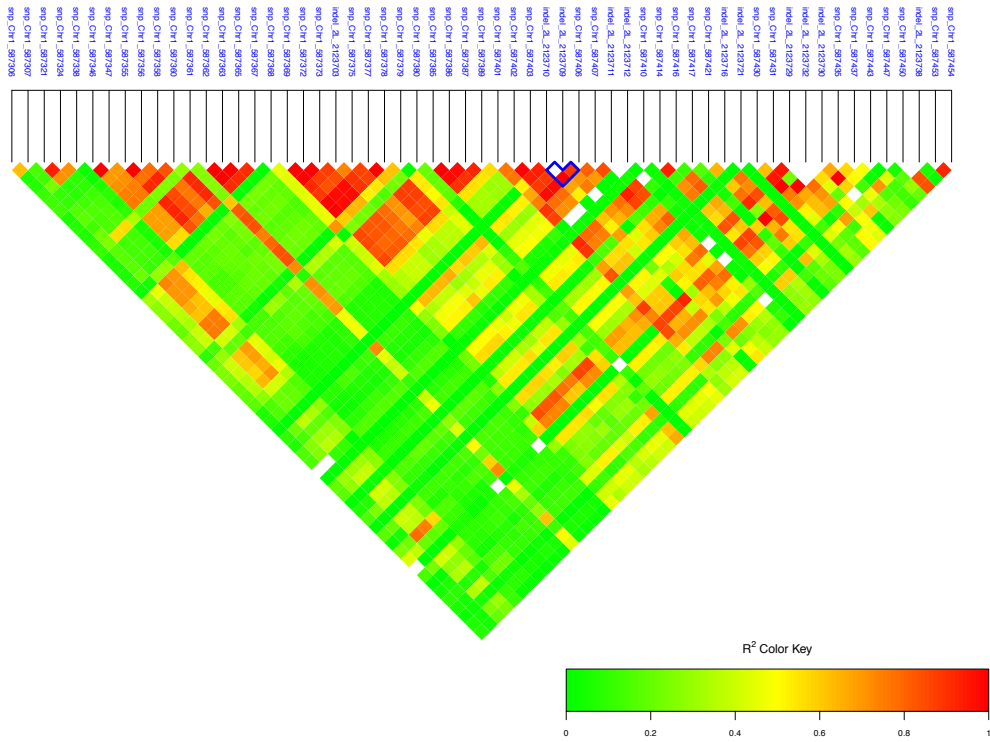

Supplement: S50 Fig — (PDF) [file pgen.1007699.s051.pdf]

quantile-quantile plots of p-values

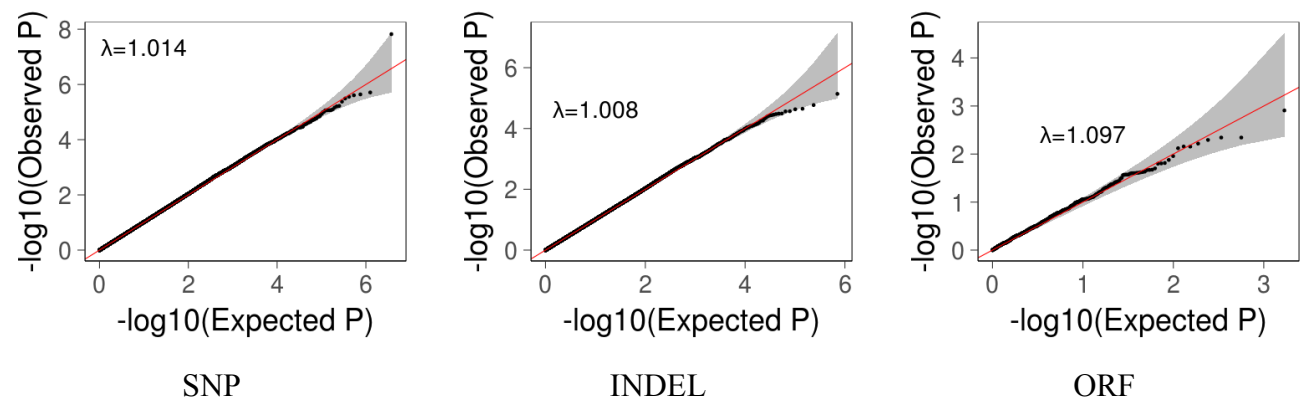

SNP results

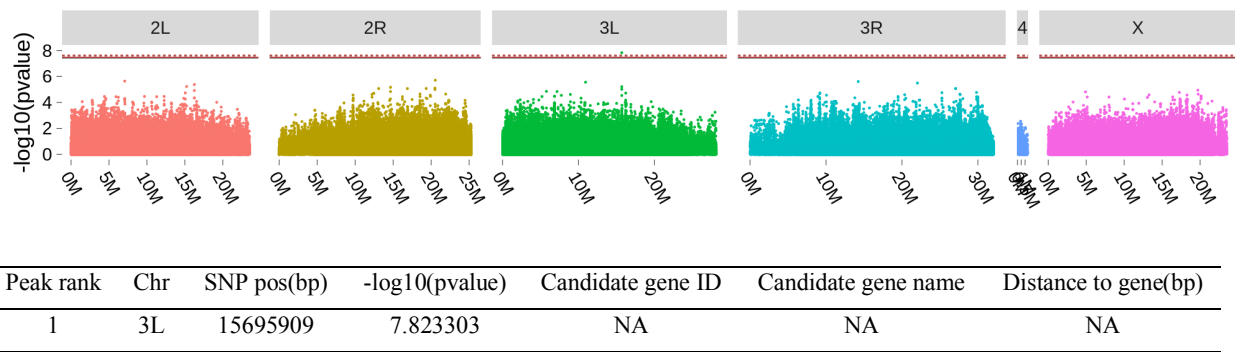

INDEL results

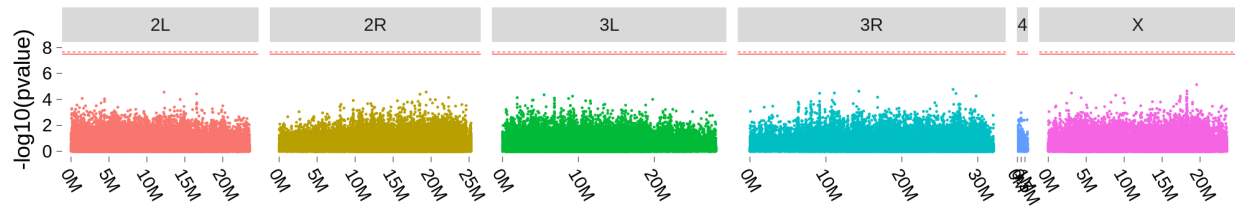

ORFS results

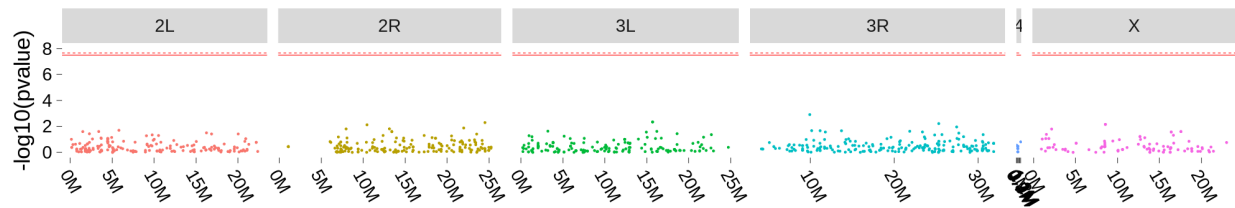

Supplement: S52 Fig — (PDF) [file pgen.1007699.s053.pdf]

quantile-quantile plots of p-values

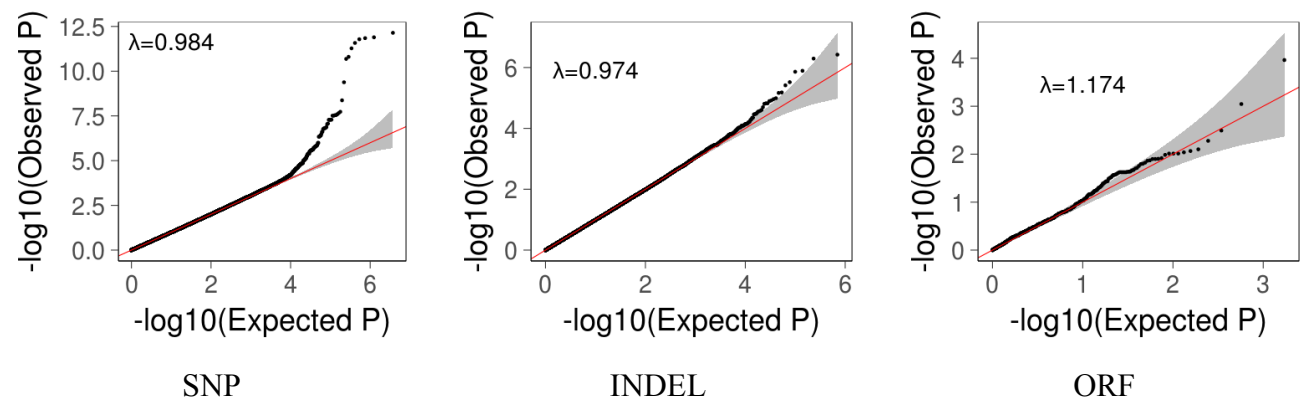

SNP results

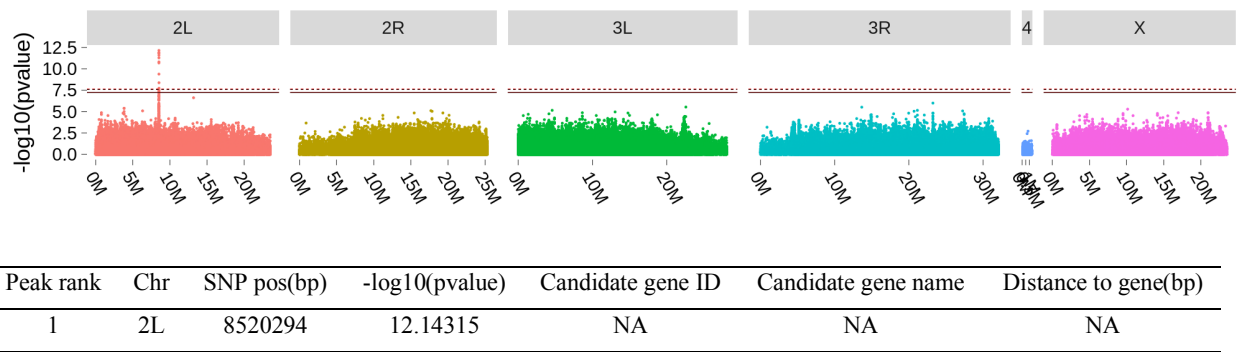

INDEL results

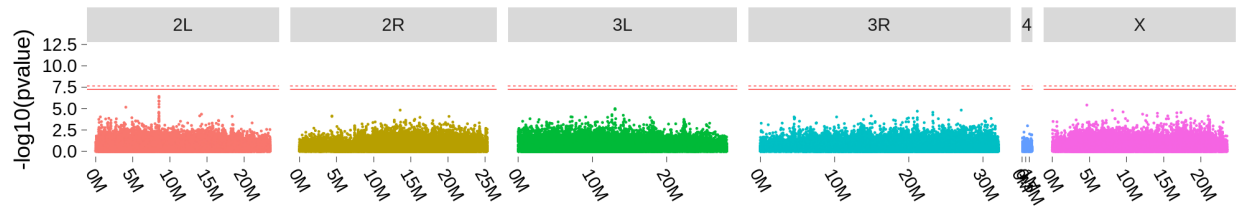

ORFS results

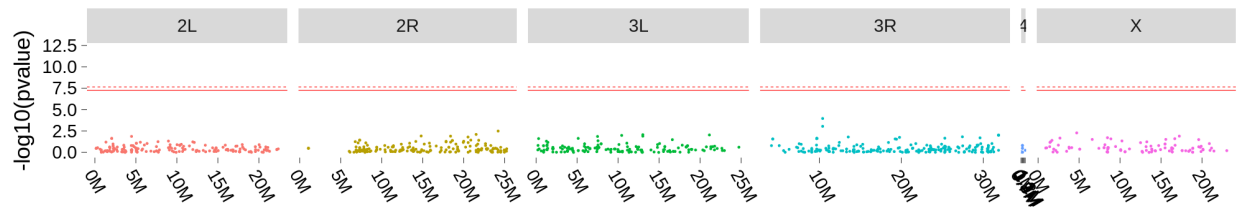

Supplement: S56 Fig — (PDF) [file pgen.1007699.s057.pdf]

quantile-quantile plots of p-values

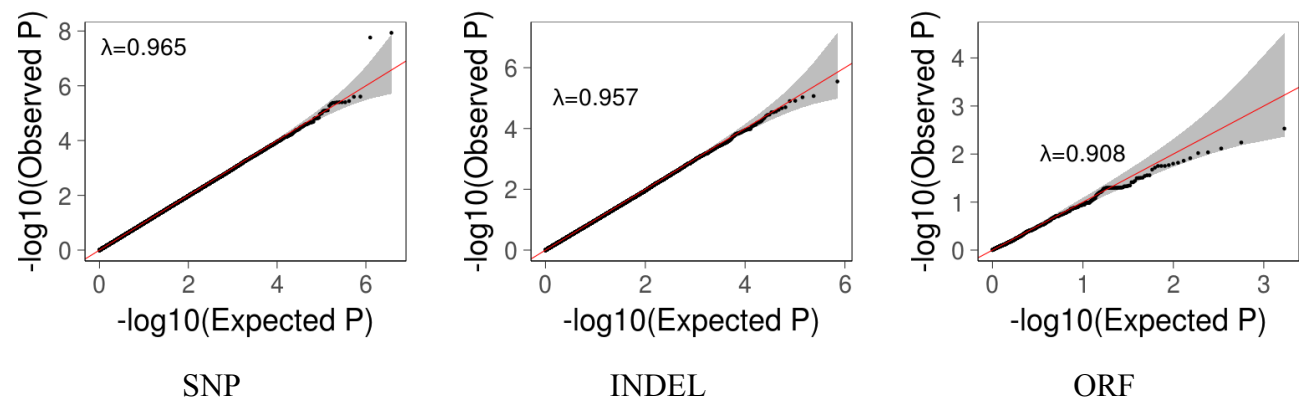

SNP results

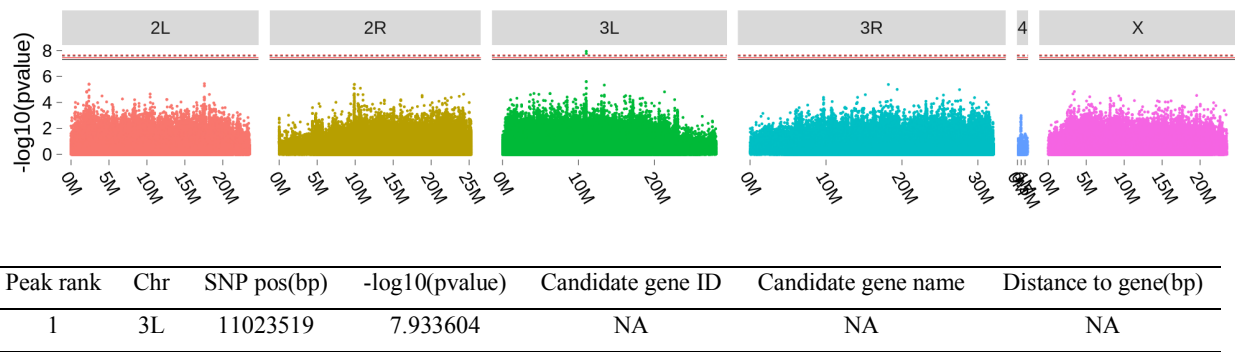

INDEL results

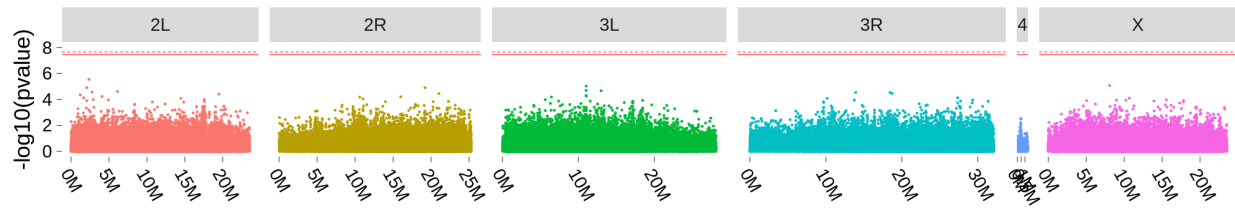

ORFS results

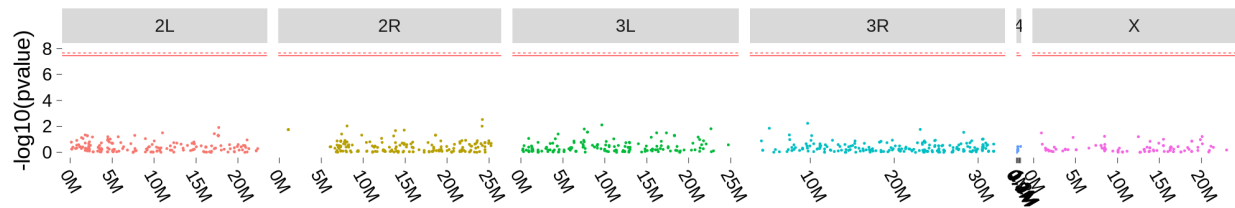

Supplement: S58 Fig — (PDF) [file pgen.1007699.s059.pdf]

quantile-quantile plots of p-values

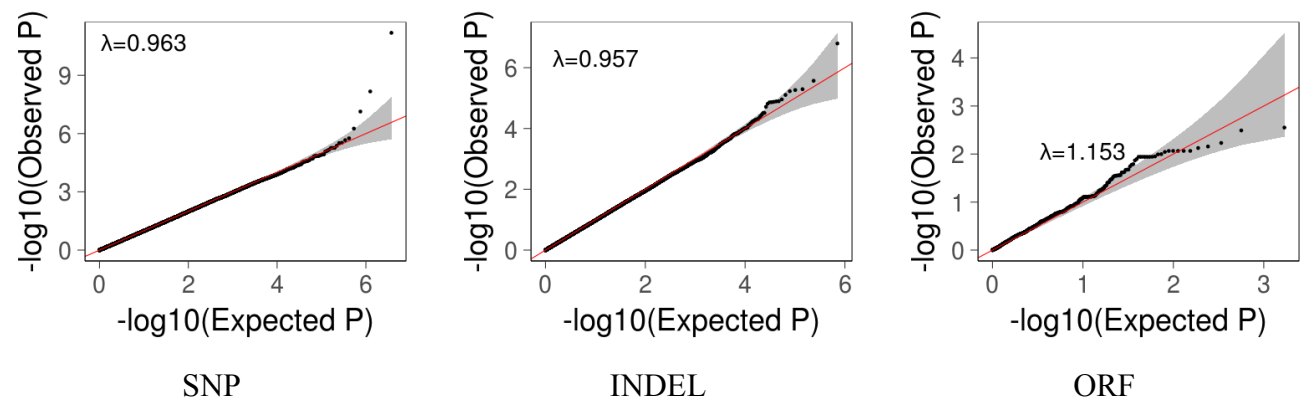

SNP results

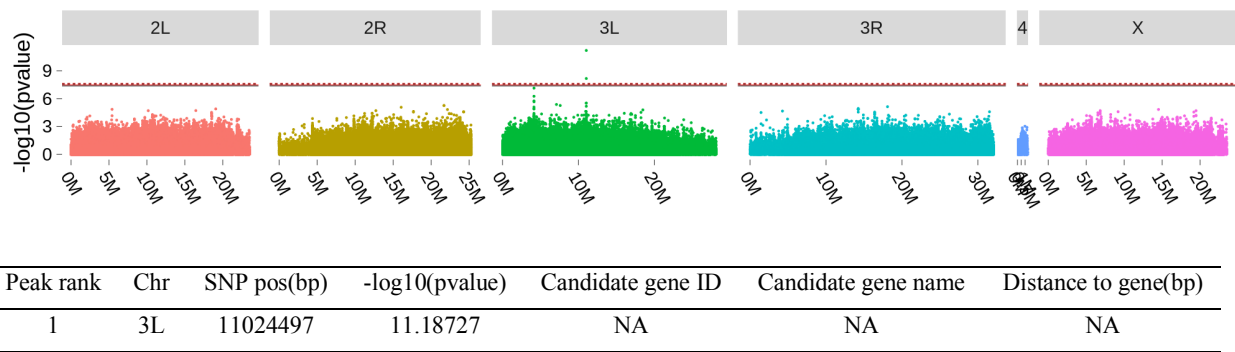

INDEL results

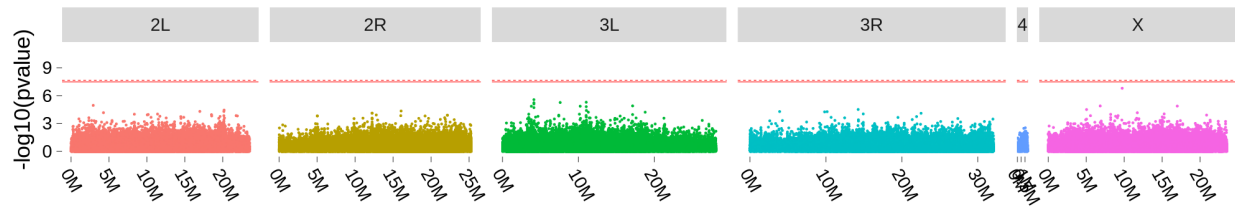

ORFS results

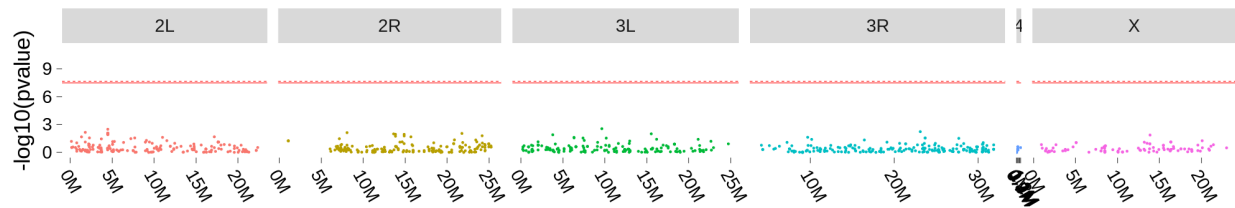

Supplement: S59 Fig — (PDF) [file pgen.1007699.s060.pdf]

quantile-quantile plots of p-values

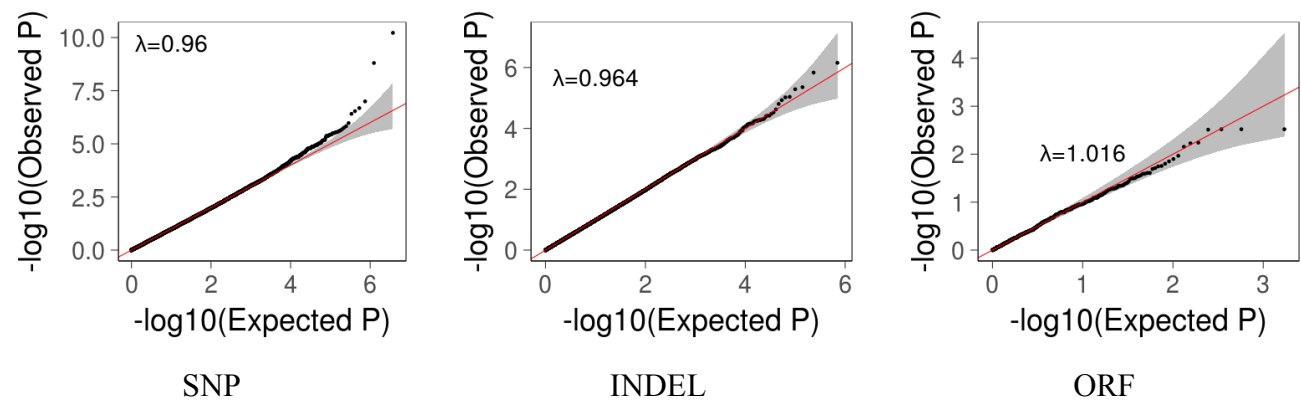

SNP results

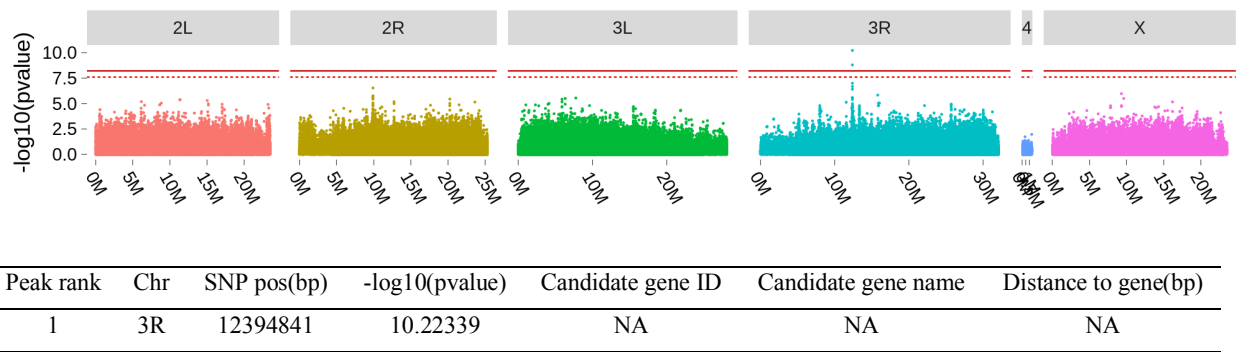

INDEL results

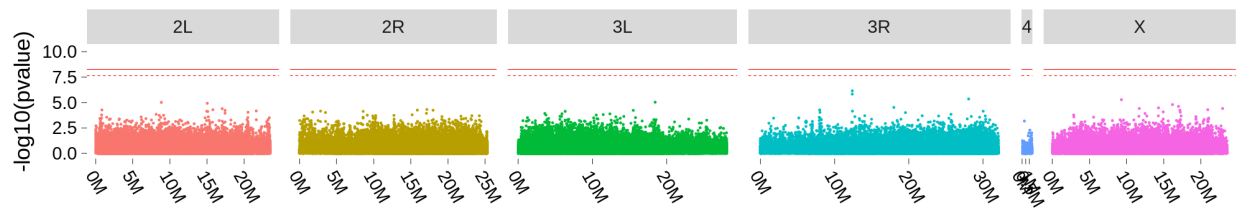

ORFS results

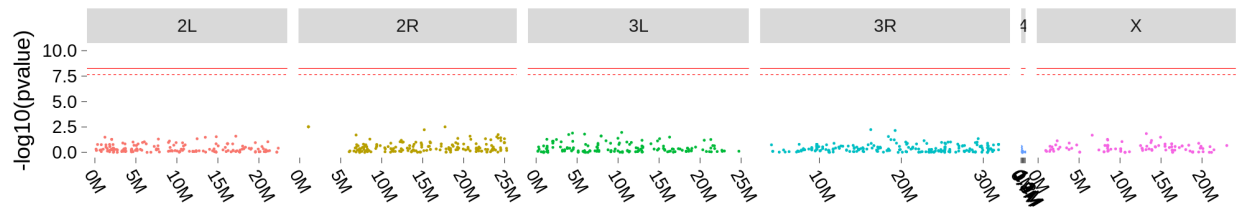

Supplement: S61 Fig — (PDF) [file pgen.1007699.s062.pdf]

quantile-quantile plots of p-values

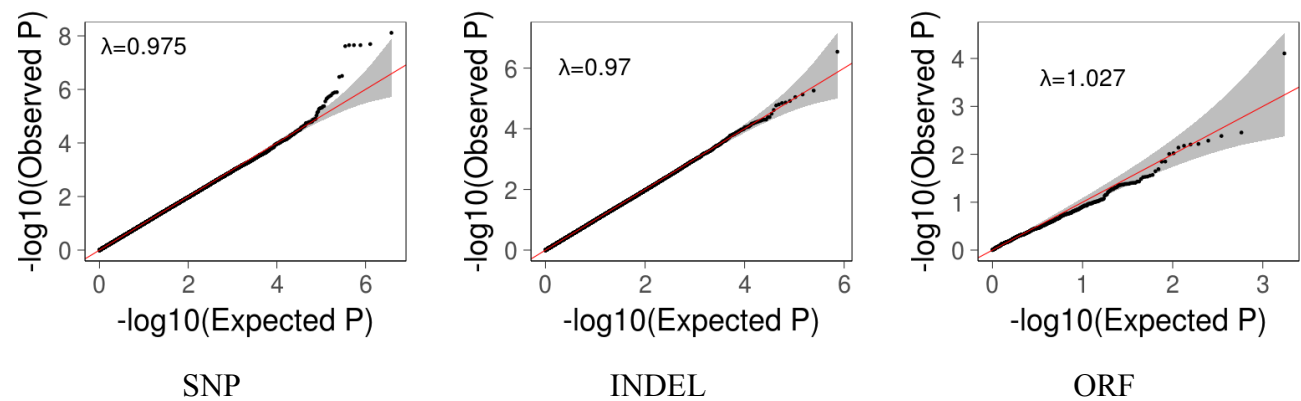

SNP results

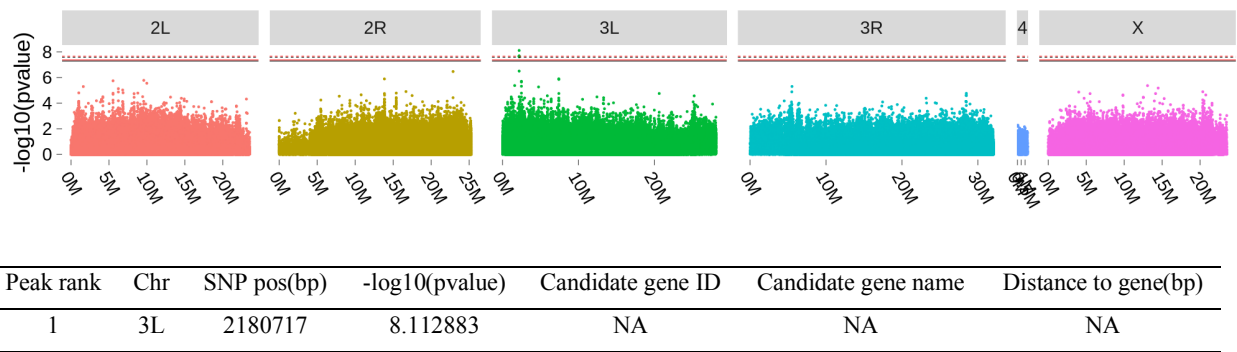

INDEL results

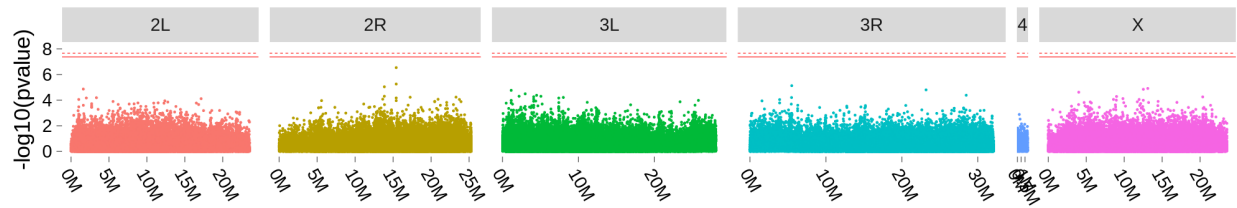

ORFS results

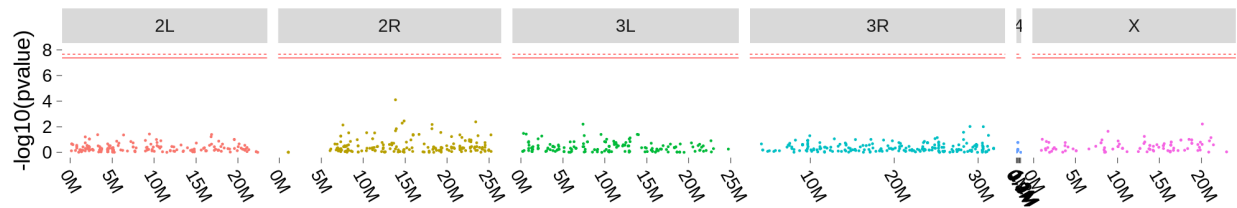

Supplement: S62 Fig — (PDF) [file pgen.1007699.s063.pdf]

quantile-quantile plots of p-values

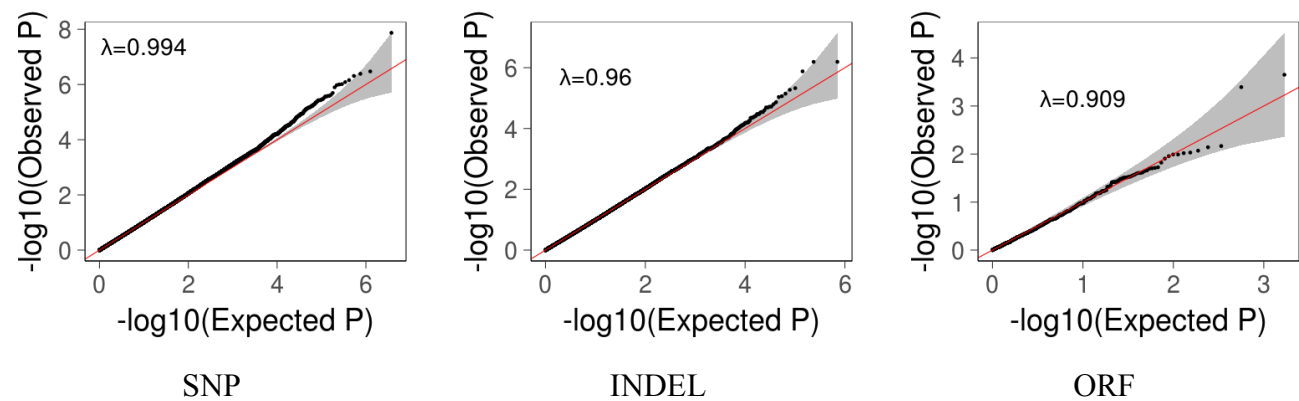

SNP results

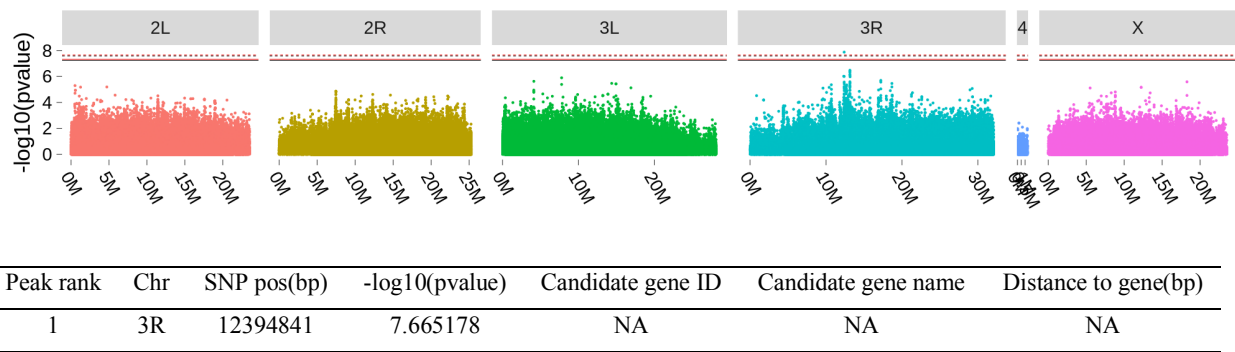

INDEL results

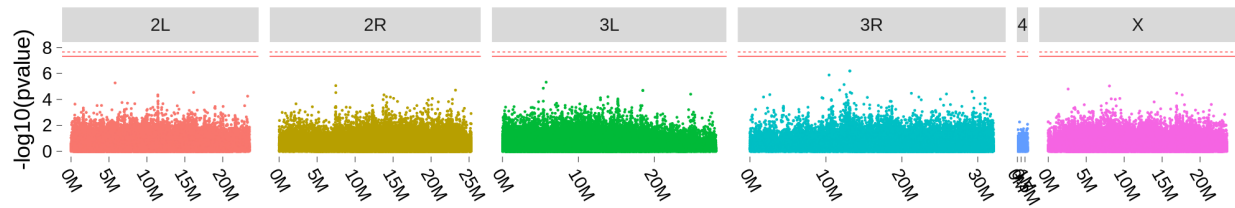

ORFS results

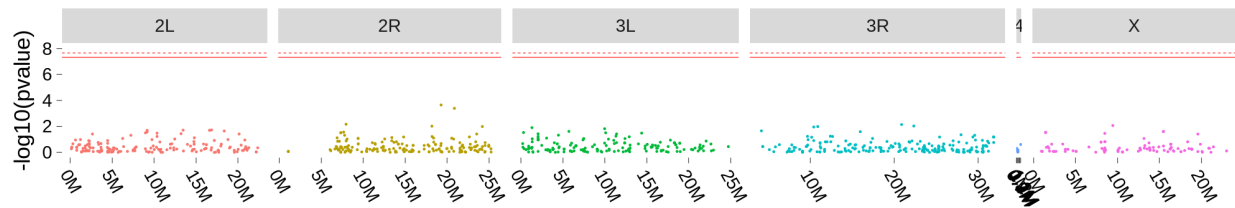

Supplement: S63 Fig — (PDF) [file pgen.1007699.s064.pdf]

quantile-quantile plots of p-values

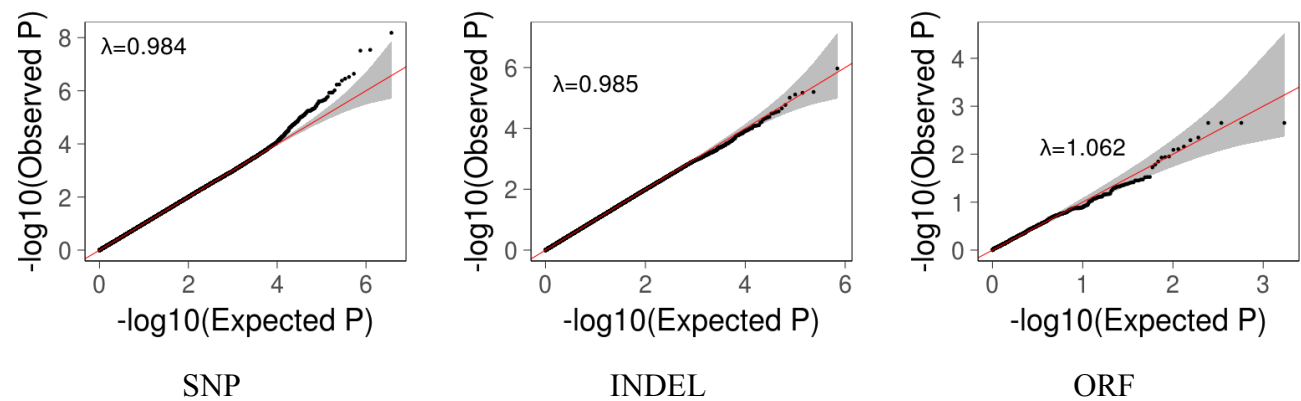

SNP results

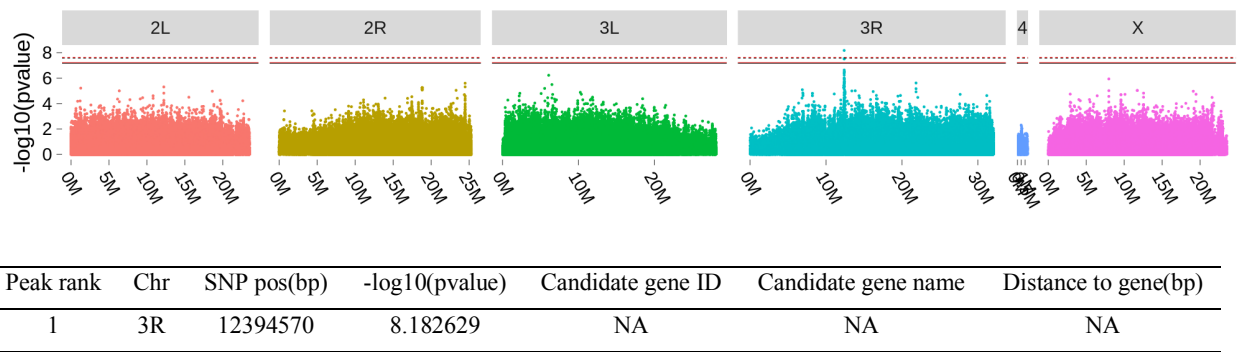

INDEL results

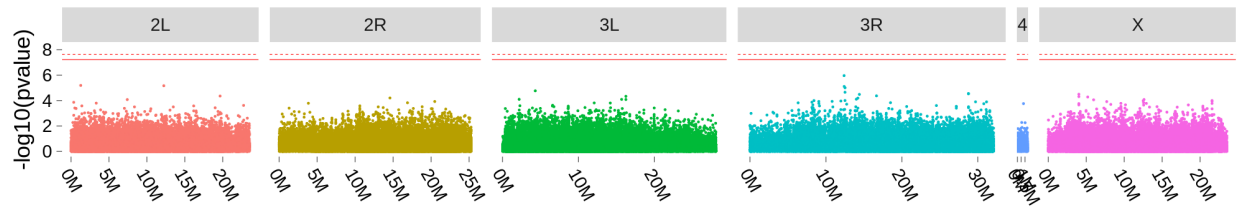

ORFS results

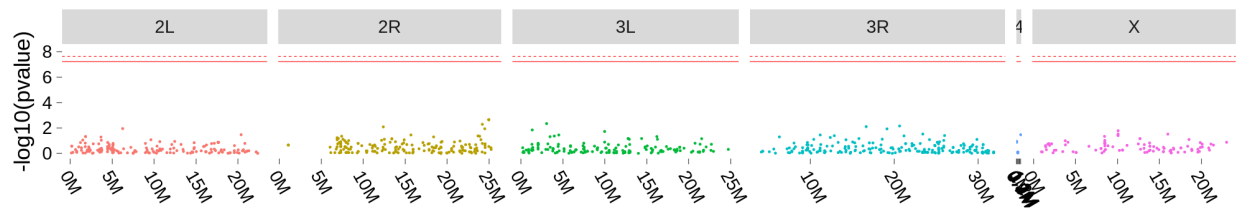

Supplement: S64 Fig — (PDF) [file pgen.1007699.s065.pdf]

quantile-quantile plots of p-values

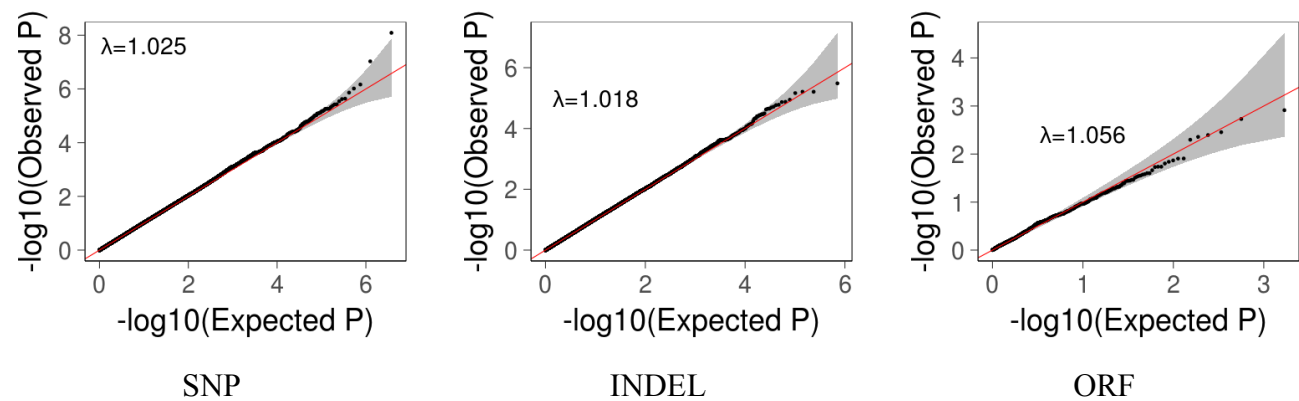

SNP results

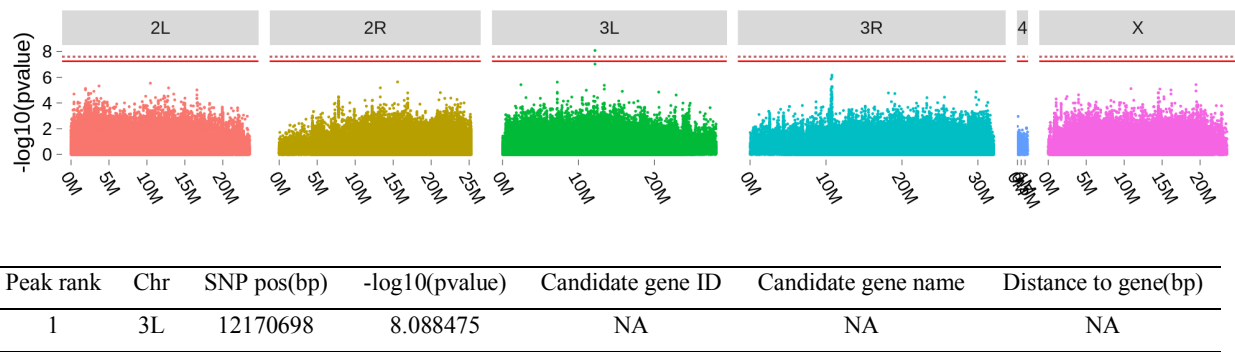

INDEL results

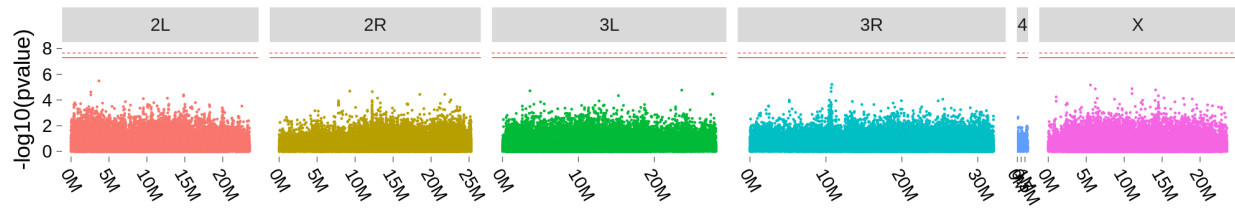

ORFS results

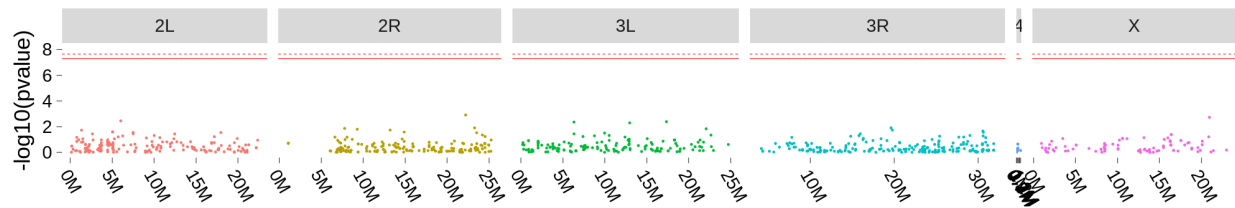

Supplement: S66 Fig — (PDF) [file pgen.1007699.s067.pdf]

quantile-quantile plots of p-values

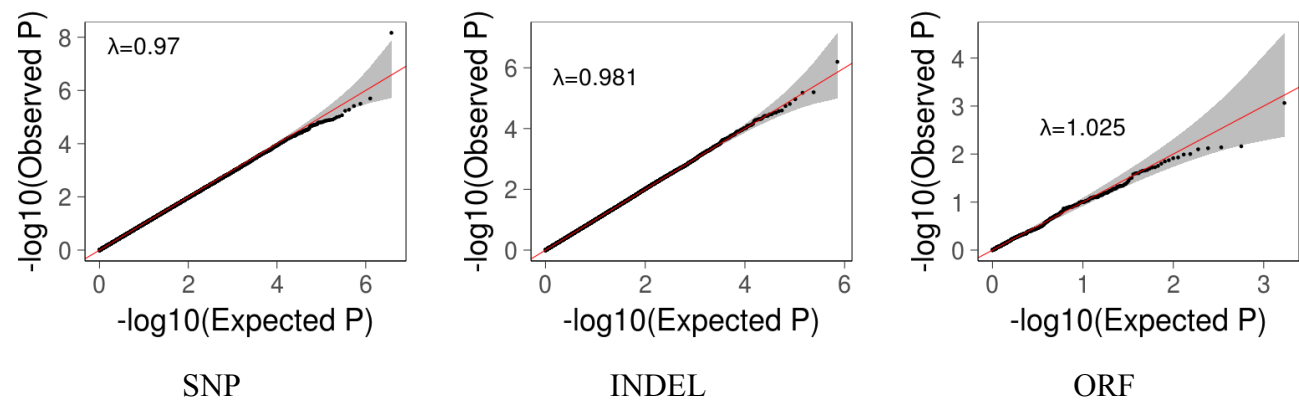

SNP results

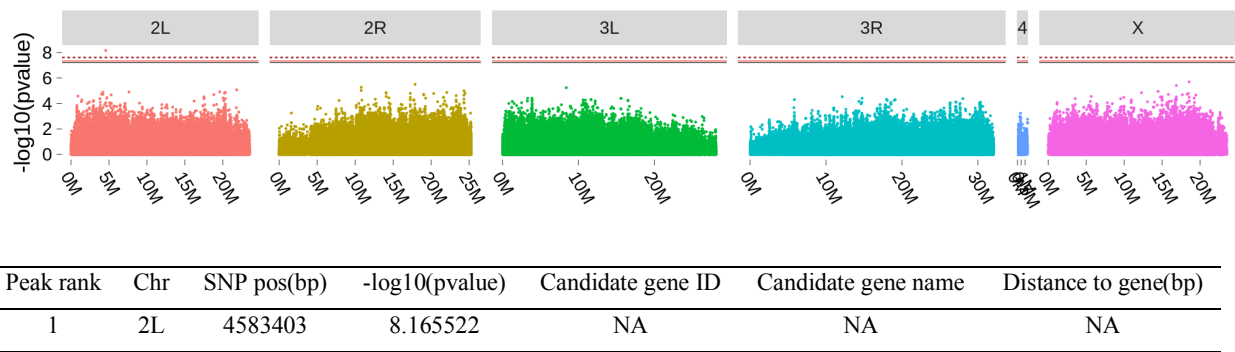

INDEL results

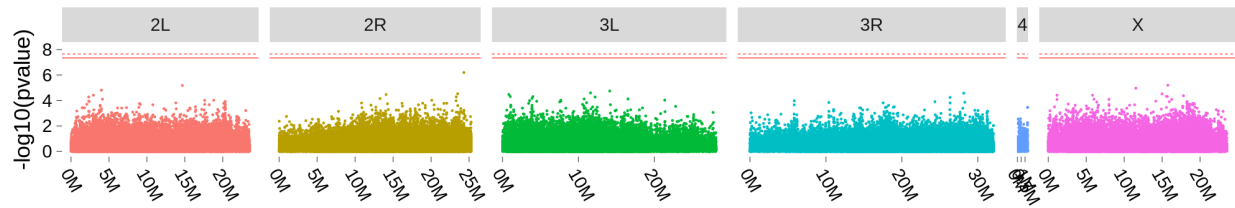

ORFS results

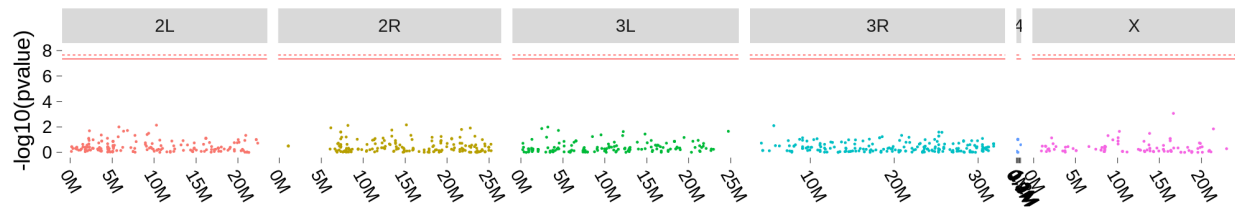

Supplement: S67 Fig — (PDF) [file pgen.1007699.s068.pdf]

quantile-quantile plots of p-values

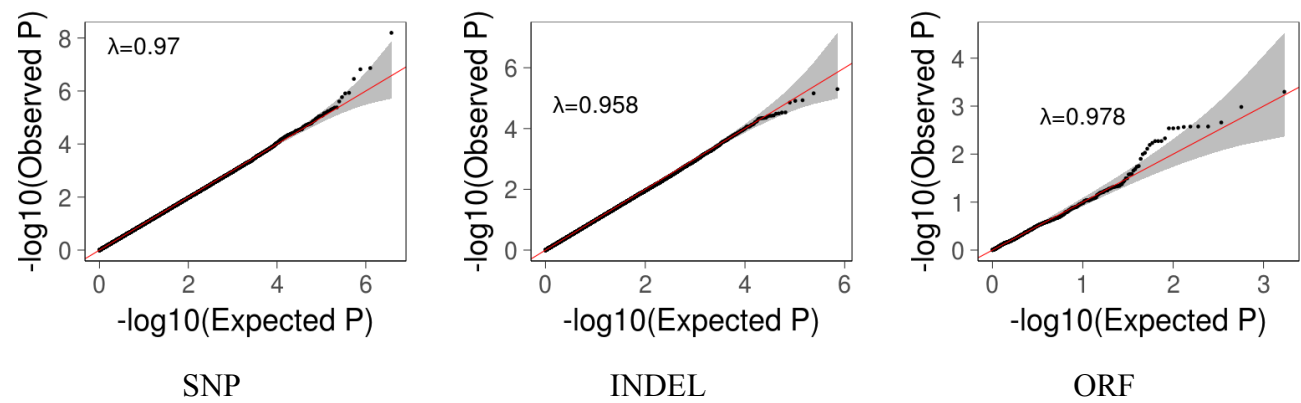

SNP results

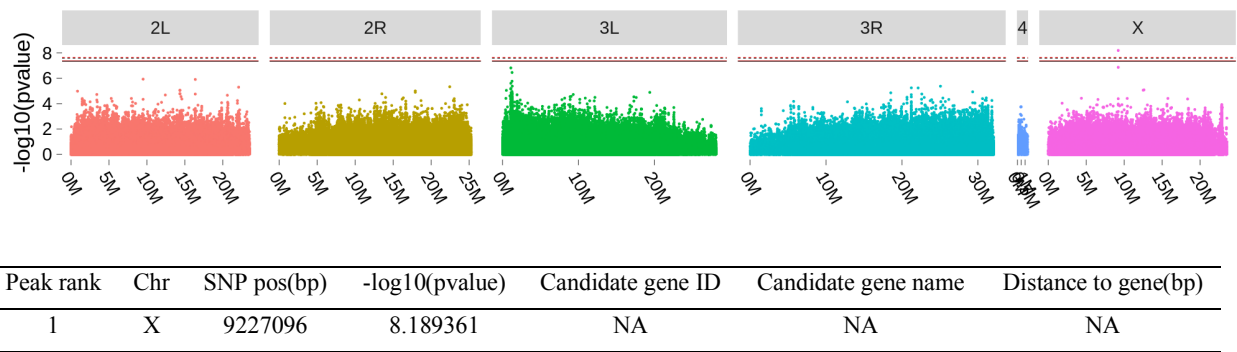

INDEL results

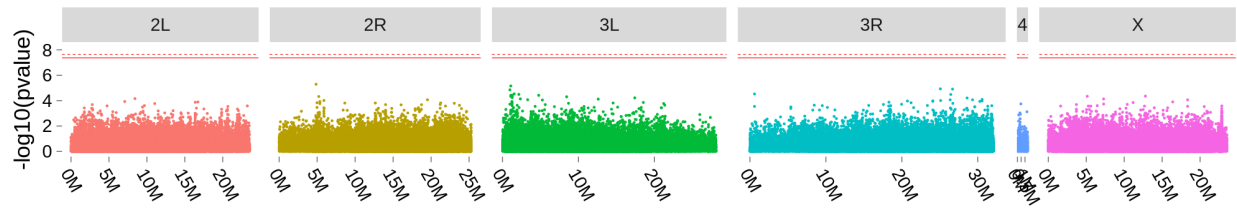

ORFS results

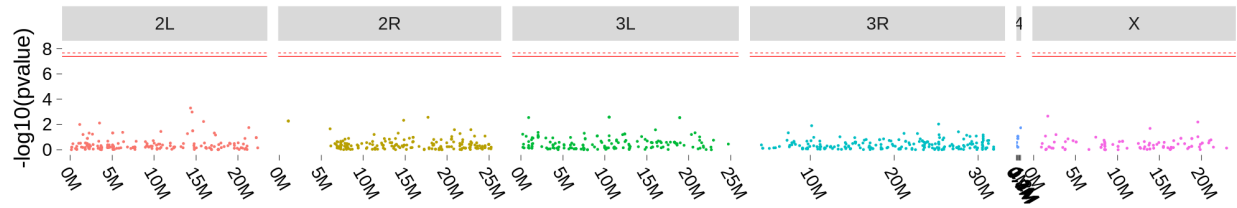

Supplement: S68 Fig — (PDF) [file pgen.1007699.s069.pdf]

quantile-quantile plots of p-values

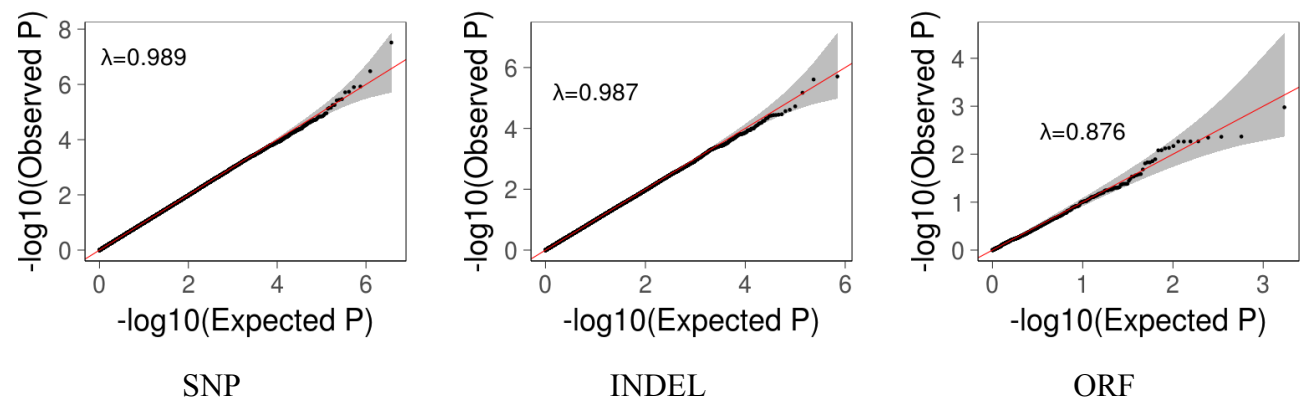

SNP results

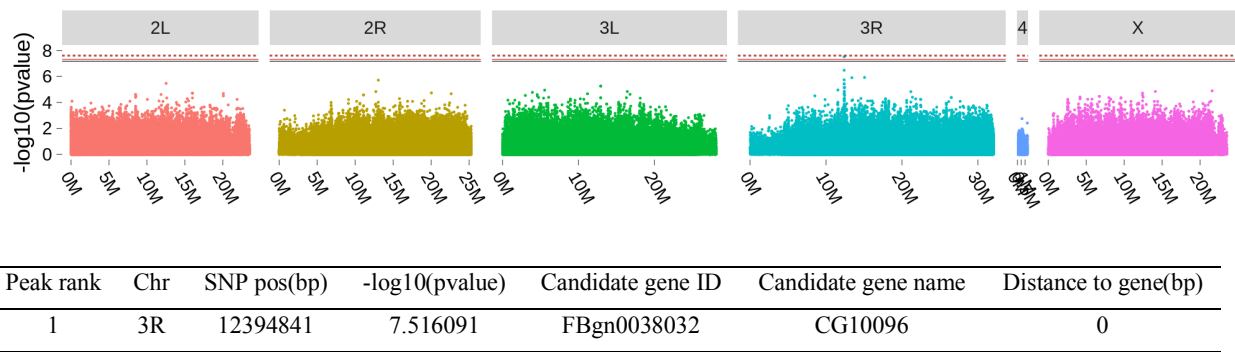

INDEL results

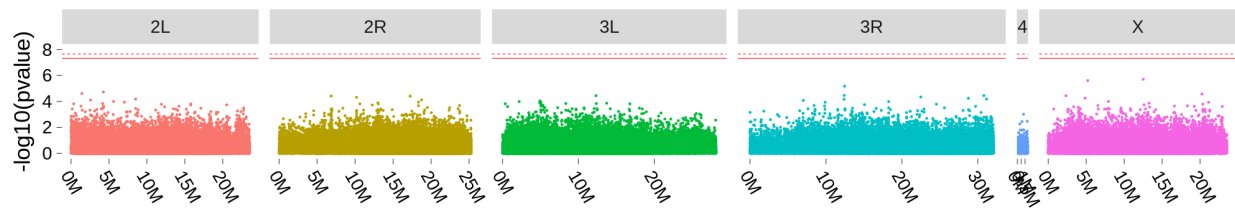

ORFS results

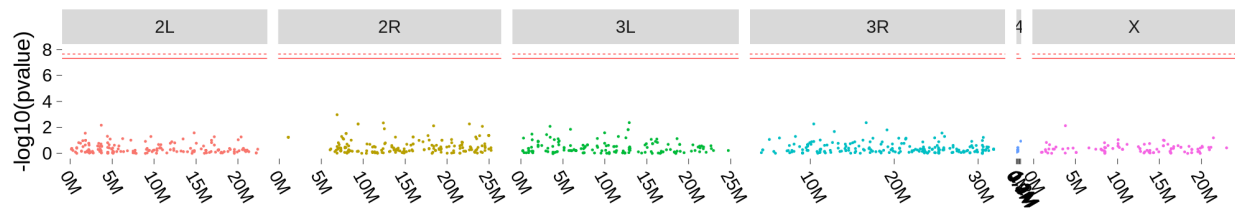

Supplement: S69 Fig — (PDF) [file pgen.1007699.s070.pdf]
