# Supplementary material for: Recovery of novel association loci in Arabidopsis thaliana and Drosophila melanogaster through leveraging INDELs association and integrated burden test
Source: PLoS Genet. 2018 Oct 16;14(10):e1007699. doi: 10.1371/journal.pgen.1007699 (PMC6203403; doi:10.1371/journal.pgen.1007699)

Phenotype histogram and quantile-quantile plots of p-values

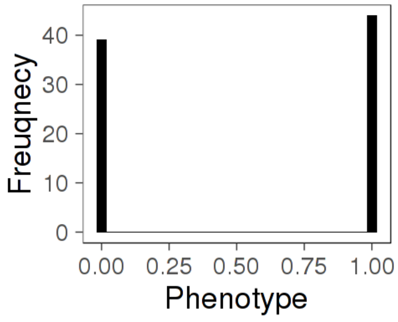


Original phenotype distribution

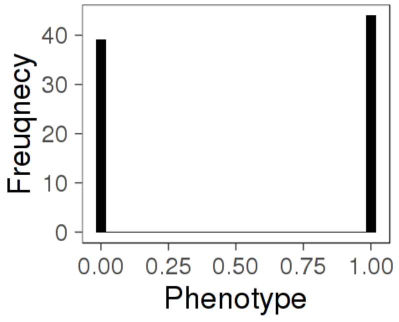


Transformed phenotype distribution

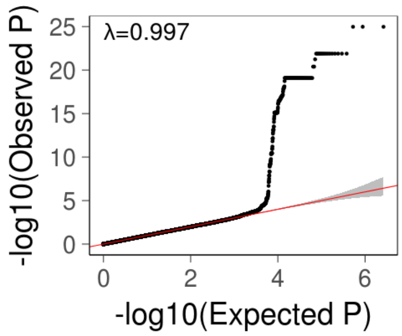


SNP

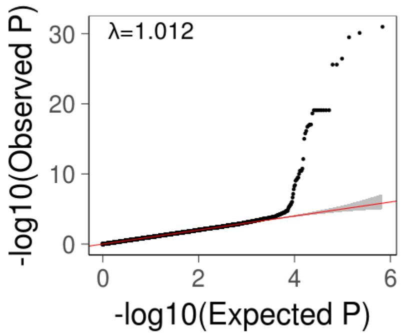


INDEL

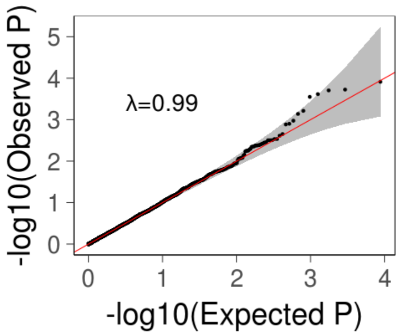


ORF

SNP results

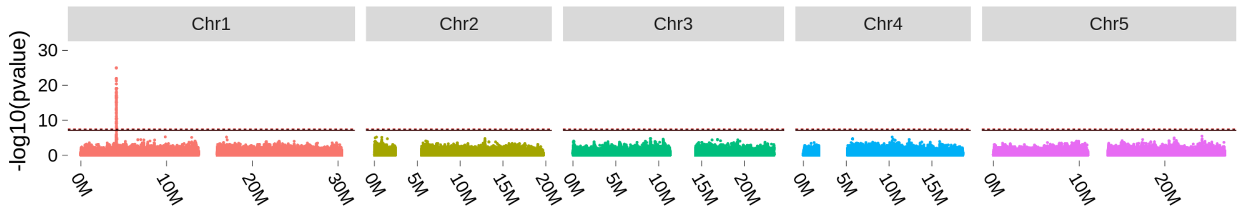


| Peak rank | Chr | SNP pos(bp) | -log10(pvalue) | Candidate gene ID | Candidate gene name | Distance to gene(bp) |
|---|---|---|---|---|---|---|
| 1 | 1 | 4140402 | 24.97581 | AT1G12220 | RPS5 | 4533 |

INDEL results

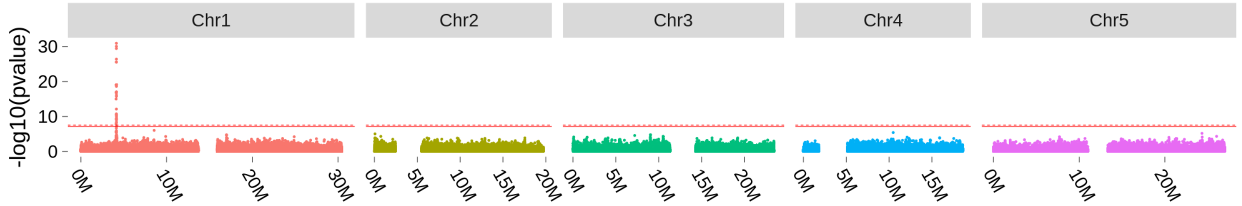


| Peak rank | Chr | INDEL pos(bp) | -log10(pvalue) | Candidate gene ID | Candidate gene name | Variation | Distance to gene(bp) |
|---|---|---|---|---|---|---|---|
| 1 | 1 | 4140250 | 30.98384 | AT1G12220 | RPS5 | -1pb deletion | 4685 |

## ORFS results

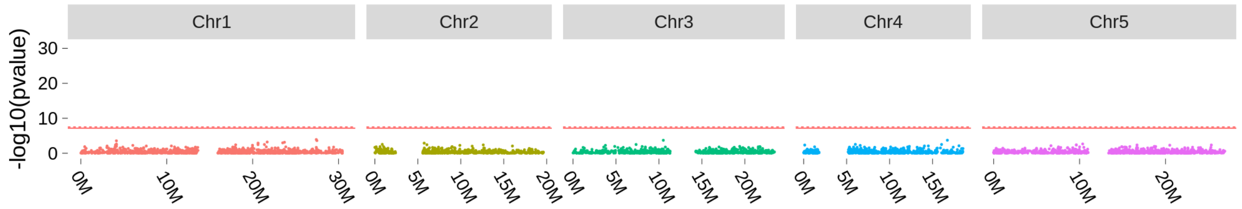


## LD pattern nearby the significant INDEL

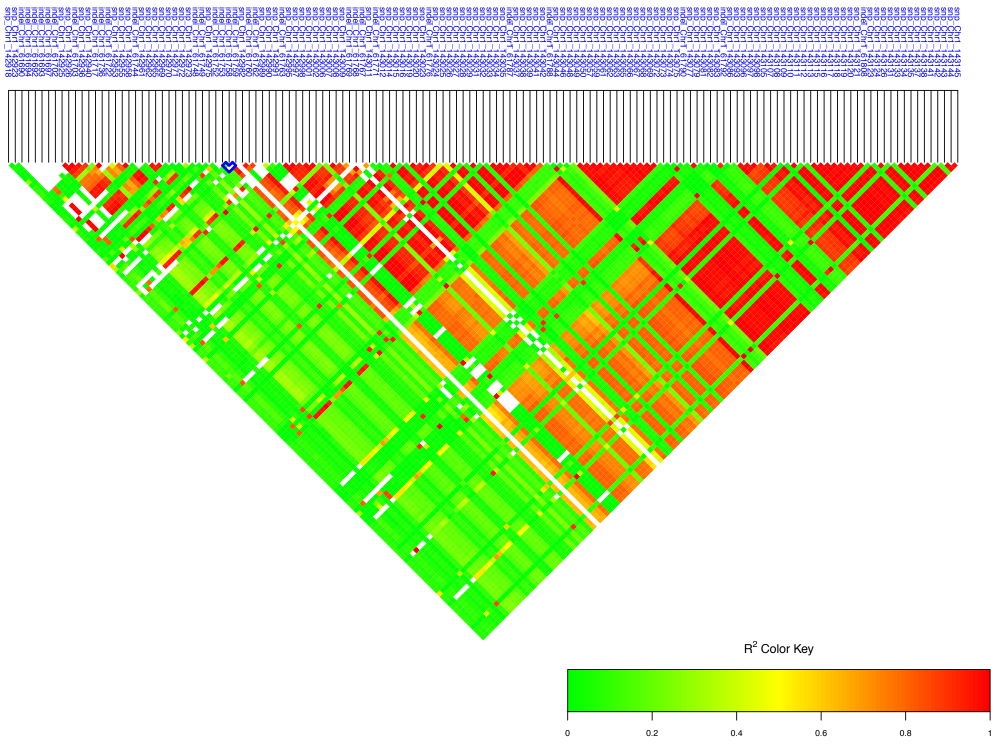

Supplement: S15 Fig — (PDF) [file pgen.1007699.s016.pdf]
