# Supplementary material for: Recovery of novel association loci in Arabidopsis thaliana and Drosophila melanogaster through leveraging INDELs association and integrated burden test
Source: PLoS Genet. 2018 Oct 16;14(10):e1007699. doi: 10.1371/journal.pgen.1007699 (PMC6203403; doi:10.1371/journal.pgen.1007699)

Phenotype histogram and quantile-quantile plots of p-values

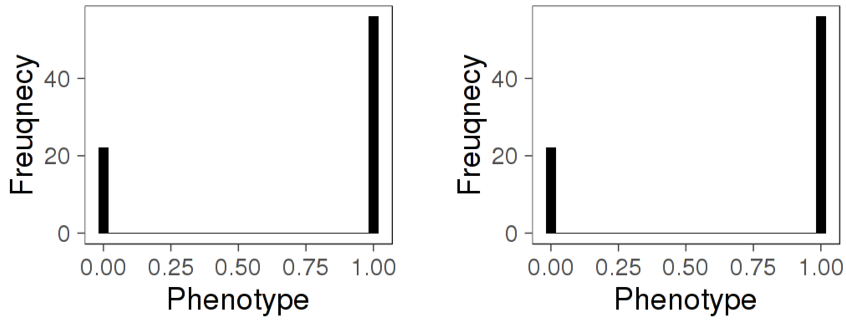

Original phenotype distribution Transformed phenotype distribution

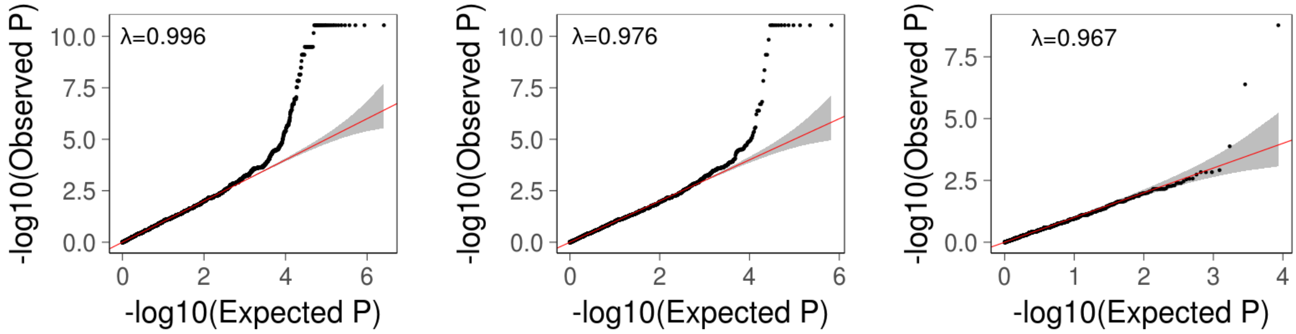

SNP INDEL ORF

SNP results

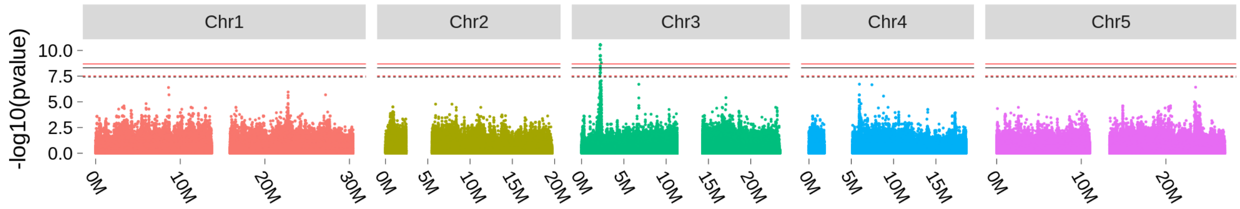

Peak rank	Chr	SNP pos(bp)	-log10(pvalue)	Candidate gene ID	Candidate gene name	Distance to gene(bp)
1	3	2224655	10.53809	AT3G07040	RPM1	1297

INDEL results

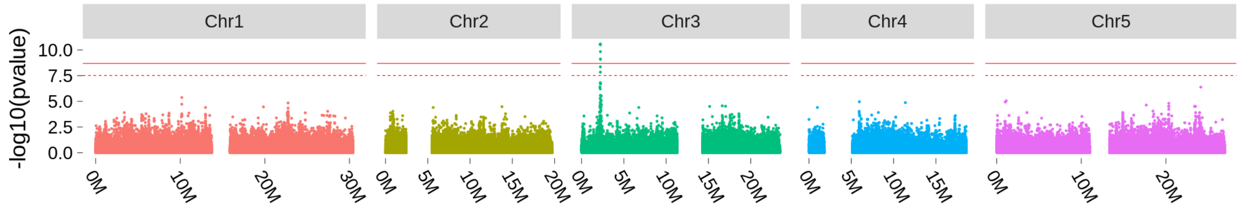

Peak rank	Chr	INDEL pos(bp)	-log10(pvalue)	Candidate gene ID	Candidate gene name	Variation	Distance to gene(bp)
1	3	2224259	10.53809	AT3G07040	RPM1	2bp deletion	1693

ORFS results

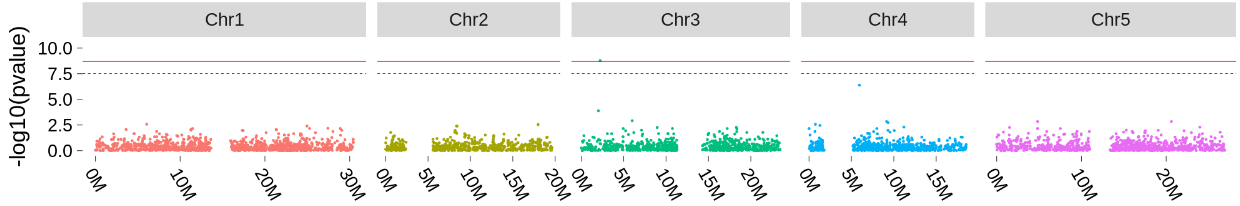

Peak rank	Chr	-log10(pvalue)	Candidate gene ID	Candidate gene name
1	3	8.775945	AT3G07040	RPM1

LD pattern nearby the significant INDEL

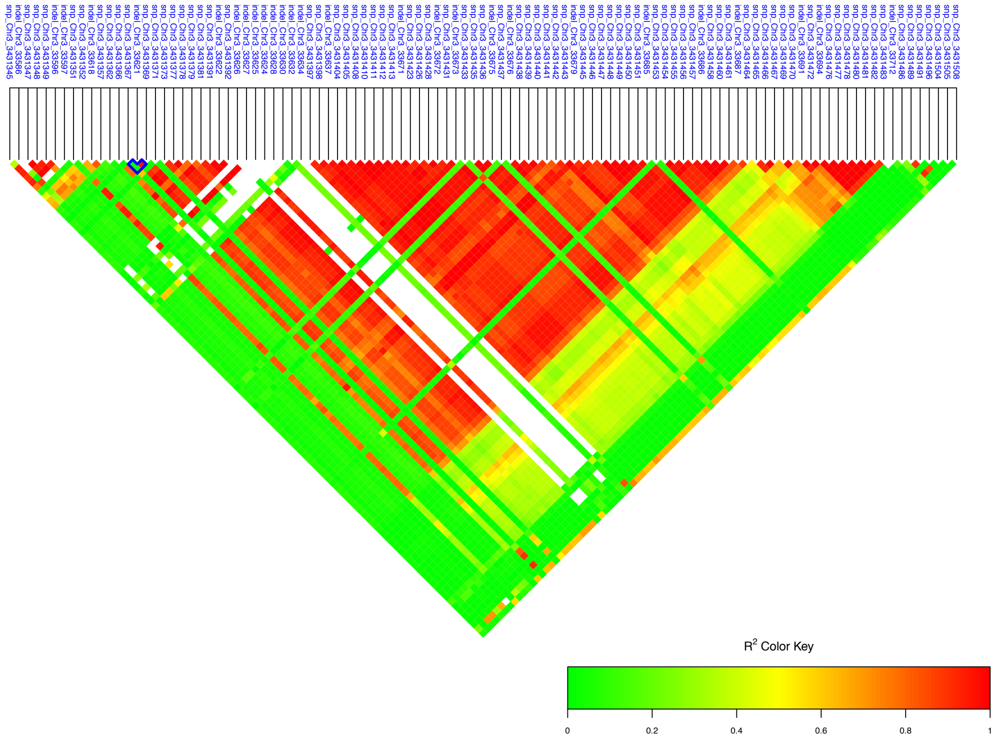

Supplement: S16 Fig — (PDF) [file pgen.1007699.s017.pdf]
