# Supplementary material for: Recovery of novel association loci in Arabidopsis thaliana and Drosophila melanogaster through leveraging INDELs association and integrated burden test
Source: PLoS Genet. 2018 Oct 16;14(10):e1007699. doi: 10.1371/journal.pgen.1007699 (PMC6203403; doi:10.1371/journal.pgen.1007699)
Supplement: S20 Fig — (PDF) [file pgen.1007699.s021.pdf]

Phenotype histogram and quantile-quantile plots of p-values

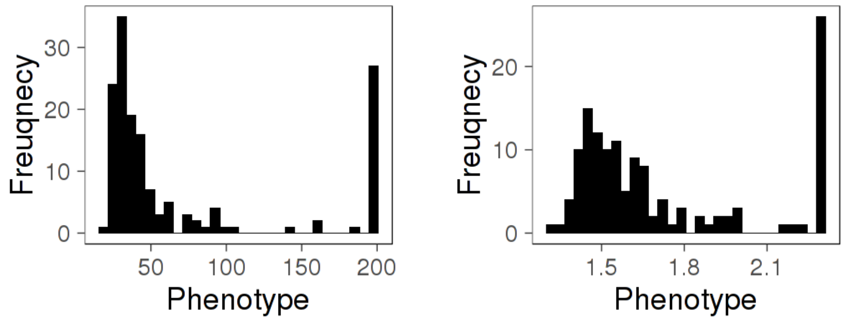


Original phenotype distribution Transformed phenotype distribution

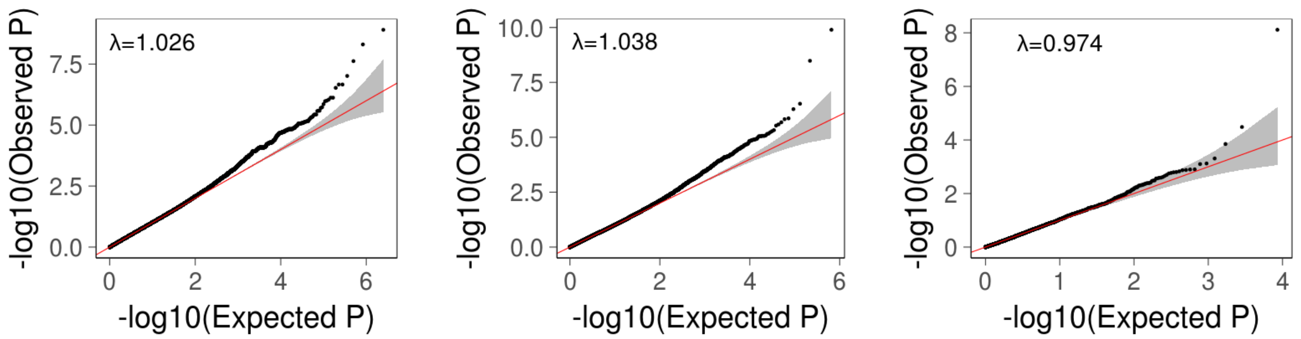


SNP INDEL ORF

SNP results

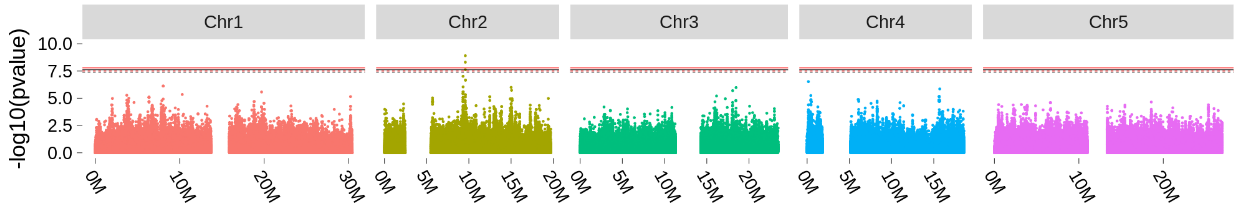


| Peak rank | Chr | SNP pos(bp) | -log10(pvalue) | Candidate gene ID | Candidate gene name | Distance to gene(bp) |
|---|---|---|---|---|---|---|
| 1 | 2 | 9581436 | 8.906688 | AT2G22540 | SVP | 0 |

INDEL results

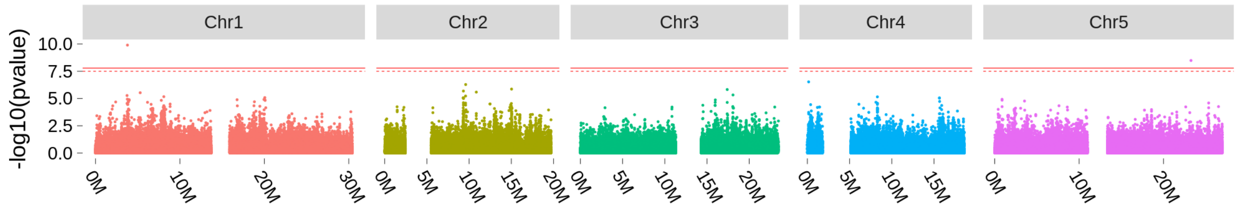

| Peak rank | Chr | INDEL pos(bp) | -log10(pvalue) | Candidate gene ID | Candidate gene name | Variation | Distance to gene(bp) |
|---|---|---|---|---|---|---|---|
| 1 | 1 | 3785721 | 9.89584 | NA | NA | 3bp deletion | NA |
| 2 | 5 | 23249423 | 8.483741 | AT5G57380 | VIN3 | 4bp deletion | 0 |

## ORFS results

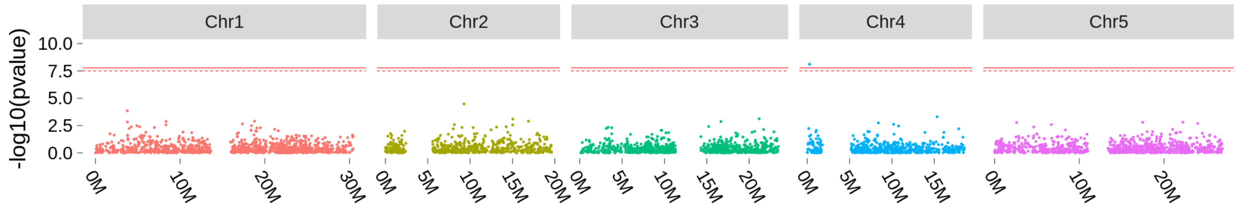


| Peak rank | Chr | -log10(pvalue) | Candidate gene ID | Candidate gene name |
|---|---|---|---|---|
| 1 | 4 | 8.114638 | AT4G00650 | FRI |
