# Supplementary material for: Recovery of novel association loci in Arabidopsis thaliana and Drosophila melanogaster through leveraging INDELs association and integrated burden test
Source: PLoS Genet. 2018 Oct 16;14(10):e1007699. doi: 10.1371/journal.pgen.1007699 (PMC6203403; doi:10.1371/journal.pgen.1007699)
Supplement: S28 Fig — (PDF) [file pgen.1007699.s029.pdf]

Phenotype histogram and quantile-quantile plots of p-values

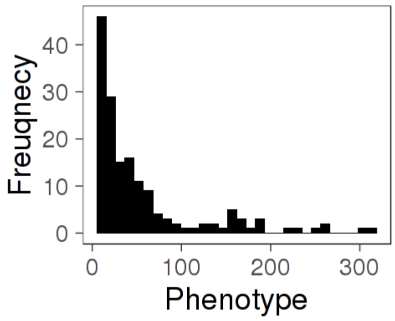

Original phenotype distribution

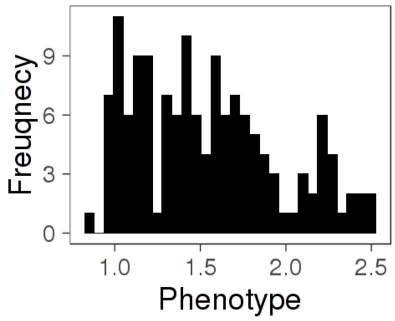

Transformed phenotype distribution

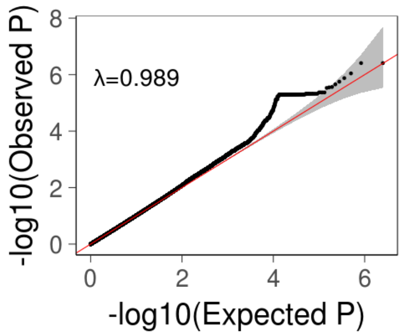

SNP

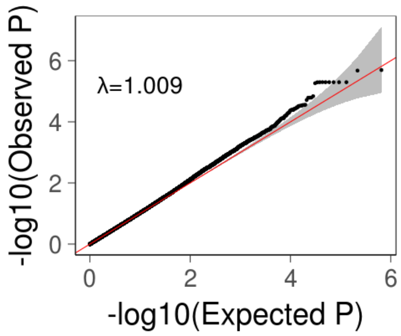

INDEL

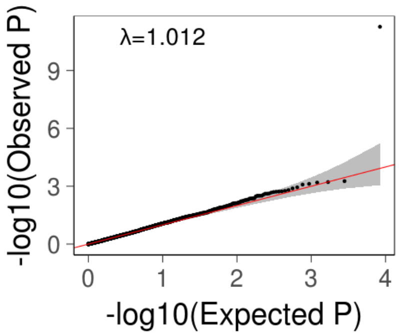

ORF

SNP results

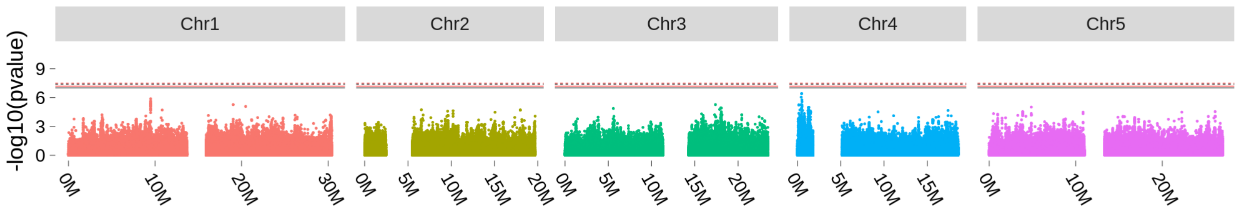

INDEL results

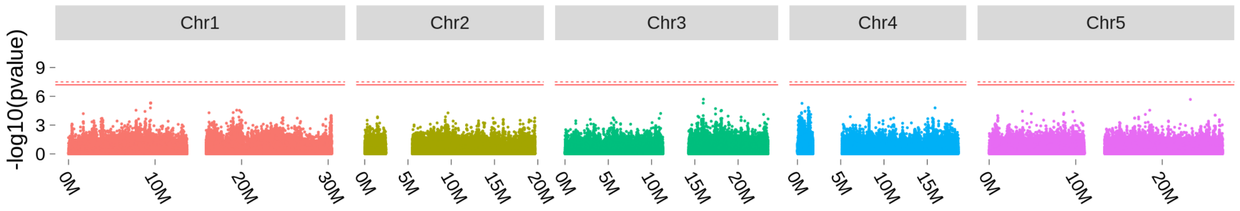

ORFS results

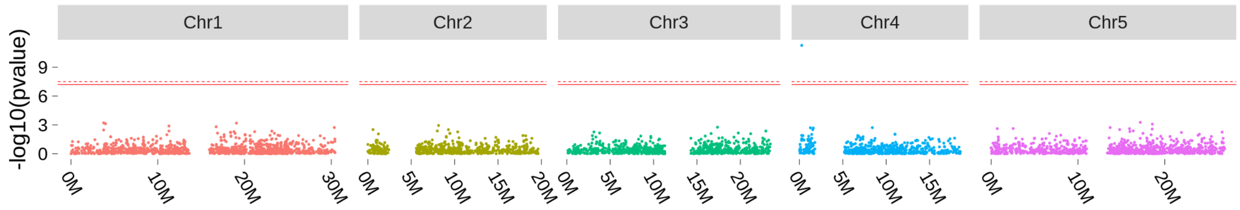

Peak rank	Chr	-log10(pvalue)	Candidate gene ID	Candidate gene name
1	4	11.26632	AT4G00650	FRI
