# Supplementary material for: Recovery of novel association loci in Arabidopsis thaliana and Drosophila melanogaster through leveraging INDELs association and integrated burden test
Source: PLoS Genet. 2018 Oct 16;14(10):e1007699. doi: 10.1371/journal.pgen.1007699 (PMC6203403; doi:10.1371/journal.pgen.1007699)

Phenotype histogram and quantile-quantile plots of p-values

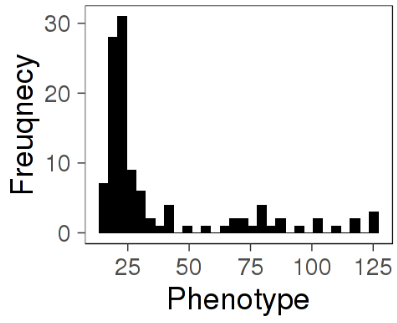

Original phenotype distribution

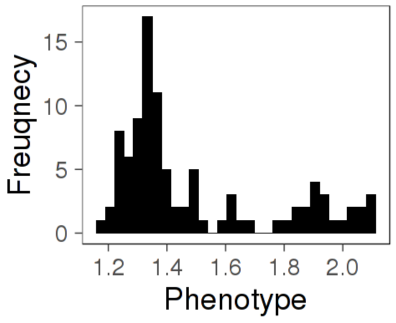

Transformed phenotype distribution

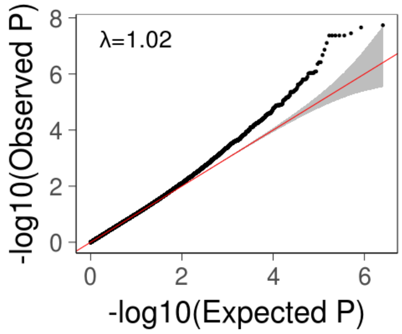

SNP

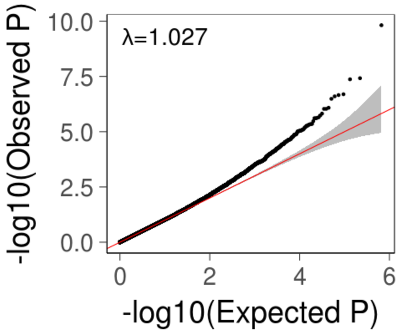

INDEL

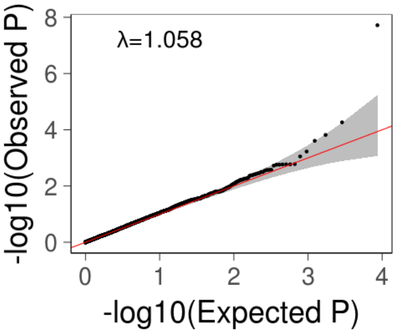

ORF

SNP results

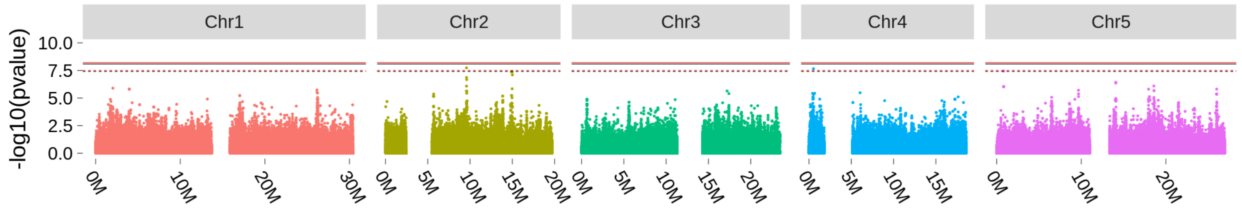

INDEL results

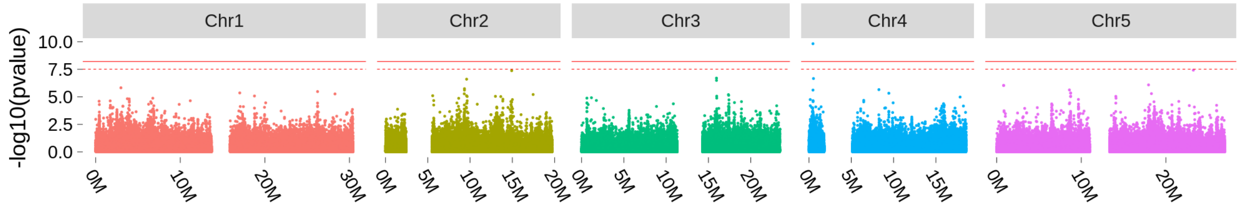

Peak	Chr	INDEL	-log10(pvalue)	Candidate	Candidate	Variation	Distance to gene(bp)

rank		pos(bp)		gene ID	gene name		
1	4	468530	9.815293	NA	NA	1bp insertion	NA

ORFS results

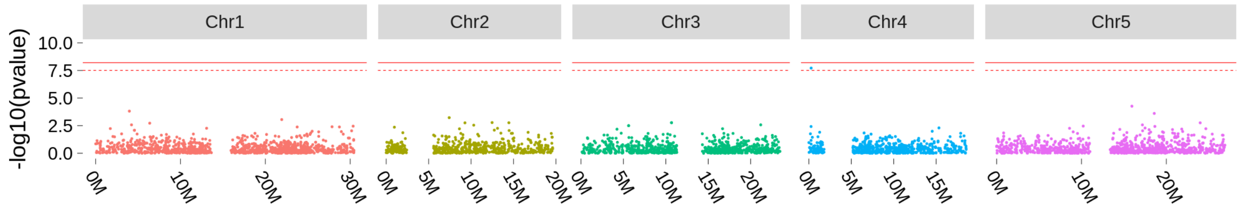

Supplement: S32 Fig — (PDF) [file pgen.1007699.s033.pdf]
