# Supplementary material for: Recovery of novel association loci in Arabidopsis thaliana and Drosophila melanogaster through leveraging INDELs association and integrated burden test
Source: PLoS Genet. 2018 Oct 16;14(10):e1007699. doi: 10.1371/journal.pgen.1007699 (PMC6203403; doi:10.1371/journal.pgen.1007699)

## Phenotype histogram and quantile-quantile plots of p-values

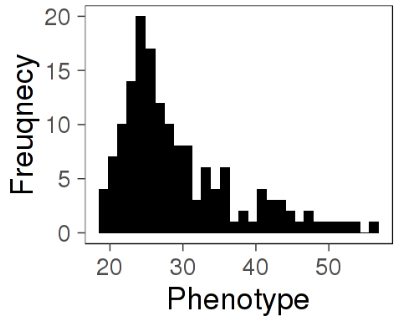


Original phenotype distribution

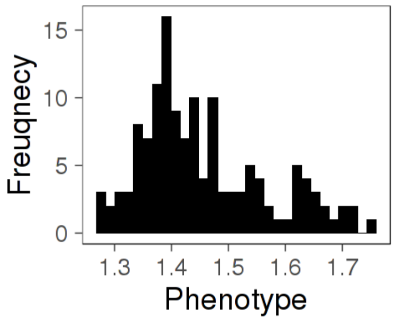


Transformed phenotype distribution

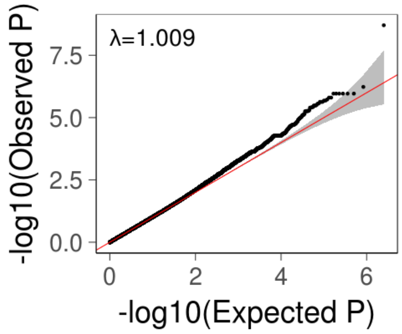


SNP

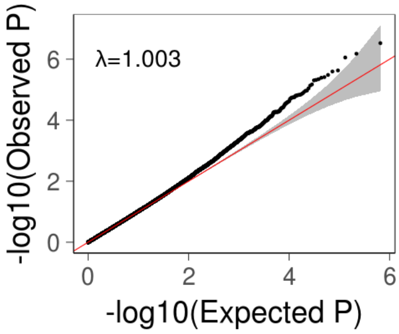


INDEL

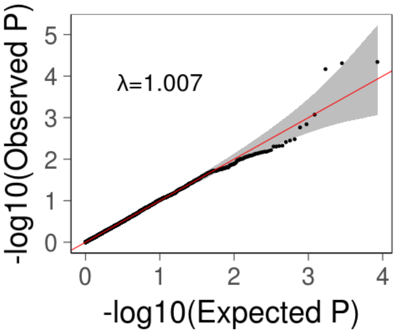


ORF

## SNP results

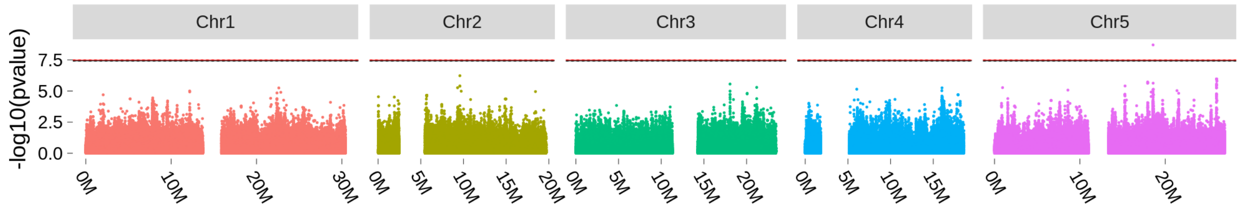


| Peak rank | Chr | SNP pos(bp) | -log10(pvalue) | Candidate gene ID | Candidate gene name | Distance to gene(bp) |
|---|---|---|---|---|---|---|
| 1 | 5 | 18576038 | 8.705972 | AT5G45830 | DOG1 | 13444 |

## INDEL results

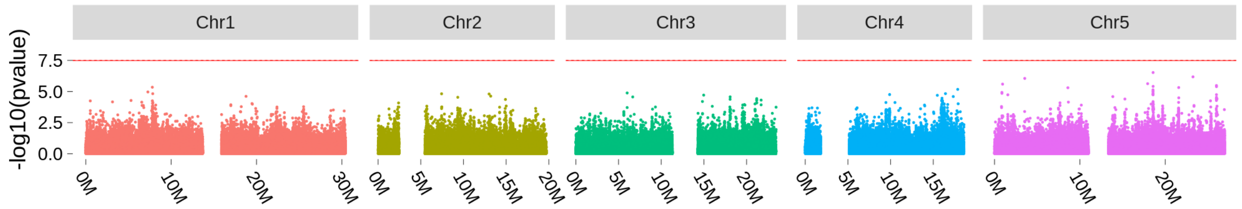

# ORFS results

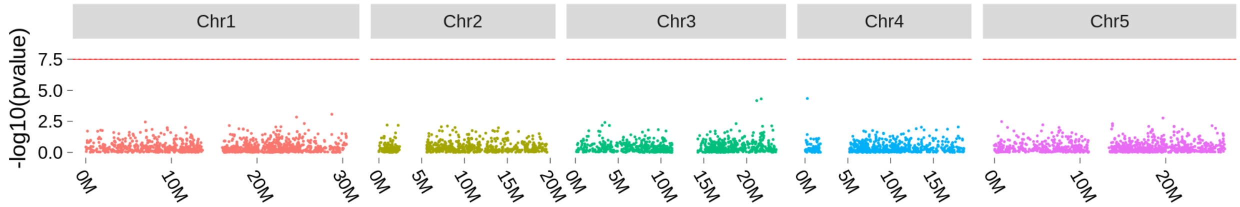

Supplement: S35 Fig — (PDF) [file pgen.1007699.s036.pdf]
