# Supplementary material for: Recovery of novel association loci in Arabidopsis thaliana and Drosophila melanogaster through leveraging INDELs association and integrated burden test
Source: PLoS Genet. 2018 Oct 16;14(10):e1007699. doi: 10.1371/journal.pgen.1007699 (PMC6203403; doi:10.1371/journal.pgen.1007699)

Phenotype histogram and quantile-quantile plots of p-values

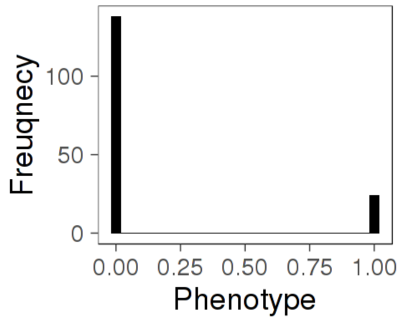

Original phenotype distribution

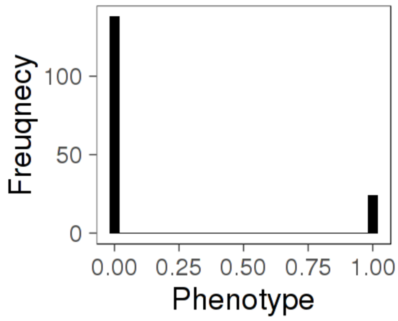

Transformed phenotype distribution

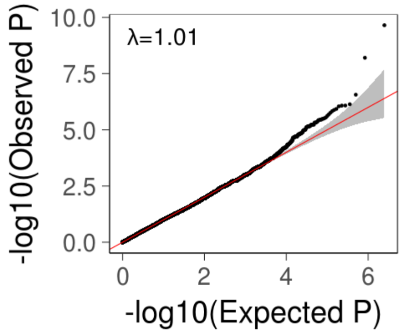

SNP

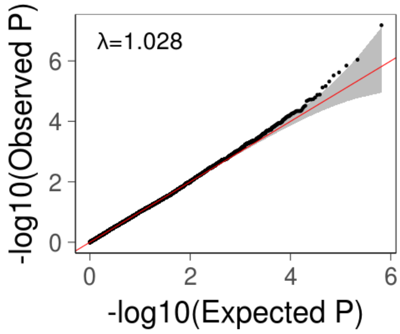

INDEL

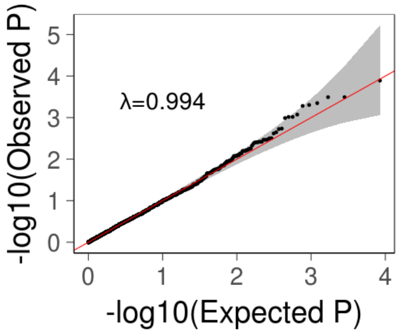

ORF

SNP results

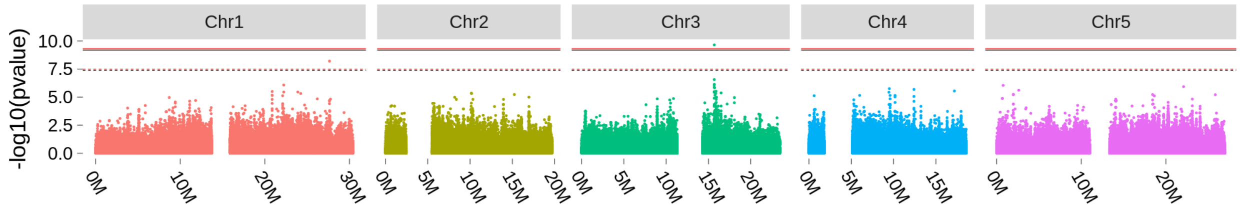

Peak rank	Chr	SNP pos(bp)	-log10(pvalue)	Candidate gene ID	Candidate gene name	Distance to gene(bp)
1	3	15692768	9.651037	NA	NA	NA

INDEL results

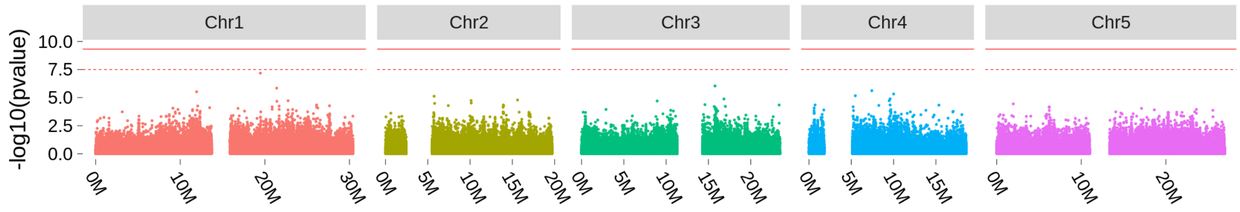

ORFS results

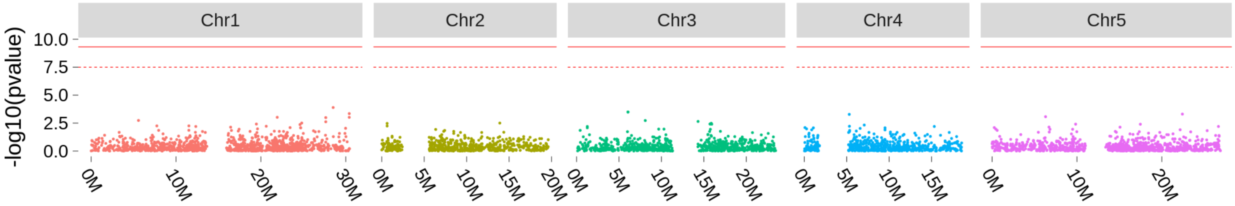

Supplement: S42 Fig — (PDF) [file pgen.1007699.s043.pdf]
