# Supplementary material for: Recovery of novel association loci in Arabidopsis thaliana and Drosophila melanogaster through leveraging INDELs association and integrated burden test
Source: PLoS Genet. 2018 Oct 16;14(10):e1007699. doi: 10.1371/journal.pgen.1007699 (PMC6203403; doi:10.1371/journal.pgen.1007699)

quantile-quantile plots of p-values

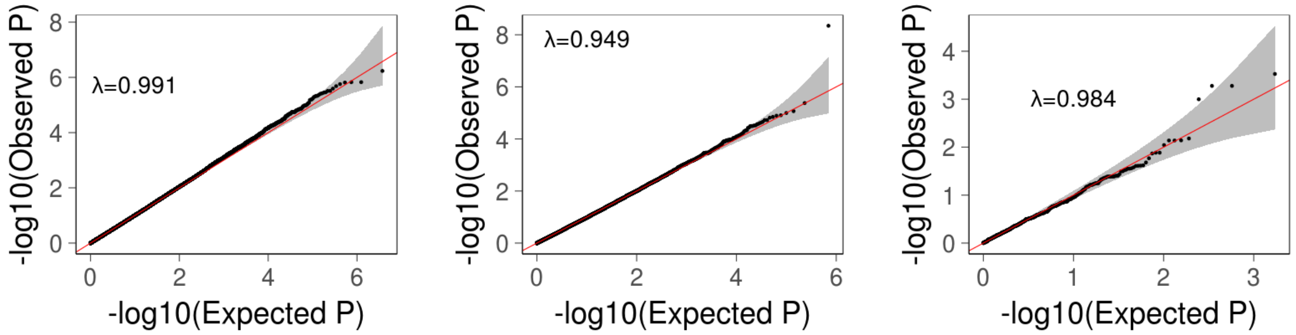

SNP INDEL ORF

SNP results

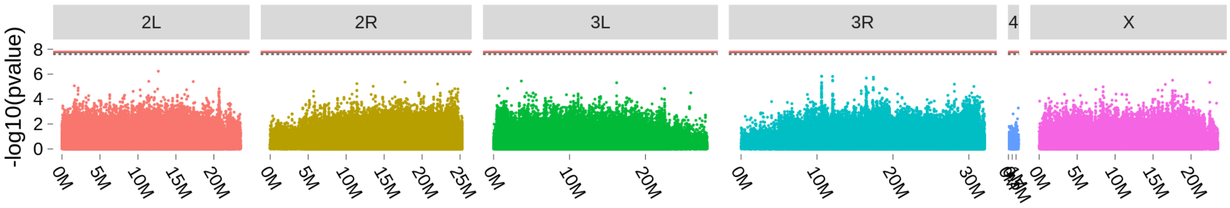

INDEL results

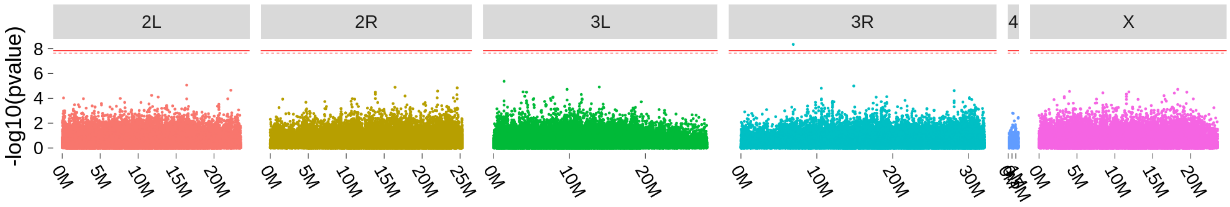

Peak rank	Chr	INDEL pos(bp)	-log10(pvalue)	Candidate gene ID	Candidate gene name	Variation	Distance to gene(bp)
1	3R	6892261	8.345634	NA	NA	12bp deletion	NA

ORFS results

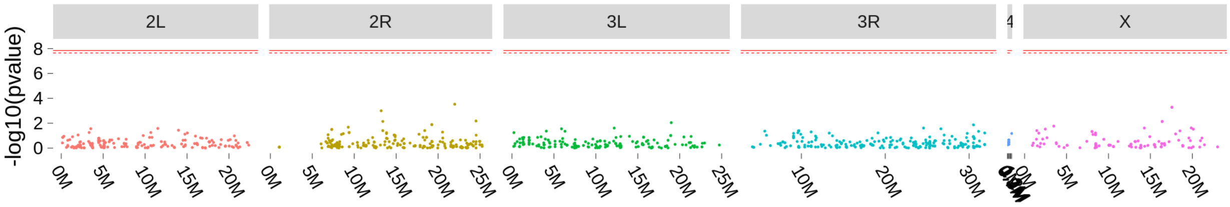

Supplement: S54 Fig — (PDF) [file pgen.1007699.s055.pdf]
