# Supplementary material for: Recovery of novel association loci in Arabidopsis thaliana and Drosophila melanogaster through leveraging INDELs association and integrated burden test
Source: PLoS Genet. 2018 Oct 16;14(10):e1007699. doi: 10.1371/journal.pgen.1007699 (PMC6203403; doi:10.1371/journal.pgen.1007699)
Supplement: S60 Fig — (PDF) [file pgen.1007699.s061.pdf]

quantile-quantile plots of p-values

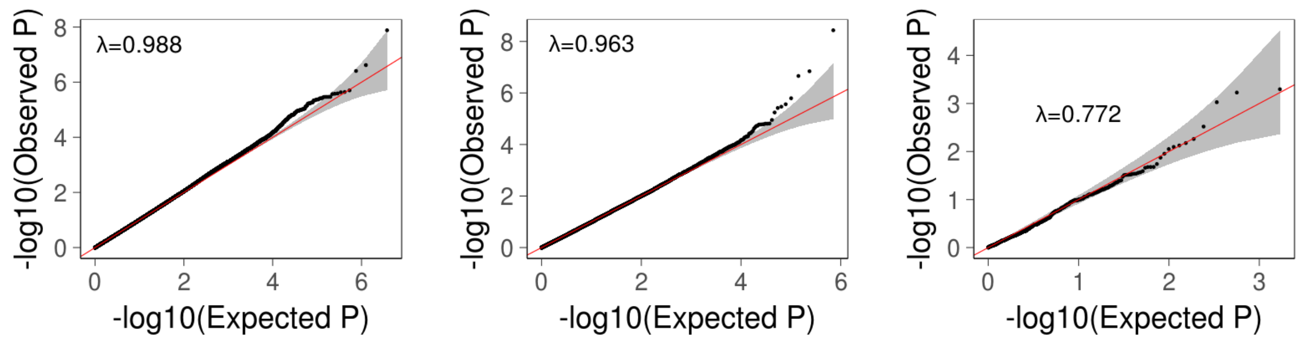

SNP INDEL ORF

SNP results

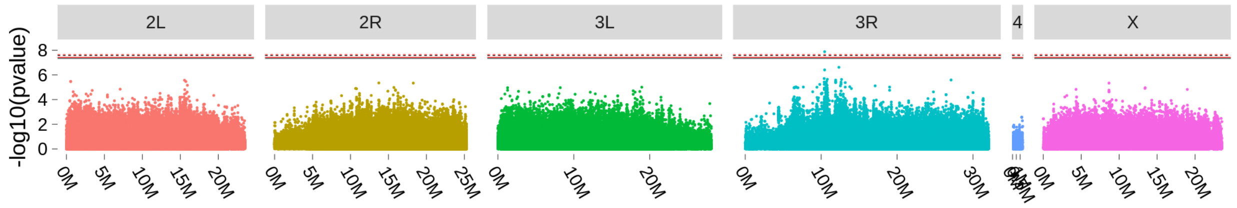

Peak rank	Chr	SNP pos(bp)	-log10(pvalue)	Candidate gene ID	Candidate gene name	Distance to gene(bp)
1	3R	10457365	7.882685	NA	NA	NA

INDEL results

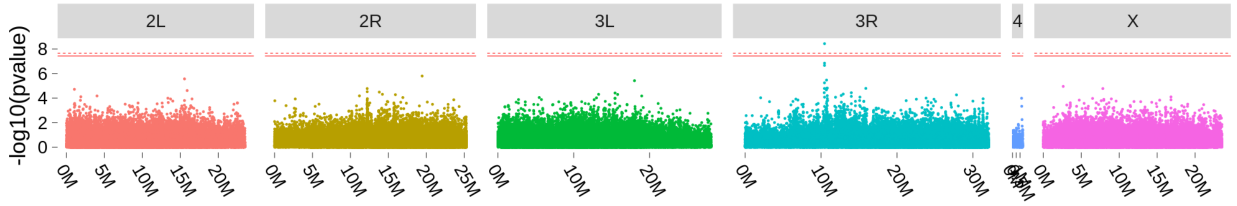

Peak rank	Chr	INDEL pos(bp)	-log10(pvalue)	Candidate gene ID	Candidate gene name	Variation	Distance to gene(bp)
1	3R	10457824	8.432678	NA	NA	1bp deletion	NA

ORFS results

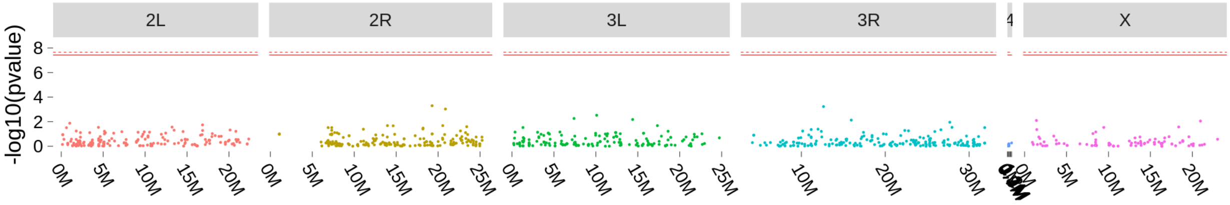
2L
2R
3L
3R
4
X
-log10(pvalue)
8
6
4
2
0
0M
5M
10M
15M
20M
25M
30M
