# Supplementary material for: Recovery of novel association loci in Arabidopsis thaliana and Drosophila melanogaster through leveraging INDELs association and integrated burden test
Source: PLoS Genet. 2018 Oct 16;14(10):e1007699. doi: 10.1371/journal.pgen.1007699 (PMC6203403; doi:10.1371/journal.pgen.1007699)

quantile-quantile plots of p-values

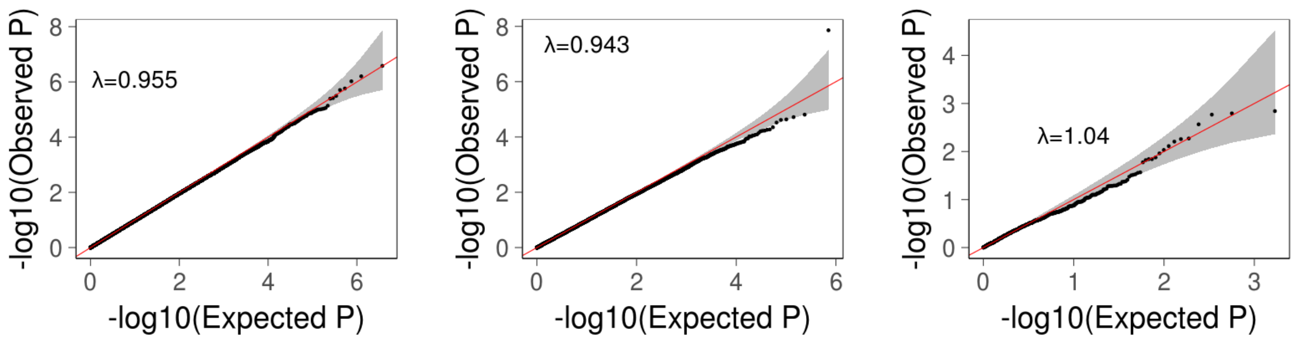


SNP INDEL ORF

SNP results

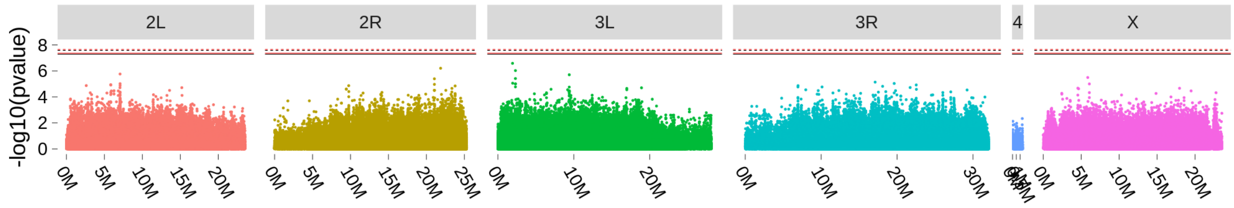


INDEL results

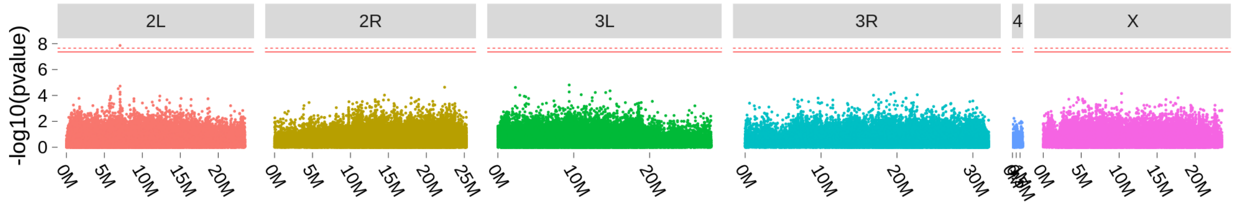


| Peak rank | Chr | INDEL pos(bp) | -log10(pvalue) | Candidate gene ID | Candidate gene name | Variation | Distance to gene(bp) |
|---|---|---|---|---|---|---|---|
| 1 | 2L | 7062316 | 7.858395 | NA | NA | 2bp deletion | NA |

ORFS results

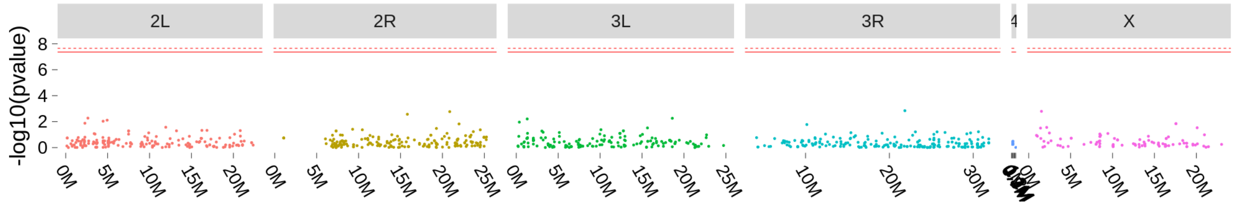

Supplement: S65 Fig — (PDF) [file pgen.1007699.s066.pdf]
