# Supplementary material for: Recovery of novel association loci in Arabidopsis thaliana and Drosophila melanogaster through leveraging INDELs association and integrated burden test
Source: PLoS Genet. 2018 Oct 16;14(10):e1007699. doi: 10.1371/journal.pgen.1007699 (PMC6203403; doi:10.1371/journal.pgen.1007699)
Supplement: S1 Table — Those in bold font are accessions whose sequence data are from an individual with same native name. (DOC) [file pgen.1007699.s071.doc]

| Phenotype ecotype ID | Genotype ecotype ID | Native name | Phenotype ecotype ID | Genotype ecotype ID | Native name | Phenotype ecotype ID | Genotype ecotype ID | Native name |
| --- | --- | --- | --- | --- | --- | --- | --- | --- |
| 5837 | 5837 | Bor-1 | 6967 | 6967 | Sq-8 | 8239 | 8239 | Köln |
| 6008 | 6008 | Duk | 6968 | 6968 | Tamm-2 | 8240 | 8240 | Kulturen-1 |
| 6009 | 6009 | Eden-1 | 6969 | 6969 | Tamm-27 | 8241 | 8241 | Liarum |
| 6016 | 6016 | Eds-1 | 6970 | 6970 | Ts-1 | 8242 | 8242 | Lillö-1 |
| 6040 | 6040 | Kni-1 | 6971 | 6971 | Ts-5 | 8243 | 8243 | PHW-2 |
| 6042 | 6042 | Lom1-1 | 6972 | 6972 | Tsu-1 | **8245** | **7332** | **Seattle-0** |
| 6043 | 6043 | Löv-1 | 6973 | 6973 | Ull2-3 | 8247 | 8247 | San-2 |
| 6046 | 6046 | Löv-5 | 6974 | 6974 | Ull2-5 | 8249 | 8249 | Vimmerby |
| 6064 | 6064 | Nyl-2 | 6975 | 6975 | Uod-1 | **8254** | **6992** | **Ang-0** |
| 6074 | 6074 | Ör-1 | 6976 | 6976 | Uod-7 | 8256 | 8256 | Bå1-2 |
| 6243 | 6243 | Tottarp-2 | **6977** | **7383** | **Van-0** | 8258 | 8258 | Bå4-1 |
| 6709 | 6709 | Bg-2 | **6978** | **7394** | **Wa-1** | 8259 | 8259 | Bå5-1 |
| 6897 | 6897 | Ag-0 | 6979 | 6979 | Wei-0 | 8264 | 8264 | Bla-1 |
| 6898 | 6898 | An-1 | **6980** | **7396** | **Ws-0** | 8265 | 8265 | Blh-1 |
| 6899 | 6899 | Bay-0 | 6981 | 6981 | Ws-2 | 8266 | 8266 | Boo2-1 |
| 6900 | 6900 | Bil-5 | 6982 | 6982 | Wt-5 | **8270** | **7003** | **Bs-1** |
| 6901 | 6901 | Bil-7 | **6983** | **7416** | **Yo-0** | **8271** | **7036** | **Bu-0** |
| 6903 | 6903 | Bor-4 | 6984 | 6984 | Zdr-1 | **8274** | **7063** | **Can-0** |
| 6904 | 6904 | Br-0 | 6985 | 6985 | Zdr-6 | 8275 | 8275 | Cen-0 |
| **6905** | **7058** | **Bur-0** | 6988 | 6988 | Alc-0 | 8283 | 8283 | Dra3-1 |
| 6906 | 6906 | C24 | 7000 | 7000 | Aa-0 | 8284 | 8284 | DraII-1 |
| 6907 | 6907 | CIBC-17 | 7014 | 7014 | Ba-1 | 8285 | 8285 | DraIII-1 |
| 6908 | 6908 | CIBC-5 | 7033 | 7033 | Buckhorn Pass | 8290 | 8290 | En-1 |
| 6909 | 6909 | Col-0 | 7062 | 7062 | Ca-0 | **8296** | **7161** | **Gd-1** |
| **6910** | **7067** | **Ct-1** | 7064 | 7064 | Cnt-1 | 8297 | 8297 | Ge-0 |
| 6911 | 6911 | Cvi-0 | 7081 | 7081 | Co | **8300** | **430** | **Gr-1** |
| 6913 | 6913 | Eden-2 | 7094 | 7094 | Da-0 | 8306 | 8306 | Hov4-1 |
| **6914** | **7111** | **Edi-0** | 7123 | 7123 | Ep-0 | **8310** | **7162** | **Hs-0** |
| 6915 | 6915 | Ei-2 | 7147 | 7147 | Gie-0 | 8311 | 8311 | In-0 |
| 6916 | 6916 | Est-1 | 7163 | 7163 | Ha-0 | 8312 | 8312 | Is-0 |
| 6917 | 6917 | Fäb-2 | 7231 | 7231 | Li-7 | **8313** | **7177** | **Jm-0** |
| 6918 | 6918 | Fäb-4 | 7255 | 7255 | Mh-0 | 8314 | 8314 | Ka-0 |
| 6919 | 6919 | Ga-0 | 7275 | 7273 | No-0 | 8323 | 8323 | Lc-0 |
| 6920 | 6920 | Got-22 | 7282 | 7282 | Or-0 | 8325 | 8325 | Lip-0 |
| 6921 | 6921 | Got-7 | 7296 | 7296 | Petergof | 8326 | 8326 | Lis-1 |
| 6922 | 6922 | Gu-0 | 7306 | 7306 | Pog-0 | **8329** | **7217** | **Lm-2** |
| 6923 | 6923 | HR-10 | 7323 | 7323 | Rubezhnoe-1 | 8334 | 8334 | Lu-1 |
| 6924 | 6924 | HR-5 | 7346 | 7346 | Ste-0 | 8335 | 8335 | Lund |
| 6926 | 6926 | Kin-0 | 7418 | 7418 | Zu-1 | 8337 | 8337 | Mir-0 |
| 6927 | 6927 | Kno-10 | 7424 | 7424 | Jl-3 | 8343 | 8343 | Na-1 |
| 6928 | 6928 | Kno-18 | 7438 | 7438 | N13 | 8351 | 8351 | Ost-0 |
| 6929 | 6929 | Kondara | 7460 | 7460 | Da(1)-12 | 8353 | 8353 | Pa-1 |
| 6930 | 6930 | Kz-1 | 7461 | 7461 | H55 | 8354 | 8354 | Per-1 |
| 6931 | 6931 | Kz-9 | 7477 | 7477 | WAR | 8357 | 8357 | Pla-0 |
| 6932 | 6932 | Ler-1 | 7514 | 7514 | RRS-7 | 8365 | 8365 | Rak-2 |
| 6933 | 6933 | LL-0 | 7515 | 7515 | RRS-10 | 8366 | 8366 | Rd-0 |
| 6936 | 6936 | Lz-0 | 7516 | 7516 | Vår2-1 | 8369 | 8369 | Rev-1 |
| 6937 | 6937 | Mrk-0 | 7517 | 7517 | Vår2-6 | **8374** | **7322** | **Rsch-4** |
| 6939 | 6939 | Mt-0 | 7518 | 7518 | ÖMö2-1 | 8376 | 8376 | Sanna-2 |
| 6940 | 6940 | Mz-0 | 7519 | 7519 | ÖMö2-3 | 8378 | 8378 | Sap-0 |
| 6942 | 6942 | Nd-1 | 7520 | 7520 | Lp2-2 | 8387 | 8387 | St-0 |
| 6943 | 6943 | NFA-10 | 7521 | 7521 | Lp2-6 | **8388** | **7347** | **Stw-0** |
| 6944 | 6944 | NFA-8 | 7522 | 7522 | Mr-0 | **8389** | **7349** | **Ta-0** |
| 6945 | 6945 | Nok-3 | 7523 | 7523 | Pna-17 | **8395** | **7375** | **Tu-0** |
| **6946** | **7288** | **Oy-0** | 7524 | 7524 | Rmx-A02 | 8411 | 8411 | Rd-0 |
| 6951 | 6951 | Pu2-23 | 7525 | 7525 | Rmx-A180 | 8412 | 8412 | Sav-0 |
| 6956 | 6956 | Pu2-7 | 7526 | 7526 | Pna-10 | 8420 | 8420 | Kelsterbach-4 |
| 6957 | 6957 | Pu2-8 | 8213 | 8213 | Pro-0 | 8422 | 8422 | Fjä1-1 |
| 6958 | 6958 | Ra-0 | 8214 | 8214 | Gy-0 | 8423 | 8423 | Hov2-1 |
| 6959 | 6959 | Ren-1 | **8215** | **9941** | **Fei-0** | 8424 | 8424 | Kas-2 |
| 6960 | 6960 | Ren-11 | 8222 | 8222 | Lis-2 | 8426 | 8426 | Ull1-1 |
| 6961 | 6961 | Se-0 | 8230 | 8230 | Algutsrum | 8430 | 8430 | Lisse |
| 6962 | 6962 | Shahdara | 8231 | 8231 | Brö1-6 | 9057 | 9057 | Vinslöv |
| 6963 | 6963 | Sorbo | 8233 | 8233 | Dem-4 | 9058 | 9058 | Västervik |
| 6964 | 6964 | Spr1-2 | 8235 | 8235 | Hod | **100000** | **8419** | **Wil-1-Dean-Lab** |
| 6965 | 6965 | Spr1-6 | 8236 | 8236 | HSm |  |  |  |
| 6966 | 6966 | Sq-1 | 8237 | 8237 | Kävlinge-1 |  |  |  |
