# Supplementary material for: Recovery of novel association loci in Arabidopsis thaliana and Drosophila melanogaster through leveraging INDELs association and integrated burden test
Source: PLoS Genet. 2018 Oct 16;14(10):e1007699. doi: 10.1371/journal.pgen.1007699 (PMC6203403; doi:10.1371/journal.pgen.1007699)
Supplement: S2 Table — (DOC) [file pgen.1007699.s072.doc]

| ecotype | ORF conserve transcript from Gan *et al*.^*^ | ORF conserve transcript | identical count | identical proportion |
| --- | --- | --- | --- | --- |
| Bur_0 | 39039 | 30618 | 30277 | 0.98886276 |
| Edi_0 | 39107 | 30696 | 30383 | 0.989803232 |
| Ler_0 | 39012 | 30609 | 30276 | 0.989120847 |
| Oy_0 | 39123 | 30717 | 30446 | 0.991177524 |
| Sf_2 | 38983 | 30526 | 30173 | 0.988436087 |
| Ws_0 | 39046 | 30573 | 30259 | 0.9897295 |
| Can_0 | 38856 | 30205 | 29824 | 0.987386194 |
| Hi_0 | 39196 | 30950 | 30610 | 0.98901454 |
| Mt_0 | 39093 | 30746 | 30427 | 0.989624667 |
| Po_0 | 39209 | 30981 | 30658 | 0.989574255 |
| Tsu_0 | 39098 | 30745 | 30466 | 0.990925354 |
| Wu_0 | 39130 | 30846 | 30536 | 0.989950075 |
| Ct_1 | 39091 | 30690 | 30384 | 0.990029326 |
| Kn_0 | 39058 | 30585 | 30260 | 0.989373876 |
| No_0 | 39035 | 30730 | 30387 | 0.988838269 |
| Rsch_4 | 39116 | 30856 | 30559 | 0.990374644 |
| Wil_2 | 38972 | 30585 | 30236 | 0.988589178 |
| Zu_0 | 39096 | 30713 | 30405 | 0.989971673 |

^*^including predicted novel transcripts, which is not included in TAIR10 annotation
