# Supplementary material for: Recovery of novel association loci in Arabidopsis thaliana and Drosophila melanogaster through leveraging INDELs association and integrated burden test
Source: PLoS Genet. 2018 Oct 16;14(10):e1007699. doi: 10.1371/journal.pgen.1007699 (PMC6203403; doi:10.1371/journal.pgen.1007699)
Supplement: S3 Table — (DOC) [file pgen.1007699.s073.doc]

|  | SNP | INDEL | ORFS | Structure Variation (length > 1kbp) |
| --- | --- | --- | --- | --- |
| *A. thaliana* | 8283590 | 3820962 | 32787 | 22808 |
| *D. melanogaster* | 5602288 | 2290193 | 22074 | 5242 |
