# Supplementary material for: Recovery of novel association loci in Arabidopsis thaliana and Drosophila melanogaster through leveraging INDELs association and integrated burden test
Source: PLoS Genet. 2018 Oct 16;14(10):e1007699. doi: 10.1371/journal.pgen.1007699 (PMC6203403; doi:10.1371/journal.pgen.1007699)
Supplement: S4 Table — Seed sowed under long day, at 21°C. The plants were moved to short day, 4°C for vernalization after 5 days, then were moved back to long day, 21°C after 14 days. (DOC) [file pgen.1007699.s074.doc]

| genetic background | days before bolting | days before bolting reach 5cm high | days before flowering |
| --- | --- | --- | --- |
| Col-0 | 38 | 41 | 41 |
| Col-0 | 38 | 41 | 41 |
| Col-0 | 38 | 41 | 41 |
| Col-0 | 38 | 41 | 41 |
| Col-0 | 38 | 41 | 41 |
| Col-0 | 38 | 41 | 41 |
| Col-0 | 38 | 41 | 41 |
| Col-0 | 38 | 41 | 41 |
| *tfl1-1* | 32 | 38 | 39 |
| *tfl1-1* | 32 | 38 | 39 |
| *tfl1-1* | 32 | 39 | 39 |
| *tfl1-1* | 32 | 39 | 39 |
| *tfl1-1* | 32 | 38 | 39 |
| *tfl1-1* | 32 | 39 | 39 |
| *tfl1-13* | 32 | 38 | 39 |
| *tfl1-13* | 32 | 39 | 39 |
| *tfl1-13* | 32 | 38 | 39 |
| *tfl1-13* | 32 | 37 | 38 |
| *tfl1-13* | 32 | 38 | 39 |
| *tfl1-13* | 32 | 38 | 39 |
