# Supplementary material for: Recovery of novel association loci in Arabidopsis thaliana and Drosophila melanogaster through leveraging INDELs association and integrated burden test
Source: PLoS Genet. 2018 Oct 16;14(10):e1007699. doi: 10.1371/journal.pgen.1007699 (PMC6203403; doi:10.1371/journal.pgen.1007699)
Supplement: S5 Table — (DOC) [file pgen.1007699.s075.doc]

| Col-0 |  |  |  | *svp* |  |  |
| --- | --- | --- | --- | --- | --- | --- |
| days before bolting | days before bolting reach 5cm high | days before flowering |  | days before bolting | days before bolting reach 5cm high | days before flowering |
| 26 | 32 | 32 |  | 18 | 23 | 23 |
| 26 | 31 | 31 |  | 17 | 22 | 24 |
| 26 | 32 | 32 |  | 18 | 22 | 24 |
| 25 | 31 | 31 |  | 18 | 23 | 23 |
| 29 | 35 | 35 |  | 18 | 22 | 22 |
| 27 | 32 | 32 |  | 17 | 22 | 22 |
| 26 | 32 | 32 |  | 17 | 22 | 23 |
| 26 | 31 | 31 |  | 16 | 20 | 21 |
| 30 | 36 | 36 |  | 21 | 27 | 28 |
| 26 | 31 | 31 |  | 18 | 22 | 23 |
| 28 | 33 | 33 |  | 18 | 24 | 24 |
|  |  |  |  | 17 | 20 | 22 |
|  |  |  |  | 18 | 22 | 22 |
