# Supplementary material for: Recovery of novel association loci in Arabidopsis thaliana and Drosophila melanogaster through leveraging INDELs association and integrated burden test
Source: PLoS Genet. 2018 Oct 16;14(10):e1007699. doi: 10.1371/journal.pgen.1007699 (PMC6203403; doi:10.1371/journal.pgen.1007699)
Supplement: S6 Table — (DOC) [file pgen.1007699.s076.doc]

| Col-0 |  |  |  | *svp* |  |  |
| --- | --- | --- | --- | --- | --- | --- |
| days before bolting | days before bolting reach 5cm high | days before flowering |  | days before bolting | days before bolting reach 5cm high | days before flowering |
| 20 | 26 | 26 |  | 14 | 18 | 20 |
| 21 | 26 | 27 |  | 14 | 17 | 19 |
| 21 | 26 | 27 |  | 14 | 18 | 20 |
| 19 | 25 | 27 |  | 15 | 19 | 21 |
| 20 | 25 | 25 |  | 14 | 17 | 20 |
| 22 | 27 | 28 |  | 14 | 18 | 21 |
| 20 | 25 | 25 |  | 14 | 18 | 20 |
|  |  |  |  | 14 | 17 | 19 |
|  |  |  |  | 15 | 20 | 23 |
|  |  |  |  | 14 | 19 | 21 |
