# Supplementary material for: Recovery of novel association loci in Arabidopsis thaliana and Drosophila melanogaster through leveraging INDELs association and integrated burden test
Source: PLoS Genet. 2018 Oct 16;14(10):e1007699. doi: 10.1371/journal.pgen.1007699 (PMC6203403; doi:10.1371/journal.pgen.1007699)
Supplement: S9 Table — (DOC) [file pgen.1007699.s079.doc]

| snpQTL | number of genes with eQTL detected | 9921 |
| --- | --- | --- |
|  | variance explained on average | 16.28% |
| indelQTL | number of genes with eQTL detected | 8558 |
|  | variance explained on average | 13.90% |
| orfsQTL | number of genes with eQTL detected | 1586 |
|  | variance explained on average | 2.02% |
| all | number of genes with eQTL detected | 10508 |
|  | variance explained on average | 17.09% |
