# Supplementary material for: Recovery of novel association loci in Arabidopsis thaliana and Drosophila melanogaster through leveraging INDELs association and integrated burden test
Source: PLoS Genet. 2018 Oct 16;14(10):e1007699. doi: 10.1371/journal.pgen.1007699 (PMC6203403; doi:10.1371/journal.pgen.1007699)
Supplement: S10 Table — (DOC) [file pgen.1007699.s080.doc]

| ecotype ID | *FRI* ORF-state | ecotype ID | *FRI* ORF-state | ecotype ID | *FRI* ORF-state |
| --- | --- | --- | --- | --- | --- |
| 7461 | shift | 7521 | conserved | 6940 | shift |
| 7177 | shift | 7000 | shift | 6924 | shift |
| 8237 | conserved | 7394 | conserved | 8230 | conserved |
| 6918 | conserved | 6931 | conserved | 7332 | shift |
| 8422 | conserved | 6927 | conserved | 8275 | conserved |
| 6921 | shift | 7460 | conserved | 6966 | shift |
| 8357 | conserved | 6898 | shift | 7306 | shift |
| 7522 | shift | 6909 | shift | 8256 | conserved |
| 6008 | shift | 7383 | conserved | 7062 | shift |
| 7347 | conserved | 8354 | conserved | 7003 | shift |
| 6040 | conserved | 7418 | shift | 7147 | shift |
| 6956 | conserved | 7036 | shift | 8243 | conserved |
| 8306 | conserved | 6901 | shift | 8420 | shift |
| 6968 | conserved | 7515 | conserved | 7162 | shift |
| 6959 | shift | 8213 | shift | 6920 | conserved |
| 6009 | conserved | 7346 | conserved | 6974 | conserved |
| 8266 | shift | 7288 | shift | 6917 | conserved |
| 8247 | conserved | 6960 | shift | 8419 | conserved |
| 6926 | shift | 8297 | conserved | 7033 | conserved |
| 6243 | conserved | 8351 | conserved | 6975 | shift |
| 6904 | conserved | 8337 | conserved | 6919 | shift |
| 6903 | conserved | 7524 | shift | 8387 | conserved |
| 8365 | shift | 8290 | shift | 6951 | conserved |
| 8334 | conserved | 5837 | conserved | 8326 | conserved |
| 6992 | shift | 7163 | shift | 6908 | shift |
| 6969 | conserved | 6932 | shift | 6933 | conserved |
| 6982 | shift | 6906 | conserved | 8239 | shift |
| 8242 | conserved | 6981 | shift | 6897 | conserved |
| 7064 | shift | 6929 | conserved | 7516 | conserved |
| 8376 | conserved | 6928 | shift | 6064 | conserved |
| 430 | shift | 6937 | conserved | 8426 | shift |
| 6943 | shift | 7323 | conserved | 6042 | shift |
| 8284 | conserved | 8335 | conserved | 7375 | shift |
| 7231 | shift | 6043 | conserved | 7296 | shift |
| 6961 | conserved | 7111 | conserved | 7014 | conserved |
| 8430 | shift | 6957 | conserved | 6016 | conserved |
| 8231 | conserved | 7063 | conserved | 6979 | shift |
| 8264 | conserved | 8240 | conserved | 8222 | conserved |
| 8353 | shift | 8424 | conserved | 6922 | shift |
| 8236 | conserved | 6963 | conserved | 6973 | shift |
| 7161 | shift | 6911 | shift | 6913 | conserved |
| 8214 | shift | 6046 | conserved | 8311 | conserved |
| 6923 | shift | 8378 | conserved | 8285 | conserved |
| 7217 | shift | 7517 | conserved | 6939 | conserved |
| 8233 | conserved | 8258 | conserved | 8259 | conserved |
| 6976 | conserved | 7282 | shift | 7416 | conserved |
| 6958 | shift | 8312 | shift | 6984 | shift |
| 7273 | shift | 7520 | conserved | 7477 | shift |
| 8369 | conserved | 7067 | shift | 6944 | shift |
| 8283 | conserved | 8343 | shift | 9941 | shift |
| 8241 | conserved | 8235 | conserved | 7322 | shift |
| 7525 | shift | 6945 | conserved | 7396 | conserved |
| 6915 | conserved | 9057 | shift | 7058 | conserved |
| 7424 | shift | 6900 | shift | 7094 | conserved |
| 6709 | shift | 8249 | conserved | 6907 | conserved |
| 7514 | shift | 6074 | conserved | 6970 | conserved |
| 9058 | conserved | 6971 | conserved | 7081 | shift |
| 8366 | shift | 7255 | shift | 6967 | shift |
| 8325 | shift | 7523 | conserved | 7349 | conserved |
